# USPPAR is a cost-effective, scalable, and highly sensitive single-cell RNA sequencing workflow compatible with diverse specimens

**DOI:** 10.1371/journal.pbio.3003537

**Published:** 2025-12-15

**Authors:** Ya-Wen Hsueh, Po-Min Chiang

**Affiliations:** 1 Institute of Clinical Medicine, College of Medicine, National Cheng Kung University, Tainan, Taiwan, R.O.C; 2 Clinical Medicine Research Center, National Cheng Kung University Hospital, College of Medicine, National Cheng Kung University, Tainan, Taiwan, R.O.C.; Institute for Systems Biology, UNITED STATES OF AMERICA

## Abstract

Single-cell RNA sequencing (scRNA-seq) requires high sensitivity, throughput, and broad compatibility across specimens. Current high-capacity methods lack sensitivity compared to low-capacity counterparts. Moreover, tissue-specific methods for collecting cells/nuclei limit unbiased comparisons across samples. Here, we propose a Unified framework by Split-Pool barcoding with optimal-efficiency PolydeoxyAdenylation for scRNA detection (USPPAR). Using short and long dsDNA substrates, low Co²⁺ concentration, while eliminating all other metal-ion components, enabled terminal deoxynucleotide transferase to efficiently polydeoxyadenylate intractable blunt and 3′ recessed dsDNA ends, which was unattainable with other systems. By benchmarking against six state-of-the-art technologies using HEK293, the efficient addition of PCR handles for cDNA amplification made USPPAR’s gene detection sensitivity comparable to high-sensitivity methods and significantly higher than existing high-cell-capacity platforms. In primary PBMCs, USPPAR enabled high-sensitivity, high-resolution scRNA-seq, and lysine conjugation improved sensitivity as an RNase inactivator. Based on nuclease reporter and mRNA protection assays, partially chelated Cu²⁺ served as a potent, non-precipitating, broad-spectrum nuclease inhibitor across various pH levels. Beyond demonstrating high sensitivity in liver tissue, an organ with low nuclease activity, single-nucleus RNA sequencing (snRNA-seq) with this inhibitor enabled one-pot extraction of RNA-stable nuclei from nuclease-rich tissues, such as the pancreas. Finally, comparisons with reference datasets from the 10× platform using mouse spleen and maize tissues showed that USPPAR matched cell-type coverage while achieving higher gene-detection efficiency. With five key enzymes available and quality-controlled, USPPAR provides a unified, cost-effective, sensitive method for high-cell-capacity scRNA profiling of diverse specimens without special equipment.

## Abstract 2

Single-cell RNA-sequencing workflows based on combinatorial barcoding are limited by their high cost and modest sensitivity. This study develops a unified single-cell RNA sequencing approach called USPPAR that combines split-pool barcoding and optimized polydeoxyadenylation, outperforming existing methods without requiring specialized equipment.

## Introduction

**Fig 1:**
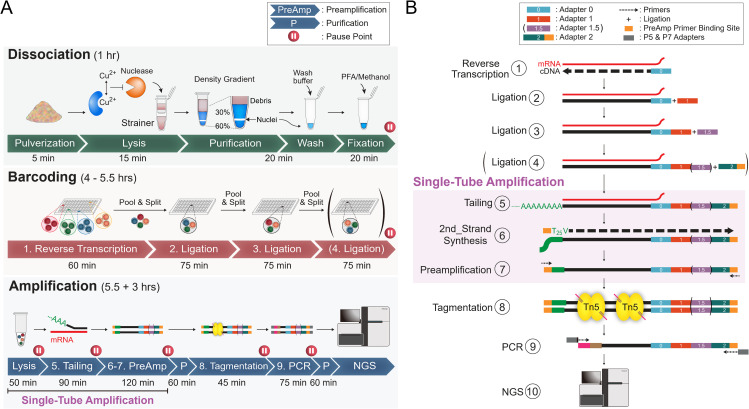
Graphic summary of this work. **(A)** The entire process is divided into three stages. **Dissociation** involves separating whole cells from culture dishes or extracting nuclei from tissues, followed by fixation. **Barcoding** includes converting mRNA to cDNA with initial tagging, then performing 2–3 additional rounds of barcoding through ligation. **Amplification** encompasses cell/nucleus lysis, high-efficiency poly(dA) extension of cDNA, preamplification, tagmentation, and final library amplification. **(B)** The corresponding cDNA structures during **Barcoding** and **Amplification**.

Single-cell RNA (scRNA)/single-nucleus RNA sequencing (snRNA-seq) workflows can be divided into three main stages: Dissociation, in which single cells are generated from cultured cells or nuclei are isolated from tissue fragments; Barcoding, performed during reverse transcription (RT) with additional rounds introduced either by ligation or by using Tn5 transposase pre-loaded with mosaic end adapters containing barcode sequences; and Amplification, in which cDNA is amplified to prepare libraries for sequencing (Fig 1, an example of the Dissociation, Barcoding and Amplification stages). Most scRNA-seq methods can be categorized by their strategies for cell Barcoding and cDNA Amplification. Focusing on barcoding, the cells could be barcoded by microfluidic chips (CEL-Seq2 [1], 10× Chromium, Drop-seq [2], inDrop [3], VASA-seq [4]), multiwell plates/microplates (QUARTZ-Seq2 [5], Microwell-Seq [6], VASA-seq [4], Smart-seq3xpress [7], FLASH-seq [8]), emulsification with barcoded beads (PIP-seq [9]), or split-pool combinatorial barcoding (SPLiT-seq [10], sci-RNA-seq3 [11]/EasySci [12,13]). The microfluidic chip, microplate, and bead-based methods require specialized equipment for their fabrication, which makes it harder to democratize to every lab. Additionally, plate/microplate-based methods and microfluidic chip-based ones, including the commercial 10× platform, only offer a lower cell input per experiment without saturating the well capacity or incurring an unacceptable collision/multiplet rate [9]. Although the limitation in capacity is resolved by recent particle-templated emulsification of PIP-seq, the nondissolvable beads and nonreleasable primers potentially reduce the capture and detection of large full-length or precursor mRNAs. The limited cell capacity, special equipment requirement, and restricted primer access are not issues with the combinatorial-barcoding of sci-RNA-seq3/EasySci and SPLiT-seq (Figs 1 and S1A; steps 1–4, example of typical split-pool barcoding). Additionally, both methods offer the benefit of multiplexing a high sample number alongside PIP-seq. Further, SPLiT-seq offers a higher potential cell capacity than sci-RNA-seq3/EasySci due to its use of more than three layers of barcoding. Thus, barcoding cells through split-pool cDNA synthesis and ligation, like SPLiT-seq, is a good option to process large cell numbers in a single experiment. However, we found that current high cell-throughput methods, including SPLiT-seq, sci-RNA-seq3/EasySci, and PIP-seq, suffered from lower gene detection sensitivity per barcode.

In addition to adding sequencing-primer binding sites, the Amplification stage (Figs 1 and S1A; steps 5 onward) serves to increase the amount of cDNA generated during the Barcoding stage, thereby improving gene-detection sensitivity. However, complete cDNA amplification is essential for achieving high detection sensitivity but remains an under-characterized problem with current scRNA-seq methods. The cDNA amplification requires a PCR handler on RT primers and the other based on the following strategies: RT after in vitro transcription (as seen in CEL-Seq2, inDrop, VASA-seq), tagmentation after second-strand synthesis (as in sci-RNA-seq3), template-switching (as in 10× Chromium, Drop-seq, Microwell-Seq, PIP-seq, SPLiT-seq, FLASH-seq, Smart-seq3xpress), or homopolymers (QUARTZ-Seq2). Strategies requiring intermediate steps of in vitro transcription (PIP-seq), second-strand synthesis (sci-RNA-seq3), or purifications (SPLiT-seq) before cDNA amplification inevitably increase technical uncertainty and incomplete conversion risk. The single-step addition of the second PCR handler through template-switching or homopolymerization, followed by purification-free amplification with techniques like FLASH-seq and QUARTZ-Seq2, maximizes detectable cDNA fragments. However, template-switching is not 100% efficient due to potential non-dC nucleotide additions to cDNA tails [14]. Homopolymerization using TdT is incomplete with recessed-end DNAs [15], like incompletely extended cDNAs on mRNAs. Furthermore, the efficiencies of tailing DNA substrates as PCR primer-binding handlers using these systems have not been directly demonstrated. Thus, our first objective is to develop and validate a reaction system that can efficiently tail challenging DNA substrates, serving as PCR handles to enable purification-free cDNA amplification (Figs 1 and S1A, steps 5–7).

Regarding the Dissociation stage, obtaining high-quality scRNA data from diverse tissues and organs of multicellular organisms remains a challenging yet underappreciated issue. Unlike cell lines or a limited number of samples such as peripheral blood, most tissues consist of cells embedded interacted with varied extracellular matrix compositions and RNase-laden body fluids, such as plasma [16]. While it’s feasible to dissociate samples for intact cell profiling, tissue-dependent procedures [17] introduce unavoidable artifacts, hindering cross-tissue comparisons of the same cell types. In addition, the dissociation potentially leads to biased release of different cell types, and the ex vivo incubation is also associated with cell state changes [18]. Given the proper sensitivity and the ability to classify cell type using nuclei as input [19], directly isolating nuclei from pulverized tissues can circumvent the issues associated with cell dissociation mentioned earlier. This approach also substantially expands the acceptable sample range. However, the abundant RNases in certain tissues, such as the blood-rich spleen or exocrine pancreas, will quickly degrade mRNAs in the nuclei within the lysate. This difficulty is evident from the absence of one-pot lysis datasets for the spleen [20–24] and the need for a very low pH buffer for human pancreatic acinar cells [25]. The extensive variation in tissue- or sample-specific transcriptome collection methods introduces additional artifacts that inevitably hinder cross-dataset integration. Thus, another important objective of this study is to develop a streamlined nuclear extraction procedure, consisting of defined steps with limited parameter variations, that enables rapid and efficient isolation of mRNA-containing nuclei from even the most challenging RNase-rich tissues.

To address the first objective, we engineered and validated a complete cDNA tailing process with single-tube amplification to engineer a scRNA-seq platform (Unified framework by Split-Pool barcoding with optimal-efficiency PolydeoxyAdenylation for scRNA detection [USPPAR]) to achieve a higher sensitivity, surpassing current high cell-throughput methods. This terminal deoxynucleotide transferase (TdT)-based, trace-free, minimal cationic system achieves complete tailing efficiency on DNA ends that are inaccessible under other reaction conditions. Together with multilayer split-pool barcoding for maximum cell capacity, the scRNA-seq based on the improved tailing was benchmarked against current platforms, demonstrating high concordance of gene expression and cell clustering, high sensitivity, low dropout rates, and minimal equipment needs. Regarding the second objective on RNase inhibition, in addition to covalent modification of lysine residues, we found that Cu^2+^ ions subjected to partial chelation could protect RNA from degradation without causing undesirable protein aggregation in nuclease-rich tissue lysates. Using this Cu^2+^-based inhibitor, our system successfully collected comprehensive cell mixture from the nuclease-rich pancreas and spleen in a one-pot lysis procedure, which had previously been impossible for the spleen [20–24]. In addition to demonstrating the coverage and cross-organ proximities of the same cell types in murine liver, pancreas, and spleen, our system was extended to include cell wall-containing maize tissues, showing improved sensitivity and cell-state co-segregation against a reference dataset prepared using 10× Chromium. Together with the open-source, high-capacity, and high-sensitivity scRNA-seq system, this one-pot nuclear extraction and stabilization, regardless of sources, provides an end-to-end toolkit to comprehensively profile cell states for unbiased cross-tissue comparisons.

## Results

### Optimizations for a self-sufficient and widely accessible system

In our three-stage scRNA-seq workflow, the first stage, Dissociation, involves dissociating and fixing/permeabilizing cells from in vitro cultures or single cells in vivo (such as peripheral blood mononuclear cells, PBMCs), or extracting nuclei from solid tissues. The second stage, Barcoding, involves synthesizing cDNA and adding the first barcode during RT, followed by 2–3 additional rounds of barcode addition through ligation. The final Amplification stage adds a poly(dA) PCR handle to the cDNA, followed by suppressive-PCR amplification. The purified PCR product is then tagmented by Tn5, followed by an additional PCR to generate the final sequencing library. (Figs 1: overview of the method; S1A: stepwise sequence modification; S1B: full library structure and sequencing strategy). These three stages are designed as independent modules that could be flexibly adapted or interchanged with corresponding components from other platforms.

Our first objective is to make this scRNA-seq system an affordable, open-source platform that is both convenient and broadly accessible to the research community. As for the Barcoding stage (Fig 1, Barcoding), although the split-pool strategy allows for high cell capacity, a major bottleneck to its widespread use is the cost associated with 3–4 rounds of barcoding. This high cost arises from the large quantities of commercial recombinant proteins required for RNase inhibition, RT, and ligation, as well as the modified oligos used in 96-well plates. Thus, one objective was to make the entire system self-sufficient and easily accessible to a wide range of users. First, direct synthesis of phosphorylated oligos was replaced by enzymatic phosphorylation using recombinant T4 polynucleotide kinase (PNK). Using this recombinant PNK to phosphorylate an unmodified oligo (S2A Fig, lane 2, arrow), we were able to subsequently ligate this oligo for complete upshifting (S2A Fig, lane 4, arrowhead), without any remaining unligated substrate (S2A Fig, lane 4, arrow). This experiment verified the efficiencies of both homebrew T4 PNK and DNA ligase. Additionally, the working oligo-modifying PNK and DNA ligase were further applied to the oligos/adapters used in the 3-round split-pool barcoding system (S1 Table, nucleotide sequences of adapters 0, 1, and 2 and corresponding splinters) using a mixed incubation experiment. The pBC0, pBC1, and BC2 represent the mock oligos/adapters used for the 1st (RT), 2nd (ligation), and 3rd (ligation) rounds of barcoding, respectively. The formation of ligation products pBC0-pBC1 (S2B Fig, lane 7, asterisk) and pBC1-BC2 (S2B Fig, lane 8, asterisk) showcased the functioning of the barcoding system. Furthermore, the absence of the pBC1 donor strand after ligation (S2B Fig, lane 8, arrow) again indicated successful oligo phosphorylation followed by ligation. The sufficient ligation using shorter 8-bp protrusions (S2A and S2B Fig), instead of the original 15-bp with SPLiT-seq, enables more layers of barcoding. Indeed, beyond the 3-layer barcoding, a 4-layer barcoding system (96^4 barcodes for unambiguously labeling more than 1 million cells) using 8-bp protrusions was demonstrated to work based on maize tissues (Fig 8; S2 Table, SampleID 6 and 7).

**Fig 2:**
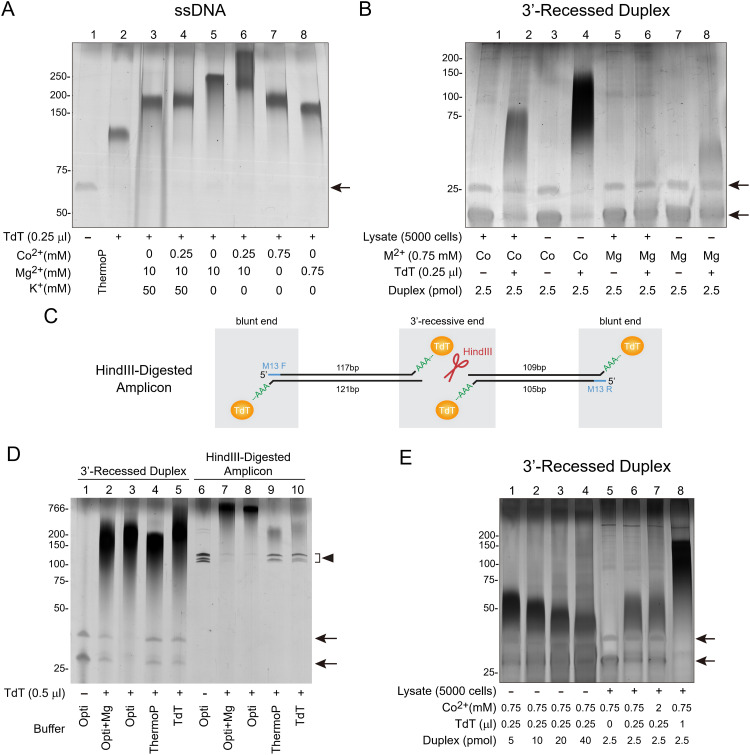
A minimal-salt, high dATP, and TdT reaction system engineering to fully tail cDNA for single-tube preamplification (STA). **(A)** Silver staining of TBE-PAGE (15%) for TdT activities in various reaction conditions. 0.5 pmole of an ssDNA oligo (GTGGAAAGGACGAAACACCGGATGCTTCCTTTTAAACAGGGTTTTAGAGCTAGAAATA) was incubated in 10 μL volumes of various compositions plus dATP (2 mM) and commercial TdT (0.25 μL) at 37, 45, 55, 65, 75°C for 30, 5, 5, 5, 20 min, respectively. All the reaction was used for electrophoresis. ThermoP: ThermoPol Reaction Buffer; Co^2+^: cobalt chloride; Mg^2+^: magnesium acetate; K^+^: potassium acetate. Tris-acetate pH 7.9 (20 mM) and Triton X-100 (0.1%) were included in the reactions of lanes 3–8. **(B)** Silver staining of TBE-PAGE (20%) for the effects of divalent cations and substrate amounts on tailing a recessed-end DNA substrate. 2.5 pmole of the substrate was incubated in 10 μL of reaction mixtures containing PMSF (0.5 mM), dATP (2 mM), Triton X-100 (0.1%), Tris-acetate pH 7.9 (20mM), and either Mg^2+^ or Co^2+^ in chloride salt form (M^2+^, 0.75 mM) as tailing cofactors, with (+) or without (−) of TdT (0.25 μL) at 37°C for 60 min followed by 75°C for 20 min. Subsequently, all the reactions were loaded for electrophoresis. The 5,000 cell-lysate (Lysate+) and the lysis buffer-only (Lysate−) serve as controls for the presence and absence of background DNA ends, respectively. **(C)** Schematic representation of the HindIII-digested PCR product, which contains both 3′-recessed and blunt ends, used to assay TdT tailing in different buffers as shown in **(D)**. The multiple-cloning site of pBluescript KS(+) was amplified using M13 F and R primers with KAPA HiFi DNA polymerase. The purified amplicon, which has blunt ends due to the exonuclease activity of the high-fidelity DNA polymerase, was then digested with the restriction enzyme HindIII to yield 3′-recessed ends in the middle. **(D)** Silver staining of denaturing 15% TBE-PAGE for superior polydeoxyadenylation of blunt and 3′-recessed ends with different reaction systems. The substrates were derived from either the annealed DNA duplex (3′-Recessed Duplex) or the purified long dsDNA shown in **(C)** (HindIII-Digested Amplicon). The annealed duplex (2.5 pmol) or HindIII-digested products (12.5 ng) were incubated in 10 μL reaction mixtures containing dATP (2 mM), TdT (0.5 μL), and one of the following buffers: optimized buffer (Opti; 20 mM Tris-acetate pH 7.9, 0.75 mM CoCl_2_, 0.1% Triton X-100), optimized buffer with an additional 0.75 mM MgCl_2_ (Opti+Mg), ThermoPol buffer (ThermoPol, 20 mM Tris-HCl pH 8.8, 10 mM (NH_4_)_2_SO_4_, 10 mM KCl, 2 mM MgSO_4_, 0.1% Triton X-100), or current standard TdT extension buffer (TdT, 20 mM Tris-acetate pH 7.9, 50 mM potassium acetate, 10 mM magnesium acetate, 0.25 mM CoCl_2,_ 0.1% Triton X-100). The reactions were performed at 37°C for 60 min, followed by 75°C for 20 min. Subsequently, half the reactions were loaded for electrophoresis. Arrowheads indicate the unextended HindIII-digested PCR products. **(E)** Silver staining of TBE-PAGE (20%) for the amounts of TdT, substrates, and cofactors on the tailing efficiency. Various pmol of the 3′ recessed-end substrate were incubated in 10 μL of reactions containing PMSF (0.5 mM), Tris-acetate pH 7.9 (20 mM), Triton X-100 (0.1%), dATP (2 mM), CoCl_2_ (0.75 or 2 mM), and commercial TdT (0, 0.25 or 1 μL) with (+) or without (−) the 5000-cell lysate (Lysate) at 37°C for 60 min followed 75°C for 20 min before loading all of reactions for electrophoresis. The recessed-end dsDNA substrate in **(B)**, **(D)**, and **(E)** and the 5,000-cell lysate used in **(B)** and **(E)** were prepared as in S2F Fig. PMSF served to quench the proteinase-K activity in **(B)** and **(E)**. Arrows denote the untailed oligo substrates in **(A)**, **(B)**, **(D)**, and **(E)**. The uncropped images of denaturing PAGE for panels **(A)**, **(B)**, **(D)**, and (E) can be found in S1 Raw Images.

**Fig 3:**
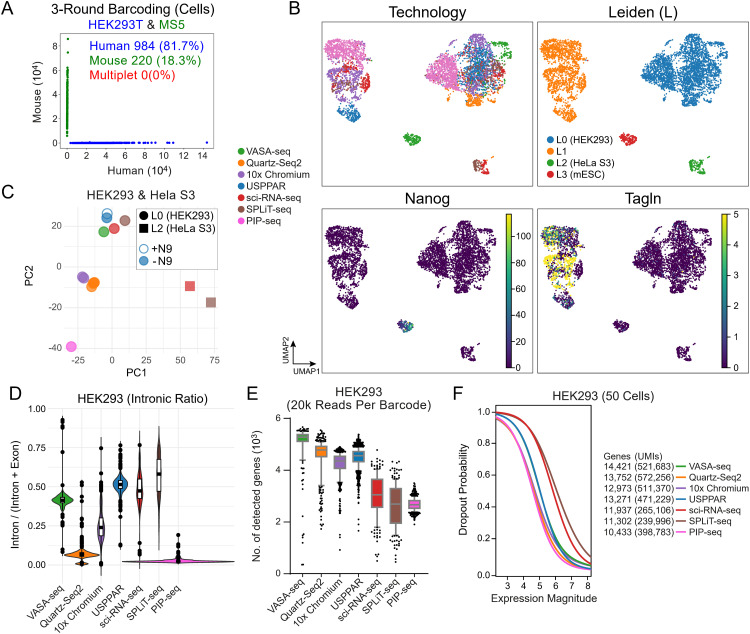
The libraries prepared using the optimized TdT tailing system enabled single-cell RNA-seq (scRNA-seq) with high gene detection sensitivity, surpassing that of current high-throughput methods. **(A)** Scatter plot of reads detected in a mixed-species experiment. Human HEK293T (70,000) and mouse MS5 (30,000) cells were dissociated, washed, and fixed in 10× volumes of methanol at −20°C before collection for USPPAR. The mixed cells underwent 3 rounds of barcoding. 1,500 cells were used for library preparation in 3 batches of approximately 500 cells. 1,204 cells (with gene counts >400) are assigned as human (blue), mouse (green), or multiplet (red) if they contain > 90% of reads from the respective species or none from either. **(B)** Joint UMAP of cells from USPPAR (1,204 HEK293 and MS5 cells), Quartz-Seq2 (327 HEK293 cells), VASA-seq (350 HEK293T and mouse embryonic stem cells), SPLiT-seq (586 HEK293, HeLa S3, and NIH/3T3 cells), 10× Chromium (982 HEK293T and NIH/3T3 cells), sci-RNA-seq (334 HEK293T, HeLa S3, and NIH/3T3 cells) and PIP-seq (1,500 HEK293T and NIH/3T3 cells). Cells containing more than 500 genes per cell, and the top 2,500 highly variable genes are included for embedding modeling [28]. The cells are colored by technologies (Technology), Leiden clustering (Leiden), or the raw expression levels of murine pluripotent (Nanog) and mesenchymal (Tagln) [29] markers. **(C)** Scatter plot of principal component analysis (PCA) showing technique- and cell type-dependent associations based on pseudobulk expression values per group. The UMI counts from subsampled (20,000) reads of Leiden 0 (HEK293) and Leiden 2 (HeLa S3) cells in (B) underwent regularization and log transformation before PCA and plotting. Colors denote technology; circles and squares Leiden 0 and 2 cells, respectively; blue open and solid circles USPPAR with (+N9) and without (−N9) random nonamers during RT (S2K Fig), respectively. **(D)** Violin and box plots of the percentages of intronic UMI over all UMI counts of individual HEK293 cells (Leiden 0) in **(B)**. The horizontal bars denote the upper-quartile, median, and lower-quartile values descendingly in each box plot. **(E)** Boxplot of the numbers of genes detected at 20,000 subsampled reads per HEK293 cell (Leiden0) in **(B)**. The horizontal bars denote the maximum, upper-quartile, median, lower-quartile, and minimum values descendingly in each box plot. **(F)** Dropout probabilities by expression magnitude. The HEK293 cells in **(E)** are randomly sampled (50) for the analysis. The numbers of genes and UMIs of the cells in the filtered count tables (minimum 500 genes per cell, minimum 1 UMI per gene, genes must be present in at least 1 cell) are shown on the right to demonstrate the difference in the numbers of UMIs/genes covered across 50 cells for different technologies. Raw count data are available at GEO (GSE256014); analysis and plotting code are available at Zenodo (https://zenodo.org/records/13927875).

**Fig 4:**
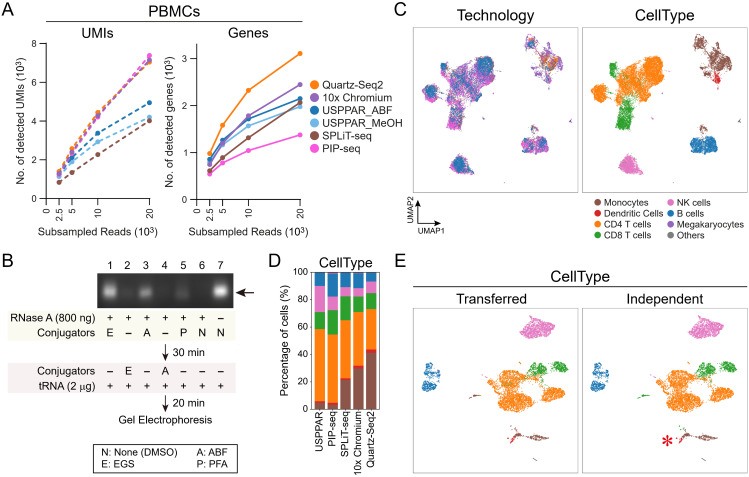
USPPAR profiling of PBMCs with enhanced sensitivity via lysine conjugation. **(A)** Medians of detected UMIs (UMIs) and genes (Genes) at different subsampled read depths per PBMC across technologies shown in **(C)**. PBMCs were purified via Ficoll-Paque and methanol-fixed for the Barcoding and Amplification procedure, as with HEK293T cells in Fig 3 (USPPAR_MeOH). For USPPAR_ABF, methanol-fixed cells were treated with 1 mM ABF prior to the Barcoding stage. **(B)** 2% agarose gel electrophoresis with fluorescent dye staining showing RNase A inhibition by different conjugators. 800 ng of RNase A in 8 µL of HEPES (pH 7.2) was mixed with 1 µL of 10% PFA (P), 100 mM EGS (E), 10 mM ABF (A), or DMSO (N) and incubated at ambient temperature for 30 min. Yeast tRNA (2,000 ng) was added to the reaction mixtures, which were incubated for 20 min before loading onto an agarose gel. The delayed addition of EGS and ABF in lanes 2 and 4 indicates that the two modifiers were added just before tRNA. **(C)** Joint UMAPs of PBMCs from USPPAR, PIP-seq V, SPLiT-seq (Evercode v3), and 10× Chromium (GEMX-v4) (8,176 cells each), and Quartz-Seq2 (1,006 cells). Cells containing 500–10,000 genes per cell were included for embedding modeling. The embedding was computed using the top 6,000 highly variable genes identified across the combined datasets. Cells are colored by technology (Technology) or by cell-type annotation (CellType) using high-resolution Leiden clusters [30]. **(D)** Stacked bar plots showing the percentages of annotated cell types across technologies in **(C)**. Bars are ordered from left to right by Euclidean distance to the USPPAR group. **(E)** UMAPs of 8,176 USPPAR PBMCs modeled alone, colored by cell-type annotations. Cell types were either transferred from the joint model in **(C)** (Transferred CellType) or derived from high-resolution Leiden clustering of the USPPAR-only model (Independent CellType). Asterisk indicates a small dendritic cell cluster correctly annotated using the independent model. Raw count data are available at GEO (GSE256014) and code for analysis and plotting at Zenodo (https://zenodo.org/records/13927875) for panels **(A)** and **(C)**–**(E)**. Uncropped images of the agarose gel **(B)** are in S1 Raw Images.

**Fig 5:**
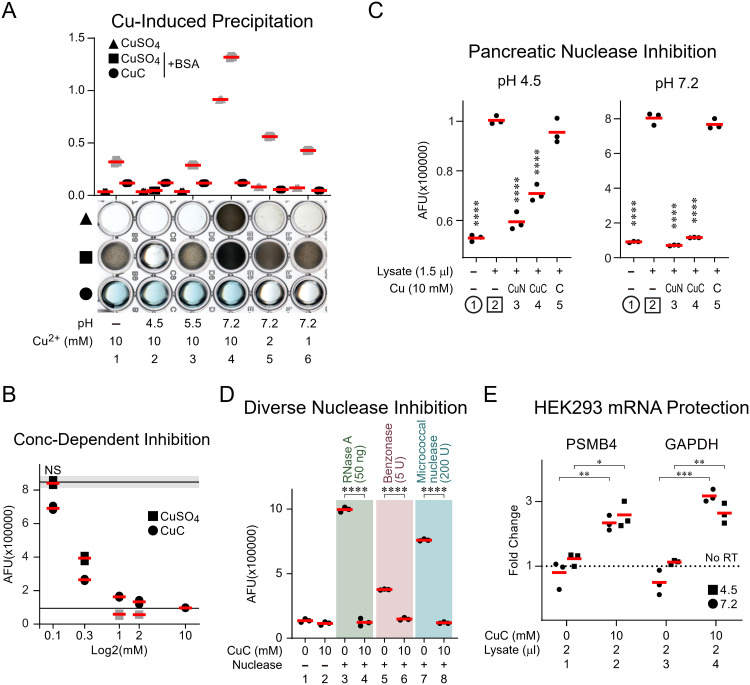
Cu²⁺, with partial coordination site occupancy, served as a soluble, potent, broad-spectrum nuclease inhibitor without causing protein coagulation at acidic to neutral pH levels. **(A)** Photograph (lower) and corresponding scatter plots (upper) showing Cu² ⁺ precipitation under different pH levels, with and without the presence of albumin and a chelator. 180 μL solutions, either unbuffered or buffered with various 100 mM buffer agents, were mixed with CuSO₄ or CuSO₄ complexed 1:1 molar with citric acid (CuC) at final concentrations of 10, 2, or 1 mM. The diluted CuC was prepared from 250 mM stock solutions: pH 4.5 for the first three lanes and pH 7.2 for the remaining three to minimize pH drift. Bovine serum albumin (BSA, final concentration: 10 μg/mL) was added as indicated. The mixtures were then aliquoted and incubated at ambient temperature for photographing (lower) and OD600 measurement (upper). Triangles: CuSO₄ alone; squares: CuSO₄ + BSA; circles: CuC + BSA. Buffers: pH 4.5 (HEPES-SO₄), pH 5.5 (MES), pH 7.2 (HEPES). The gray symbols in the scatter plot indicate visible precipitation. **(B)** Scatter plot showing the inhibition of green fluorophore release by pancreatic nucleases in the presence of Cu²⁺ or CuC. The nuclease-cleavable oligo (50 nM) was prepared in 25 μL of reaction buffer (50 mM HEPES pH 7.2, 146 mM NaCl, 1 mM CaCl₂, 21 mM MgCl₂, with 0.1% each of TWEEN-20 and NP-40, and 0.01% digitonin). It was supplemented with 1.5 μL of pancreatic lysate (except in the negative control) and the specified additives (CuSO₄ or CuC at various concentrations). After incubating at ambient temperature for 30 min, the release of the fluorophore due to cleavage was quantified using the green fluorescent channel. The upper and lower horizontal black lines represent the means, with gray shading around each line indicating the standard error of the mean for the Cu²⁺-free (positive) and Cu²⁺-free/lysate-free (negative) controls, respectively. All data points, except the square labeled NS, showed *p* < 0.0001 based on one-way ANOVA with Dunnett’s post hoc test, using the upper horizontal line as the reference. **(C)** Scatter plot showing the inhibition of pancreatic nucleases by CuC or chelator alone at acidic (4.5) and neutral (7.2) pH levels. The same basal reaction buffer as in **(B)**, with two different pH levels—HEPES-SO₄ at pH 4.5 or HEPES at pH 7.2—and containing the specified additives (pancreatic lysate; CuC; CuSO₄ complexed 1:1 molar with NTA, denoted as CuN; or citric acid at respective pH levels, denoted as **C)**. The incubation and quantification were performed identically to **(B)**. Lanes 2 (in squares): positive control for reference in one-way ANOVA with Dunnett’s post hoc tests; Lanes 1 (in circles): lysate-free negative control. **(D)** Scatter plot showing the inhibition of various nucleases by CuC. The same basal reaction buffer as in **(B)** was used with the following conditions: no addition, CuC only, RNase A, RNase A + CuC, Benzonase, Benzonase + CuC, Micrococcal nuclease, and Micrococcal nuclease + CuC. After 10-minute incubation at ambient temperature, the quantification was performed identically to **(B)**. Lanes 3, 5, and 7 serve as the reference for Student’s t-tests, with lanes 4, 6, and 8 as the test conditions. **(E)** RT-qPCR for demonstrating the protection of mRNA from pancreatic nucleases by CuC. Thirty μL of pH 4.5 and 7.2 lysis buffers as in **(C)**, containing 1× Halt proteinase inhibitor, 2 μL of pancreatic lysate, and either 0 mM (control) or 10 mM CuC (from 250 mM stock solutions, adjusted to pH 4.5 and 7.2, respectively), were prepared on ice. 20,000 HEK293T cells in 2 μL of PBS were added to the lysis buffer, and the nuclei were released by rotating the cells in the fridge for 15 min. The nuclear pellets were obtained by centrifugation in a swing-bucket rotor at 1,000*g* for 1 min, washed twice with 100 μL of the same buffers excluding Halt, with the detergent in the wash buffer removed and replaced with 0.05% PVA. After lysing the nuclear pellets in 15 μL of mRNA lysis buffer, the entire lysate was used for reverse transcription (20 μL reactions) and qPCR (20 μL reactions in triplicate). The Y-axis shows the relative enrichment of cDNA with reverse transcriptases against the no-enzyme control. Primers targeting PSMB4 and GAPDH exons and VEGFA promoter regions served to amplify the cDNA and reference genomic DNA, respectively. Squares indicate the lysis buffer at pH 4.5, and circles indicate pH 7.2. Student’s t-tests were conducted to compare each condition with and without CuC. The fluorescent quantifications in **(B)**, **(C)**, and **(D)** were diluted to 150 μL with the respective reaction buffers, excluding the substrate oligo, nucleases, and pancreatic lysate, before fluorescence measurements in triplicate. Individual replicate values are shown as filled shapes (gray or black), and means are indicated by red horizontal lines in the scatterplots **(A)**–**(E)**. *, **, ***, and **** denote *p* < 0.05, *p* < 0.01, *p* < 0.001, and *p* < 0.0001, respectively. Numerical data are available in S1 Data.

**Fig 6:**
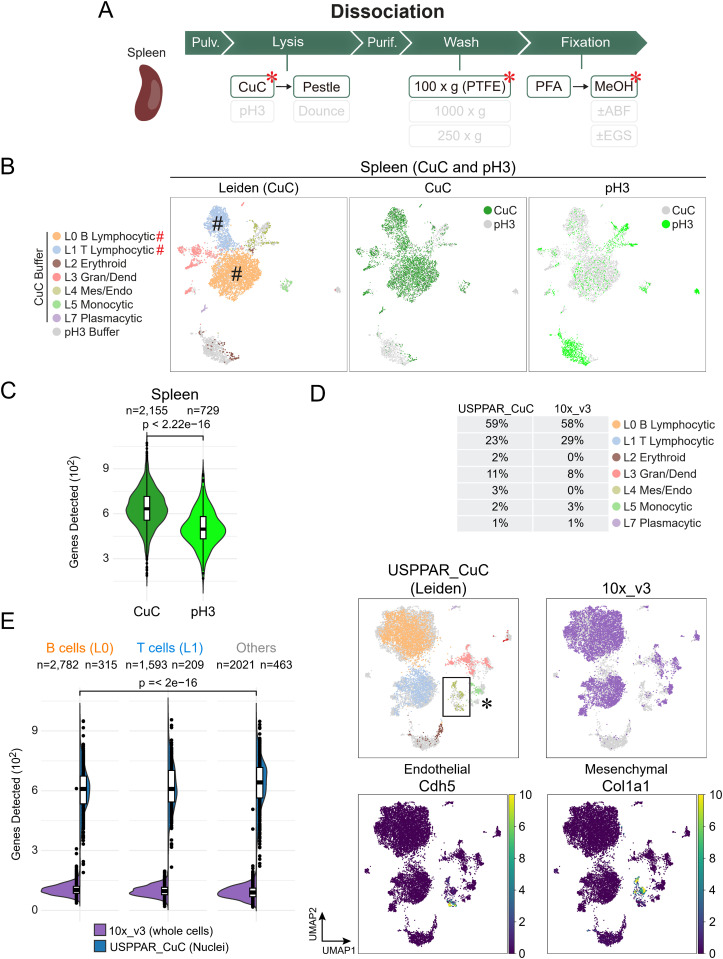
CuC lysis buffer provided higher gene detection sensitivity while enabling comprehensive cell type collection through one-pot lysis. **(A)** Overview of the nuclear extraction steps for one-pot nuclear collection from the spleen. Compared to the pH3 extraction system in S6B Fig, a CuC-containing lysis buffer was used, along with gentle washes on PTFE membranes (3 μm pore size) (S10A Fig) and additional methanol fixation (asterisks). **(B)** Integrated UMAPs of the 6,062 murine splenic nuclei (gene detected > 200) prepared using the pH3 (1,607 cells) and CuC (4,455 cells) buffer systems. The cells were colored according to Leiden labels and inferred cell types (left panel, Leiden) and the nuclear extraction strategies using either a pH3 buffer system (right panel, pH3, light green) or a CuC-based buffer system (middle panel, CuC, dark green). The ‘#’ symbols denote the lymphocytic clusters, which are significantly enriched with more cells using the improved system. **(C)** Violin and box plots showing the genes detected from 2,500 subsampled reads per barcode, prepared using the two nuclear extraction methods in **(B)**. **(D)** Integrated UMAPs of splenic datasets from one-pot lysis of the whole spleen using USPPAR (5,993 cells) and from sorted splenocytes using the commercial 10× platform (6,396). Barcodes with genes detected between 200 and 1,500 were used for modeling. Colors denote the Leiden clustering and inferred cell types of the 4,389 nuclei extracted using the CuC-based buffer and assayed with USPPAR (USPPAR_CuC), barcodes derived from splenocytes using the commercial platform (10×_v3, purple), and the normalized expression levels of endothelial (Cdh5) and mesenchymal (Col1a1) markers. The box encloses the mesenchymal/endothelial clusters significantly enriched with the one-pot lysis system. The asterisk denotes the region containing monocytic subclusters. The percentages of barcodes corresponding to each Leiden cluster in the USPPAR_CuC and 10×_v3 splenocyte datasets are shown above the respective UMAP plots. The color scale for gene expression ranges from dark purple (lowest normalized expression) to yellow (highest). **(E)** Violin and box plots show the genes detected from 2,500 subsampled reads per B cell (D, Leiden 0), T cell (D, Leiden 1), and other cell types (D, other Leiden groups) using the CuC nuclear extraction system with USPPAR (USPPAR_CuC) or from sorted splenocytes in the 10× platform reference database (10×_v3). In **(C)** and **(E)**, the horizontal bars denote the upper-quartile, median, and lower-quartile values descendingly in each box plot. *P*-values in **(C)** and **(E)** are obtained with the Wilcoxon rank-sum test. Mes: mesenchymal; Endo: endothelial; Gran: granulocytic; Dend: dendritic. Raw count data are available at GEO (GSE256014) and code for analysis and plotting at Zenodo (https://zenodo.org/records/13927875) for panels **(B)**-(E).

**Fig 7:**
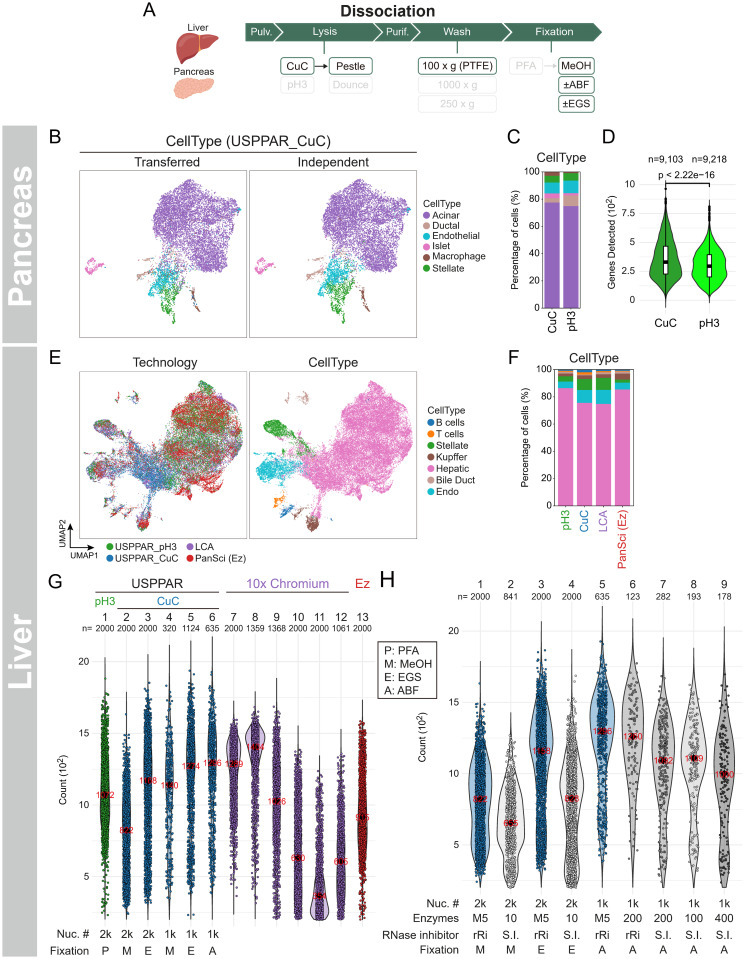
CuC-based one-pot lysis was also compatible with snRNA-seq of pancreas and liver. **(A)** Nuclear extraction steps for pancreas and liver using CuC lysis buffer. Unlike the spleen in Fig 6A, PFA fixation was omitted. For liver, methanol fixation was performed with EGS, ABF, or untreated (MeOH, methanol only). **(B)** UMAPs of 11,697 murine pancreatic nuclei (genes detected >200) prepared with CuC-based buffers. Nuclei are colored by cell-type annotations from independent modeling (Independent CellType) or transferred from the joint embedding (S11C Fig) of CuC and pH3 nuclei (Transferred CellType). **(C)** Stacked bar plots showing percentages of annotated non-blood cell types in nuclei prepared with CuC or pH3 lysis. Cell types were annotated using joint modeling of both CuC and pH3 datasets (S11C Fig, Joint). Barcodes corresponding to blood cells were identified based on their assignment to the immune category using a merged human pancreatic and immune reference dataset. **(D)** Violin and box plots showing the genes detected from 2,500 subsampled reads per barcode, prepared using the two nuclear extraction methods in Figs 7B (CuC) and S6E (pH3). **(E)** Joint UMAPs of 50,584 murine hepatic nuclei, including 12,646 nuclei from each technology: USPPAR_pH3 (pH3 lysis), USPPAR_CuC (CuC lysis), Liver Cell Atlas (LCA, 10× Chromium), and PanSci (EasySci platform). Nuclei are colored by technology (Technology) or by cell-type annotation from high-resolution Leiden clustering using the PanSci reference (CellType). **(F)** Stacked bar plots showing percentages of annotated cell types in **(E)**. **(G)** Violin plots with jittered points showing genes detected per nucleus across the four technologies in **(E)**. The LCA datasets are plotted separately due to the wide variation in median gene numbers across datasets. USPPAR_CuC and USPPAR_pH3: Nuc. # = nuclei per 20 µL RT reaction (1,000 = 1k; 2,000 = 2k). For USPPAR_CuC, Fixation = methanol alone (MeOH, M), methanol with ABF (A), or methanol with EGS (E). For USPPAR_pH3, Fixation = PFA (P). Only homebrew enzyme datasets are shown. **(H)** Violin plots with jittered points showing genes detected per nucleus using commercial or homebrew enzymes. Homebrew system (blue): homebrew enzymes were used for Barcoding and oligo phosphorylation. Commercial system (gray): adapters and RT primers were phosphorylated with commercial T4 PNK, RT was performed using Maxima H^−^ (10, 100, 200, or 400 U per 20 µL), SUPERase.In (S.I., 5 U per 20 µL) was included during RT, and T4 DNA ligase (40 U per 20 µL) was used for ligation. Lane 6: commercial enzymes were used throughout, except 5 ng/20 µL homebrew RNase inhibitor (rRi) replaced SUPERase.In during RT and pre-RT washes. Enzymes: M5, homebrew reverse transcriptase (5 ng/µL), or Maxima H^−^ at different U per 20 µL reaction. Nuc. #: nuclear number per 20 µL RT reaction (1,000 = 1k; 2,000 = 2k). Fixation: methanol alone (MeOH, M) or methanol with ABF (A) or EGS **(E)**. For (G) and **(H)**, barcodes were subsampled to 2000 per dataset if ≥2,000; smaller datasets were left unchanged. Medians are shown as black diamonds, with values indicated in red on each diamond. Statistical significance was assessed using the Wilcoxon rank-sum test with Bonferroni-corrected q values. For **(C)** and **(F)**, Chi-square tests with FDR-BH correction were used for statistical comparison of differences in cell-type proportions between groups. Raw count data are available at GEO (GSE256014) and code for analysis and plotting at Zenodo (https://zenodo.org/records/13927875) for panels **(B)**–**(H)**.

**Fig 8:**
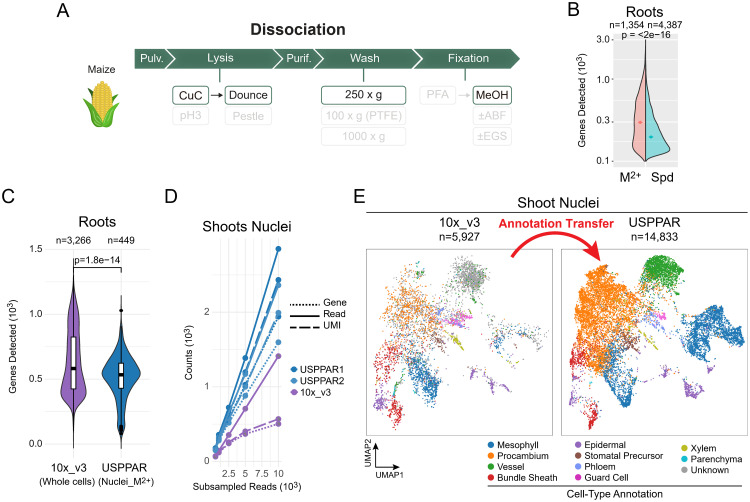
The nuclear extraction system was extendable to cell wall-bearing maize tissues. **(A)** Overview of the nuclear extraction steps for one-pot nuclear collection from maize tissues. Compared to the CuC extraction for mouse spleen shown in Fig 6A, washes on the PTFE membrane were replaced with centrifugation at 250*g* for 1 min to remove the abundant cell-wall debris from the nuclei. **(B)** Split-violin plot for genes detected per root nucleus when a spermidine (Spd)- or a divalent cation (M^2+^)-based buffer was used for lysis. The nuclei were prepared side by side and differentially barcoded at the RT step. Diamonds denote medians of each group. **(C)** Violin and box plots showing genes detected from 2,500 subsampled reads per root barcode, prepared using the M^2+^-based buffer (USPPAR), or from the protoplasts in the reference 10× dataset (SRR15686117−24) [26] (10×_v3). The horizontal bars in each box plot represent the upper quartile, median, and lower quartile values, listed from highest to lowest. *P*-values in panels **(B)** and **(C)** are obtained using the Wilcoxon rank-sum test. **(D)** The gene-detection efficiencies of maize shoot nuclei by snRNA-seq using USPPAR and 10× Chromium [27]. Median numbers of reads (solid), UMIs (dashed), and genes (dotted) per nucleus are plotted at down-sampled sequencing depths of 500, 1,000, 2,500, 5,000, and 10,000. Blue (USPPAR1) and light blue (USPPAR2) indicate USPPAR data from two independent Dissociation and Barcoding procedures, while purple represents the reference maize shoot dataset. With the reference dataset, only barcodes containing >500 genes were kept as in the original reference. **(E)** Integrated UMAPs of maize shoot nuclei prepared using USPPAR (USPPAR; 14,833 cells) and the 10× reference (10×_v3; 5,927 cells). The USPPAR and reference datasets were modeled together to infer low-dimensional embeddings and then plotted separately. The colors in the UMAP of the USPPAR dataset denote cell-type annotations transferred from the reference 10× dataset (with the exception of the ‘Unknown’ category from the 10×_v3 UMAP plot). Raw count data are available at GEO (GSE256014) and code for analysis and plotting at Zenodo (https://zenodo.org/records/13927875) for panels **(B)**–**(E)**.

In addition to T4 PNK and DNA ligase specified above, the homebrew Tn5 transposase used for tagmentation (Fig 1, Amplification stage, tagmentation) was prepared as described in the reference [31]. Reverse transcriptase (used in Fig 1, Barcoding stage, step 1) represents the major cost bottleneck, and RNase inhibitor is extensively required during the Barcoding stage and, potentially, the Dissociation stage in this and other platforms. Thus, we constructed and purified a thermostable reverse transcriptase, M5 [32]. M5 provided detection sensitivities for the mRNA of the housekeeping gene PSMB4 in human cell lysate comparable to those of commercial wild-type M-MuLV and RNase H-deficient SuperScript II reverse transcriptases based on RT-qPCR (S2C Fig, M5 versus MMLV and SSII, respectively). Using scRNA-seq on HEK293 cells, multiplexed across different RT conditions (S2 Table, SampleID 8), M5 was compared with the current best-in-class commercial RT, Maxima H Minus RT (Maxima H^−^). Fifty U of Maxima H^−^ per well yielded sensitivity comparable to homebrew M5 at 42°C (S2D Fig, lanes 6 versus 4, Wilcoxon *q* = 0.38). However, unlike Maxima H^−^, M5 RT tolerated an additional incubation at 50°C, resulting in enhanced sensitivity at this higher temperature compared to Maxima H^−^ (S2D Fig, lanes 8 versus 7, Wilcoxon *q* < 0.0001). Further, a purified oxidation-resistant rat ribonuclease inhibitor (rRi) [33] offered superior RNase inhibition compared to the commercial SUPERase·In (S.I.) (S2E Fig, lanes 3 and 4 versus 2), as evidenced by its ability to effectively block the degradation of a surrogate substrate that fluoresces upon RNase digestion. Consistent with the superior RNase inhibition observed above, rRi provided higher gene detection sensitivity than S.I. when comparing the 2 RNase inhibitors during RT in snRNA-seq of mouse liver tissue (Fig 7H, lanes 6 versus 7, 5 ng/μL rRi versus 0.25 U/μL S.I. [10]; median genes detected: 1,260 versus 1,092; Wilcoxon *q* < 0.0001).

Besides the recombinant proteins used in the Barcoding stage, we also expressed and purified TdT used in the Amplification stage (Fig 1, Amplification stage, Tailing). The TdT was capable of extending recessed-end DNA substrate under the same incubation conditions as the commercial one (S2F Fig, lane 3, homemade, versus lane 2, commercial). Furthermore, using maize roots and shoots as examples, both homebrew TdT [34] and the commercial version were capable of extending cDNA to yield scRNA libraries with similar sensitivities, as evidenced by the numbers of reads, UMIs, and genes detected upon serial subsampling of the same read numbers from each barcode (S2G Fig, root and shoot, respectively). Finally, the matched barcode distribution on the two-dimensional (2D) UMAP after batch information-free modeling (S2H Fig, cTdT versus hTdT) further demonstrated the effectiveness of the homebrew TdT in preparing libraries that accurately represent cell types, similar to those prepared with the commercial TdT.

Overall, the comparable efficiency and the simplicity of QC methods for assaying these enzymes make this platform generally accessible and special reagent-free, in addition to its inherent advantages of high cell capacity and independent of specialized equipment.

Two other parameters were evaluated and optimized using HEK293 cells. First, in the Dissociation stage, we used direct methanol fixation/permeabilization instead of the paraformaldehyde (PFA) fixation and Triton X-100 permeabilization commonly employed in methods like SPLiT-seq [10] and 10× Chromium. This approach was chosen to avoid RNA modification, simplify the procedure, and allow for long-term storage of cells in a liquid environment [35]. Indeed, methanol fixation alone was able to retain reads in 293FT cells (S2 Table, SampleID 2), while even an extra 15-minute aldehyde-dependent fixation with glyoxal [36] lowered the gene-detection efficiency (S2I Fig, +Glyoxal versus −Glyoxal). Thus, this simple and effective fixation method, involving the addition of 10× excess methanol to cell suspensions, was adopted in USPPAR for cultured cells. Next, we hypothesized that incorporating additional randomers during RT would enhance gene detection efficiency compared to using the polyA binding oligo(dT) primer (T16V) alone. To test this hypothesis, half of the cells underwent RT using barcoded T16V alone (−N9), while the other half were treated with T16V plus N9 (+N9) (S2 Table, SampleID 1). In this way, each library contained some cells barcoded with N9 and others without for a fair comparison. We found that cells barcoded with additional N9 exhibited higher numbers of detected UMIs (S2J Fig, +N9 versus −N9) and genes (S2K Fig, +N9 versus −N9) across all major RNA species. This increase was further supported by differential expression analysis, which revealed elevated levels of non-polyadenylated coding RNAs (e.g., histone mRNAs) [37] and noncoding RNAs (e.g., snoRNAs) (S2L Fig) in the +N9 group.

Given the improved sensitivity achieved by including N9, we further investigated whether traditional random hexamer (N6) priming or additional low-temperature incubation provided superior sensitivity (S2 Table, SampleID 8). Testing combinations of these parameters, we found that neither N6 priming (S2D Fig, lanes 1, 3) nor an additional 10-minute ambient temperature incubation (S2D Fig, lanes 1–2) significantly affected sensitivity compared to direct incubation at 42°C with N9 priming (S2D Fig, lane 4, all pairwise Wilcoxon *q* = 1). Thus, unless otherwise specified in S2 Table and the respective figure legends, RT reactions were performed using N9 priming and incubation at 42°C.

Collectively, the methanol-based fixative allows for simple, conjugation-free, and freeze-thaw-free single-step fixation, permeation, and storage of cellular samples, and inclusion of N9 during RT increases the number and diversity of genes detected. Finally, a cell strainer designed for minimal sample loss was fabricated to exclude cell and tissue clumps at the end of the Barcoding stage and after tissue pulverization during the Dissociation stage (S2M Fig).

### Engineering a trace-free TdT buffer system to completely tail unligated adapters and cDNA for single-tube preamplification (STA)

At the Amplification stage, cDNAs were tailed with TdT to add a poly(dA) primer-binding handle, followed by PCR preamplification (Fig. 1 and S1A, Amplification, steps 5–7). We hypothesized that the low-sensitivity issue in current high cell-throughput methods could be resolved by avoiding intermediate steps before cDNA amplification and by maximizing the efficiency of adding the second PCR primer binding site (Figs 1 and S1A, step 5). To avoid any purifications prior to cDNA purification, improved STA [14] was employed to avoid cDNA loss due to purifications before PCR amplification. To achieve this, the barcoded cells were lysed directly using sodium dodecyl sulfate (SDS)-free proteinase K, which was directly blocked with phenylmethylsulfonyl fluoride (PMSF) for purification-free continuation into the next steps. Next, we aimed to establish a system to maximize the efficiency of adding the second PCR primer binding site by DNA tailing. Complete DNA tailing is critical not only for adding PCR handles to cDNA but also for extending and inactivating any unligated adapters, which could serve as PCR primers and lead to incorrect barcode assignment. Because cDNA tailing based on template-switching was never complete using the original STA [14], we resorted to TdT-mediated polydeoxyadenylation followed by 2nd-strand synthesis as described in Quartz-Seq2 [5]. To develop an efficient tailing system for inaccessible DNA ends, we used both single-stranded (Fig 2A) or annealed double-stranded substrates (Figs 2B, 2D–2E, S3A, and S3B; arrows and the arrowhead indicating unextended substrates) as substrates to assess the tailing efficiency of TdT. In Quartz-Seq2, TdT relied on ThermoPol buffer, which contained the monovalent cation K^+^ that was detrimental to tailing (Fig 2A, lanes 3 and 4 versus 5 and 6). Besides, the enzymic cofactor in this buffer was Mg^2+^, which was known suboptimal for TdT, particularly with blunt or recessed-end substrates [15], such as many cDNA ends found on mRNA or in secondary structures. Consistent with the logic, a single-stranded substrate extended in ThermoPol or Mg^2+^-based buffer yielded shorter tails than that in a Co^2+^-containing buffer (Fig 2A, lanes 2 and 8 vs.7, respectively) and Co^2+^ could replace Mg^2+^ for tailing the ssDNA (Fig 2A, lanes 7 versus 8). Notably, the substitution of Co^2+^ for Mg^2+^ to rescue suboptimal tailing was even more prominent with a recessed-end substrate, as evidenced by the nearly complete upshift of the substrate when Mg^2+^ was replaced by Co^2+^ (Fig 2B, lanes 8 versus 4). Similar to 0.75 mM above, TdT with Co^2+^ achieved complete extension with similar efficiency at 0.25 mM (S3A Fig, lanes 3 and 5), whereas TdT with Mg^2+^ produced shorter extensions and more unextended product at both concentrations (S3A Fig, lanes 2 and 4). At 1.5 mM cofactor concentration, TdT with both cofactors Co^2+^ and Mg^2+^ showed comparable tailing lengths but left residual unextended substrate (S3A Fig, lanes 6 and 7). Furthermore, the inclusion of a low concentration (0.75 mM) of Mg^2+^ in the Co^2+^-containing reaction buffer led to a reduction in TdT extension efficiency, as evidenced by the increased residual unextended DNA duplex (Fig 2D, lanes 2 versus 3, arrows). Thus, using Co^2+^ alone at concentrations between 0.25 and 0.75 mM is optimal for the tailing system. A concentration of 0.75 mM was selected because it achieves efficient, complete tailing while minimizing potential interference from chelators such as Tris and EDTA present in the reaction mixture. In addition to the optimal buffer based on Co^2+^ and dATP, two other alternative ingredients, Mn^2+^ and dGTP, reported to improve tailing [15], were found either not to improve (S3B Fig, lanes 4 versus 3) or even to be detrimental (S3B Fig, lanes 5, 6 versus 3, 4). Besides, neither including dithiothreitol (DTT), a reducing agent (S3B Fig, lanes 7 versus 6), nor replacing Tris-HCl with HEPES, an alternative buffer (S3B Fig, lanes 1 versus 3), apparently enhanced the tailing.

In addition to the annealed DNA duplex with 3′-recessed ends as the substrate, we tested whether the optimized reaction system could also extend the previously intractable blunt and 3′-recessed ends in longer DNA fragments. To this end, the HindIII-digested PCR amplicon with both types of ends (see Fig 2C for the strategy to produce these end types) was extended using the same concentrations of dATP and TdT in different reaction formulations: the optimized system (Opti), the standard TdT buffer with Co^2+^ (TdT), and the ThermoPol buffer used for Quartz-seq2 (ThermoP). The optimized system offered a significantly superior extension at all four ends, whether blunt or 3′-recessed (Fig 2D, lanes 8 versus 9 and 10, arrowheads). This finding confirms the superior efficiency of the optimized tailing system compared to current methods for handling these challenging ends.

With the finalized formulation, five U of TdT efficiently tailed 2.5 pmole of the recessed-end substrate (Fig 2B, lane 4). However, the tailing efficiency dropped significantly either when the substrate concentrations increased (Fig 2E, lanes 1–4) or lysates containing 5,000-cell cDNA were included in the reaction (Fig 2B, lanes 2 versus 4). This reduced tailing efficiency due to substrate quantity could be addressed by increasing the TdT concentration, but not by increasing the cofactor Co^2+^ (Fig 2E, lanes 6 versus 8 and 7, respectively).

In conclusion, this TdT reaction system, which removes monovalent cations, replaces Mg²⁺ with Co²⁺, and utilizes high amounts of TdT, enabled the complete tailing of recessed-end DNAs—something that was previously considered impossible to our knowledge. Additionally, the simple reaction formulation permitted direct continuation into 2nd-strand synthesis by simply chelating residual Co^2+^.

### USPPAR showed high gene detection sensitivity that exceeded current high cell-throughput methods

These systemic optimizations were employed in USPPAR to collect single-cell transcriptomes from mixed human HEK293 and murine bone marrow MS5 cells with approximately 1,500 subsampled cells from a single preparation of 100,000 cells (S2 Table, SampleID 1). First, to validate barcode specificity and assess multiplet rates, we conducted a species-mixing analysis that revealed barcodes with clear species-specific enrichment in reads and minimal multiplets (Fig 3A). The low multiplet rates of 0% with 1,204 cells subsampled from a 100,000-cell input (Fig 3A, 3-round barcoding) and 0.2% with 16,450 from approximately 400,000 cells (S15A Fig, 4-round barcoding) were comparable to other high cell-throughput methods based on combinatorial barcoding, such as 1.3%–1.7% with 10,000–13,000 from standard 100,000 cells using the SPLiT-seq kit [38], 3.3% with 39,400 cells using the PIP-seq v4 kit, and lower compared to the expected 6.4% with 16,000 cells using the commercial 10× Chromium HT channels. Notably, this low multiplet rate will not increase with the number of subsampled libraries in the same experiment because each library is endowed with another layer of barcode during library amplification (S1A-S1B Fig, I_8_ from Index-2).

To further assess data quality and demonstrate platform competitiveness, the sequencing readout was further benchmarked against reference datasets from several state-of-the-art platforms, including 10× Chromium (HEK293T and NIH/3T3 cells), Quartz-Seq2 [5] (annotated HEK293 cells [39]), VASA-seq (HEK293T and mouse embryonic stem cells, mESCs) [4], sci-RNA-seq (HEK293T, HeLa S3, and NIH/3T3 cells) [40], PIP-seq (HEK293T, and NIH/3T3 cells) [9], and SPLiT-seq (HEK293, HeLa S3, and NIH/3T3 cells) [10]. The datasets modeled together showed 4 well-defined clusters of HEK293 cells (Fig 3B, Leiden, L0), HeLa S3 (Fig 3B, Leiden, L2), mouse embryonic stem cells (mESCs) (Fig 3B, Leiden, L3; Nanog, a pluripotency marker), and murine stromal cells (Fig 3B, Leiden, L1; Tagln, a stromal marker), reflecting the input cell types of respective platforms. In addition to the co-clustering of cell types in the 2D UMAP space, the pseudobulk reads from human cells were also segregated according to cell types (Fig 3C, PC1, squares versus circles). Beyond the distinctions by cell types, neighborhoods revealed technology-dependent differences (Fig 3C, PC2, colors): the correlations of outputs from sci-RNA-seq, SPLiT-seq, VASA-seq, and USPPAR were higher with each other. This was followed by those from 10× Chromium and Quartz-Seq2, and finally, that from PIP-seq (Figs 3C and S4A). The similarity of USPPAR to sci-RNA-seq, SPLiT-seq, and VASA-seq was reflected by their common higher intronic percentages (Fig 3D), a property that benefits the inference of cell-state transitions [41]. To demonstrate the informativeness of the intronic reads, we used murine liver as an example, as the two-cell type mixture of HEK293 and MS5 cells was insufficient to showcase cellular resolution. In the liver, the intronic counts alone could be independently modeled to yield 9 of the 10 clusters modeled using all genes (S7A Fig, Intron Only versus Exon + Intron). This indicates that the reads aligned to intronic regions were informative and derived from transcripts, not from untranscribed genomic DNA.

To evaluate detection sensitivity against these established platforms, standardized assessments were employed to benchmark the detection sensitivity across technologies [39]. The reads from HEK293 cells (Fig 3B; Leiden, L0) were subset from each platform to show UMIs and genes detected with the same read numbers per cell (S4B fig, UMIs; 3E and S4C, genes) and the cumulative numbers of detected genes from increasing randomly sampled cells (S4D Fig). These assessments showed that the detection sensitivity based on USPPAR matched or approached those based on sorting or microfluidics (10× Chromium, VASA-seq, and Quartz-Seq2) and exceeded those of the other high cell-throughput methods, SPLiT-seq, sci-RNA-seq, and PIP-seq, all based on combinatorial barcoding. Regarding dropout probabilities by expression magnitude [42], USPPAR also showed low dropout rates similar to special equipment-based methods (Fig 3F, curves), with a higher number of genes and UMIs detected per 50 cells compared to the other three methods based on combinatorial barcoding (Fig 3F, 13,271 genes with USPPAR versus 11,937, 11,302, and 10,433 with sci-RNA-seq, SPLiT-seq, and PIP-seq, respectively).

Collectively, USPPAR offered a higher gene detection efficiency and a lower dropout rate compared to the other current high cell-throughput methods. It provided a minimum capacity of 400,000 cells in a single experiment (S2 Table) while offering a low multiplet rate, surpassing those of other high-sensitivity methods that depend on special equipment. Additionally, the output accuracy of this system was demonstrated by the strong correlation in gene expression results between this system and other established methodologies.

### USPPAR profiled PBMCs with enhanced sensitivity through lysine conjugation during methanol fixation

In contrast to dissociated cells from cell lines, Profiling scRNA-seq in primary PBMCs is more challenging due to the substantially lower number of detectable transcripts per cell. This challenge is also demonstrated by sci-RNA-seq3’s inability to collect sufficient reads for analysis of primary PBMCs [19], despite its effectiveness with the cell lines described above. The challenge makes PBMCs an ideal target for benchmarking the performance of USPPAR against other technologies. Furthermore, their complex cellular composition provides an opportunity to showcase the cellular resolution achievable with USPPAR. Initially, PBMCs were isolated using the standard density gradient procedure, and methanol fixation was then applied, as previously done for HEK293 cells (S2 Table, SampleID 9). Based on the medians of genes detected per PBMC at sequencing depths of 2,500–10,000 reads per cell, the sensitivity of USPPAR remained higher than those of SPLiT-seq (Evercode v3) and PIP-seq V [43] (Fig 4A, Genes, USPPAR_MeOH versus SPLiT-seq and PIP-seq), consistent with results obtained using HEK293 cells (Fig 3). However, the sensitivity of USPPAR was lower than that of the GEMX-v4 Chromium platform (Fig 4A, Genes, USPPAR_MeOH versus 10× Chromium), contrasting with the comparable sensitivities observed between the two technologies using HEK293 samples (S4C Fig).

We reasoned that residual RNases, either from plasma or released from PBMCs, caused ongoing RNA degradation during the RT reaction in sci-RNA-seq3 and USPPAR, leading to detection failure or reduced sensitivity. In contrast, PFA fixation in SPLiT-seq, which also uses combinatorial multiplexing with thousands of cells per RT reaction, may protect RNA from degradation by immobilizing or inactivating RNases. Consistent with this reasoning, incubating RNase A with PFA for 30 min reduced its RNA-hydrolyzing activity compared with the untreated control (Fig 4B, lanes 5 versus 6). Because PFA fixation can modify RNA and requires a narrow working concentration range [12], we tested an NHS ester–based crosslinker, ethylene glycol bis(succinimidyl succinate) (EGS), which does not modify RNA. Similar to PFA, EGS quenched RNase A’s hydrolyzing activity under identical incubation conditions (Fig 4B, lane 1 versus 6). Surprisingly, 4-azidobenzoyl fluoride (ABF), another lysine-targeting conjugation reagent lacking crosslinking ability, also inactivated RNase A [44] (Fig 4B, lanes 3 versus 6). The fact that both modifiers inactivated RNase A, regardless of their crosslinking ability, indicates that lysine modification alone is sufficient for inactivation. Finally, although both modifiers inactivated RNase A after 30 min, they did not protect RNA when added without preincubating RNase A with the modifiers (Fig 4B, lanes 2 and 4 versus 1 and 3).

We used ABF here because, unlike EGS, its lack of crosslinking may improve enzyme and oligo penetration and thereby enhance detection sensitivity. The addition of ABF during methanol fixation increased the medians of UMIs and genes detected across all subsampled read depths compared with PBMCs fixed with methanol alone (Fig 4A, UMIs and Genes, USPPAR_ABF versus USPPAR_MeOH). This improvement enabled USPPAR to achieve a gene-detection sensitivity comparable to that of the GEMX-v4 Chromium platform, measured by the median numbers of genes detected per cell at commonly used sequencing depths of 2,500–10,000 reads per cell (Fig 4A, Genes, USPPAR_ABF versus 10× Chromium), and was surpassed only by the low-throughput Quartz-seq2 (Fig 4A, Genes, Quartz-Seq2). The PBMC dataset generated by USPPAR integrated well with datasets from the other four technologies, with USPPAR-derived cells represented across all major and minor clusters (Fig 4C, Technology). Despite originating from different biological samples, the cell type distribution in USPPAR from the joint model (Fig 4C, CellType) closely resembled that of PIP-seq, followed by SPLiT-seq, 10× Chromium, and Quartz-Seq2 (Fig 4D, stacked bar plots). Moreover, cell type annotations from the joint model (Fig 4E, Transferred) were largely concordant with those obtained from the independently modeled USPPAR dataset (Fig 4E, Independent). Even a small group of dendritic cells in the USPPAR dataset could be correctly annotated (Fig 4E, Independent, Dendritic cells), and major cell types, including CD4⁺ and CD8 ⁺ T cells and B cells, formed well-defined subclusters (Fig 4E, CD4 T, CD8 T, and B cells), demonstrating USPPAR’s ability to achieve high-resolution cellular mapping. In addition to cell-type annotation, each cluster expressed its canonical marker genes (S5A and S5B Fig), supporting the robustness of USPPAR for PBMC scRNA-seq.

To ensure that ABF treatment does not alter cellular composition, we compared PBMCs fixed with methanol alone versus those treated with ABF. Compared with cells fixed with methanol alone, ABF-treated cells integrated well with MeOH-treated cells (S5C Fig, MeOH versus ABF) and comparable cell-type percentages (S5D Fig, MeOH versus ABF), indicating that ABF improves gene detection sensitivity without distorting gene expression profiles or altering cell-type distributions.

Beyond cultured cells, benchmarking with PBMCs demonstrates that USPPAR can profile cells with substantially lower transcript counts while preserving sensitivity and cell-type resolution, with performance further enhanced by adding a lysine-conjugation agent during methanol fixation.

### A pH 3 nuclear extraction system enabled one-pot lysis of liver and nuclease-rich pancreas tissues for single-nucleus RNA-seq (snRNA-seq)

In addition to cultured and primary cells in singlets, collecting the single-cell transcriptomes from organs currently requires variable tissue-specific procedures. These different procedures incur unavoidable batch artifacts and may potentially alter cell status during processing, which biases the comparison of the same cell types from different tissues. To broaden the application of our system beyond cultured cells and enable its streamlined and direct use on tissues, our next objective was to minimize the duration of cell exposure to unphysiological environments and avoid tissue-specific dissociation requirements [18]. Here, we opted to collect nuclei directly by pulverizing tissues at liquid-nitrogen temperature (S6A Fig for making a liquid-nitrogen-tolerant polyimide roll for pulverization), followed by immediate mechanical lysis and filtration through a custom strainer (S2M Fig) to remove large clumps with minimal sample loss. This strategy supposedly released nuclei from all tissue types regardless of extracellular matrices and rapidly exposed nuclei to lysis buffers upon thaw. Based on this approach, the Dissociation stage can theoretically be divided into five distinct steps (Figs 1A, Dissociation; S6B, example stepwise chart): first, the initial pulverization of frozen tissue (Pulverization); second, lysing the tissue fragments using lysis buffers with mechanical disruption, followed by filtration through strainers (Lysis); third, density-gradient centrifugation to purify high-density nuclei from low-density cytosolic components (Purification); fourth, washing to remove density-gradient reagents (Wash); and finally, fixation of the purified nuclei using crosslinking agents (e.g., PFA) and/or non-crosslinking agents (e.g., methanol) (Fixation).

Regarding the Lysis step, a major caveat is the widespread presence of nucleases in blood and some tissues that rapidly degrade nuclear RNAs upon tissue lysis. This challenge might explain why a universal tissue lysis system for snRNA-seq has not yet been developed. As an initial example to identify a unified workflow with broad specimen compatibility in USPPAR, the murine pancreas was chosen because it contained abundant nucleases [25]. Initially, we adopted a pH 3 (pH3) buffer-lysis system [25] based on two key findings: First, the increased release of quenched fluorophore in the presence of sucrose alone (S6C Fig, lanes 1 versus 2), and an even greater increase with pancreatic lysate (S6C Fig, lanes 8 versus 2), indicated the widespread presence of nucleases. Second, these nuclease activities were effectively blocked in a pH-dependent manner (S6C Fig, lanes 3–6 versus 1; lanes 9–12 versus 7).

Using the pH3 lysis buffer, the pancreas was pulverized and lysed, with the strained nuclei further purified using a density gradient before fixation with PFA (S6B Fig, Pulverization to Fixation;S6D Fig, pH3, Pancreas: lysed pancreatic nuclei). These nuclei then underwent the same Barcoding and Amplification stages as the dissociated cells. The transcriptome collection from pancreatic nuclei proved successful (S2 Table, SampleID 3), as evidenced by distinct cell type clustering in the 2D UMAP space (S6E Fig). Various pancreatic cell populations were clearly identified, including ductular cells (marked by Krt19+/Epcam+), acinar cells (Pnlip+/Try5+), and islet cells (Pax6+). Additionally, blood, vascular, and mesenchymal cells were well-represented, with all cell types displaying their corresponding marker expressions (S6F and S6G Fig). Besides, the identical procedure also unveiled hepatocytic subclusters with various gene expression patterns (S2 Table, SampleID 4), as well as Krt19 + bile duct, Reln+ stellate, and Adgre1 + Kupffer cells [45] (S7A–S7C Fig). In summary, the pH3 lysis buffer was effective for the Dissociation stage, successfully releasing RNA-containing nuclei from both the liver and the more challenging, nuclease-laden pancreas.

### The pH3 extraction system was suboptimal for the unbiased collection of all cell types in another challenging organ, the spleen

Next, we tested the pH3 lysis system on the spleen, another nuclease-rich tissue that is refractory to one-pot lysis for snRNA-seq. However, the identical acidic system yielded significantly fewer high-quality splenic barcodes (1,607 splenic cells with more than 200 genes detected per cell) compared to hepatic barcodes (17,487 hepatic cells) when equal numbers of hepatic and splenic nuclei were processed simultaneously (S2 Table, SampleID 4; S6D Fig, pH3, Liver and Spleen: lysed nuclei). This data was obtained using a 36,000-cell aliquot from a preparation of 400,000 cells (S8A Fig; color: liver, black: spleen). This dramatic loss stemmed from the underrepresentation of Cd19+ B and CD3e+ T cells (15.3% and 4.9% of all splenic cells, respectively, using the pH3 buffer) (S8B Fig; Leiden, denoted by #; Cd19; and Cd3e), which are major cell populations in the spleen [45]. Beyond reduced cell percentage, comparison of white blood cells (Ptprc⁺ Leiden clusters) between spleen and liver revealed lower gene detection efficiency in splenic nuclei. This difference was evident in the reduced number of genes detected per barcode in the splenic sample compared to the hepatic one (S8C Fig). Since the two organs were lysed and barcoded concurrently, the reduced sensitivity is likely due to mRNA loss or degradation during the lysis of the spleen. This aligns with the fact that the spleen contains abundant nucleases, and as a result, no datasets have yet been derived from one-pot nuclear extraction of the spleen.

Besides the underrepresentation of lymphocytes, another issue was that this imbalance in barcode numbers between the spleen and liver also resulted in an aberrant Leiden cluster, Leiden 6, with hepatocyte identity appearing in the spleen dataset (S8B Fig; Leiden, asterisk; Alb). The barcodes in Leiden cluster 6 were almost exclusively found in the nuclei derived from the pH3 lysis system (82 of 83 cells in Leiden 6) and not in those from the other system we developed later (S8B Fig, Leiden, gray). The reads contributing to the Leiden-6 barcodes were found to have an 8-bp truncation (S8D Fig, All versus Leiden 6, pink box). Accordingly, the frameshift could be corrected to reveal the contributing cells in the combined liver–spleen dataset of the same experiment (S8E Fig, red: before barcode correction, sky-blue: after correction). The 7,370 corrected barcodes showed a cell-type distribution identical to that of all 19,097 cells in this dataset (S8F Fig). Furthermore, among the 7,370 cells, 6,097 are hepatocytes (S8F Fig, hepatic), which explains the enrichment of hepatocytic markers in the Leiden-6 cluster. This truncation likely occurred during PCR of library preparation or colonial amplification, yet only accounted for a minority of reads (96,165,705 reads assigned to cells in this experiment, 95,504,092 with the expected frame, and 70,242 with the 8-bp truncation, representing 83 Leiden6 cells among all 19,094 barcodes). This leakiness by misassignment appeared because only a few nuclei from the spleen passed the quality check, compared with many more (~10×) liver cells using the pH3 lysis system. Regardless, the issue could be addressed by simply gating the non-truncated reads at specific windows in the barcode-generating reads.

### *Cu* ^*2+*^ *-chelator complexes effectively inhibited a variety of nucleases across different pH levels without causing undesirable precipitation*

We hypothesized that lymphocyte loss was likely caused by the pH3 lysis buffer and PFA fixation, which failed to retain nuclear RNA in small lymphocytic nuclei during Barcoding incubation and washes. Although methanol was effective for fixing dissociated cultured cells, it was incompatible with nuclei extracted using the pH3 lysis buffer, as it led to extensive clumping. We therefore sought a new nuclear extraction buffer that could immediately quench endogenous nucleases in tissues while remaining compatible with methanol fixation. Regarding the inhibition of nuclease activity, since the histidine residues are critical for the RNA catalysis of RNase A [46], the most common and well-studied form of RNases. The RNase is readily inactivated by diethyl pyrocarbonate (DEPC) [47] that covalently modifies histidine residues [48]. Although DEPC has been used to collect nuclei from embryos for snRNA-seq [49], we found DEPC was incompatible with our nuclear extraction system. Specifically, when pancreatic tissue was lysed in a buffer containing DEPC (1%) and subjected to density-gradient purification, the nuclei accumulated at the interface between the tissue lysate and the 30% iodixanol lysis buffer (S9A Fig, DEPC, asterisk), where they aggregated into large clumps (S9B Fig, DEPC). This resulted in most nuclei remaining above the 30% iodixanol layer, with barely any nuclei descending to the 30%−60% interface (S9A Fig, DEPC, arrow) to be free of cytoplasmic and extracellular contaminants.

Thus, beyond the pH3 buffer and DEPC, we resorted to other agents that target histidine residues for RNase inhibition as well. Histidine residues have specific and strong interactions with transition metal ions, such as Co^2+^, Ni^2+^, Zn^2+,^ and Cu^2+^ [50]. We hypothesized that this RNase could also be inhibited by using cations to bind to the imidazole side chain of the active-site histidine residues, thereby preventing them from accessing the substrate RNAs. Based on this logic, our initial tests using the two strongest binders, Zn^2+^ and Cu^2+^ [51], revealed that Cu^2+^ effectively diminished RNase activity in pancreatic lysate in a concentration-dependent manner(S9C Fig, lanes 4–6). However, the hydroxide form of Cu^2+^ (Cu(OH)_2_) is minimally soluble in aqueous solution at neutral pH, leading to precipitation even at concentrations as low as 1 mM (Fig 5A, lanes 4–6, triangle). Besides, Cu^2+^ led to severe protein aggregation at pH above 5.5, as evidenced by the co-incubation of this ion with bovine serum albumin (BSA), which caused precipitation both visually and as indicated by OD600 readings (Fig 5A, lanes 3–6, square). This aggregation is likely due to increased deprotonation of histidine residues, which enhances their interaction with Cu^2+^. This consequential cross-linking of histidine residues on different proteins prevents the clean-background isolation of nuclei. Theoretically, this crosslinking property could be blocked by chelators that occupy some coordination sites of Cu^2+^. Indeed, precomplexing Cu^2+^ with citric acid, a chelator, resolved this issue at all pH values tested in the presence of BSA (Fig 5A, circle). Importantly, the chelated Cu^2+^ was still capable of blocking pancreatic nuclease activity in a dose-dependent manner, similar to the unchelated form (Fig 5B, circles versus squares). Moreover, this nuclease-blocking activity was dependent on Cu^2+^ and not on citric acid alone (Fig 5C, pH 4.5 and 7.2, lanes 5 versus 3 and 4). Further, the Cu^2+^-citrate complex (CuC) was active at both acidic and neutral pH levels (Fig 5C, pH 4.5 and 7.2, lanes 4 versus 2) and was capable of blocking the activities of other nonselective DNA/RNA endonucleases, such as micrococcal nuclease and benzonase, in addition to RNase A (Fig 5D, lanes 4, 6, 8 versus 3, 5, 7), indicating its nonselective inhibition against different nuclease types.

To test whether nuclease inhibition extends beyond the CuC and how chelator valency affects potency, we examined additional Cu² ⁺ –chelator combinations. First, Cu²⁺, along with another chelator, nitrilotriacetic acid (NTA), also inhibited nuclease activity (S9D Fig, lane 5; 5C, pH 4.5 and 7.2, lane 3). However, the presence of Cu²⁺ with ethylenediaminetetraacetic acid (EDTA) was ineffective in inhibiting nuclease activity (S9D Fig, lane 6). This differential partner requirement for Cu² ⁺ highlights that the availability of free coordination sites (2/6 with quasidendate NTA, 3/6 with tridendate citric acid, and 0/6 with hexadentate EDTA) is crucial for achieving nuclease-blocking activity in CuC, rather than the type of chelator itself. At the concentration of 10 mM (S9E Fig, lane 3), this complex offered comparable RNase inhibition to pH3 citric acid (S9E Fig, lane 5), approaching the inhibition by DEPC (S9E Fig, lane 4), the RNase inhibitor [49] that extensively modifies amino acids [52] and nucleic acids [53] covalently. Additionally, compared with the immediate inclusion of the CuC (S9E Fig, lane 6), the delayed addition of the CuC to the reaction showed minimal quenching of fluorescence (S9E Fig, lanes 7 versus 2). This further supports the idea that the complex reduces fluorescence by preventing the cleavage of DNA/RNA that links the fluorophore to the quencher, rather than by directly quenching the fluorophore itself. Finally, in contrast to the tissue lysed with the buffer containing DEPC, the same lysis buffer with CuC instead of DEPC kept nuclei single (S9B Fig, CuC) and was compatible with density-gradient purification, forming a nuclei layer at the 30%–60% interface (S9A Fig, CuC, arrow).

Besides the in vitro reporter cleavage assays above, to mimic nuclear extraction by direct lysis of nuclease-rich organs, HEK293 cells were lysed in the presence of pancreatic lysate, with or without the CuC. The extracted nuclei were assayed for the mRNA levels of two housekeeping genes, GAPDH and PSMB4. The CuC was necessary to protect and retain nuclear RNA from complete degradation by pancreatic nuclease at both pH 4.5 and 7.2, when compared to nuclear samples prepared without this complex (Fig 5E, CuC, 10 versus 0).

In addition to the mock assay mixing HEK293 cells with pancreatic lysate, we directly assessed RNA integrity in nuclei isolated from liver, pancreas, and spleen using the two lysis systems (pH3 and CuC). RNA quality was measured by the percentage of fragments larger than 200 bp (DV200), providing a more comprehensive evaluation of RNA preservation across conditions. The presence of RNase activity in all three organs, particularly the pancreas and spleen, was evident by comparing residual RNA fragments in nuclei extracts using the pH3 lysis buffer and a lysis buffer without CuC supplementation (S9F Fig, pH3 versus None). Adding CuC to the lysis buffer during the extraction process resulted in reduced RNA degradation in all three organs, as evidenced by a better-preserved 28S peak in the liver and spleen (S9F Fig, Liver and Spleen, 28S, CuC versus None) and higher DV200 values in the pancreas (S9F Fig, CuC, pancreas, DV200, CuC: 90.3% versus None: 80.5%).

Collectively, CuC proved effective during nuclear extraction due to its potency in inhibiting various nucleases, its activity across a wide pH range, and its ability to protect RNAs in lysed nuclei, all while avoiding protein precipitation.

### The optimized CuC system enabled USPPAR to capture comprehensive cell states from the spleen in a single pot

Besides including CuC during lysis, two additional strategies were employed to enhance the integrity of lymphocytic nuclei (Fig 6A, asterisks). First, unlike nuclei extracted using the pH3 system, those extracted with the new lysis buffer allowed for clump-free fixation in methanol, similar to intact whole cells. Additional methanol fixation would be advantageous for ensuring that the extracted nuclei withstand multiple rounds of incubation and washes during the RT and barcoding steps (Fig 6A, Fixation). Second, we designed a cell retention system consisting of a 3-μm pore size hydrophilic polytetrafluoroethylene (PTFE) membrane sandwiched between an open cap and a bottom-free bottle (S10A Fig). This screw-cap filter is leak-proof, offers approximately twice the surface area of conventional spin columns, minimizes nuclear adsorption to angled plastic tube walls during spin-down, reduces the centrifugal force from 1,000*g* to 100*g* during washes to minimize nuclear damage before fixation (Fig 6A, Wash), and is compatible with future centrifugation-free automation systems.

Given the combination of lysis buffer containing the CuC, dual fixation, and gentle cell washes on a filter membrane, mouse spleens, an RNase-rich, bloody organ [16], were lysed in one pot and processed for snRNA-seq (S2 Table, SampleID 5; S6D Fig. CuC, Spleen: lysed nuclei). The snRNA-seq approach rescued the loss of lymphocytic barcodes when using our original pH3 lysis buffer system (Figs 6B, Leiden; CuC versus pH3; S10B and S10C, representative marker expression [23,54]). Additionally, standardizing the comparison by using the same number of subsampled reads (2,500) per barcode in both datasets revealed an increased number of genes detected per barcode with the optimized system (Fig 6C, CuC versus pH3), indicating its superior sensitivity. Further, a dataset from purified splenocytes obtained using the 10× commercial platform (SampleID: 10k_Mouse_Splenocytes_5p_gemx, published on April 16, 2024) was used for additional benchmarking. Our snRNA-seq results integrated well with the commercial dataset, revealing blood cell types with proportions comparable to those identified by the commercial 10× platform, which required selective enrichment of splenocytes (Fig 6D, USPPAR_CuC versus 10×_v3). Among the blood cells, only a subpopulation of monocytic cells was selectively present in the 10× dataset, but not in the CuC dataset (Figs 6D, asterisk; S10D, Monocyte A). To reveal the identity of this cluster, genes differentially expressed between the two sibling monocyte clusters were used to identify biological process Gene Ontology (GO) terms specific to each cluster. Only the A population showed significant enrichment of GO terms (*q* < 0.05), while the B subset did not. These enrichment terms were mostly about the activated antigen-presenting signatures (S10E Fig). The absence of these activated monocytic cells in our dataset was likely due to the relatively immature immune system of the young donor mice, which were less than 3 weeks old. Notably, our one-pot nuclear extraction revealed a more than 10-fold increase in cells expressing mesenchyme- or endothelium-specific markers, Col1a1 and Cdh5, respectively. (Fig 6D, boxed). To standardize gene-detection sensitivity, barcodes for B cells, T cells, and other cell types (S10F Fig, B, T, and Others) in the USPPAR and 10× datasets were subsampled to the same number of reads. Our system detected more genes than the 10× platform across all these cell types (Fig 6E; USPPAR_CuC versus 10×_v3). Overall, the comparable percentages of blood cell types, increased coverage of mesenchymal cells, and higher gene detection sensitivity compared to the 10× platform demonstrate that our one-pot lysis system effectively and conveniently handles the challenging spleen.

### CuC-based one-pot lysis was also compatible with snRNA-seq of pancreas and liver and enabled integration across platforms, lysis buffers, and organs

We next tested whether CuC lysis buffer was effective for nuclear extraction from the pancreas, a highly RNase-rich tissue representing a challenging model, and from the liver, which has lower RNase levels and is widely used for snRNA studies. The nuclei were extracted using the same procedure as for spleen, except without PFA fixation, to provide additional confirmation of the efficiency of lysine-conjugating agents as previously tested with PBMCs (Fig 7A).

As with the pancreas (S2 Table, SampleID 10; S6D Fig, CuC, Pancreas: lysed nuclei), the validity of the USPPAR_CuC data is supported by several lines of evidence. First, independent modeling and cell type assignment of snRNA-seq data revealed clusters corresponding to all major pancreatic cell types, including acinar, ductal, endothelial, mesenchymal, islet, and macrophage (Fig 7B, CellType, Independent), which matched well with the cell type assignments transferred from joint modeling of pH3 and CuC data (Fig 7B, CellType, Transferred from S11C Fig, Joint). Second, each cluster expressed hallmark markers characteristic of its respective cell type, further confirming the annotations (S11A and S11B Fig, representative marker expression). Third, cross-species cell type assignment, typically a challenging task, was successfully achieved using the human pH3 10× Chromium reference dataset [25], further supporting the validity and robustness of the USPPAR_CuC dataset. Finally, even when USPPAR_CuC and USPPAR_pH3 data were independently modeled and annotated, cells of the same type from both lysis systems co-clustered in the joint embedding space (S11C Fig, Joint versus CuC and pH3).

When comparing cell type compositions between USPPAR_CuC and USPPAR_pH3, we observed that the USPPAR_pH3 dataset contained abundant hematopoietic cells, likely due to blood carryover, as indicated by a prominent cluster of red blood cells and reticulocytes that are not typically abundant in the pancreas (S6F and S6G Fig). Because both USPPAR_CuC and USPPAR_pH3 datasets were annotated in an unsupervised manner using a merged reference of human pancreatic [25] and immune [55] datasets, non-blood parenchymal and stromal cells were defined and retained as those assigned by the pancreatic reference (and not the immune reference) (Fig 7C). Although the samples were not identical and may reflect biological variation, the cell-type composition of USPPAR_CuC showed a higher proportion of islet cells and macrophages, whereas USPPAR_pH3 exhibited a higher proportion of ductal cells (Fig 7C, chi-squared test with FDR-BH *q* < 0.05). Finally, we compared gene detection sensitivity in USPPAR_CuC and USPPAR_pH3 at a read depth of 2,500 reads per cell. Sensitivity was modestly higher in USPPAR_CuC, even when nuclei were fixed with methanol only, without crosslinkers or lysine-targeting modifiers (Fig 7D, CuC versus pH3).

No snRNA-seq datasets of mouse pancreas or spleen were available for a fair comparison, likely because of their high RNase content (S9F Fig). Consequently, liver datasets were used for benchmarking (S2 Table, SampleID 11 and 12; S6D Fig, CuC, Liver: lysed nuclei). Among the four technologies included in the PBMC benchmark, raw sequencing data were unavailable for SPLiT-seq and PIP-seq; therefore, we used datasets from the Liver Cell Atlas (LCA, 10× Chromium v2 and v3) and PanSci (based on EasySci, successor to sci-RNA-seq3) for benchmarking. Again, USPPAR datasets from CuC (USPPAR_CuC) and pH3 (USPPAR_pH3) lysis integrated well with those from the other two methods in a joint embedding space (Fig 7E, Technology), showing largely comparable cell-type compositions across technologies and lysis strategies (Fig 7F). Despite using different biological samples, the CuC dataset was enriched for non-parenchymal cells, including stellate, endothelial, B, and T cells (chi-squared test with FDR-BH *q* < 0.05), resembling LCA, whereas pH3 was enriched for parenchymal cells (hepatocytes) at the expense of non-parenchymal populations, resembling PanSci (chi-squared test with FDR-BH *q* < 0.05). Furthermore, cell type annotations transferred from the joint model (S12A Fig, Transferred from 7E) closely matched those from independent modeling of the USPPAR_CuC data (S12A Fig, Independent). These annotations were validated by the expression of canonical markers in each cluster (S12B and S12C, representative marker expression). The accurate cell-type assignments and expected expression of hallmark genes in the CuC dataset alone support its independent information content.

Further, to assess detection sensitivity, we calculated the number of genes detected per nucleus (from 5,000 subsampled reads) for each platform. The median gene count in USPPAR_pH3 (Fig 7G, lane 1; median 1,072) exceeded that of PanSci (Fig 7G, lane 13; median 915) and four of six 10× Chromium datasets (Fig 7G, lanes 9–12; medians 354–1,026), but was lower than the remaining two 10× Chromium datasets (Fig 7G, lanes 7–8; medians 1,289 and 1,404) (all Wilcoxon q < 0.001). With USPPAR_CuC samples prepared without any crosslinking or covalent conjugation agents (PFA, EGS, or ABF), the median gene count per nucleus depended on the number of nuclei used per reaction: processing 2,000 nuclei per 20 µL RT and ligation reactions yielded a median of 822 genes per nucleus, whereas 1,000 nuclei increased the median to 1,140 genes (Fig 7G, lanes 2 versus 4; Wilcoxon *q* < 0.001). With 1,000 methanol-fixed liver nuclei per reaction (no conjugation or crosslinking), median gene counts were similar between USPPAR_CuC (1,140) and USPPAR_pH3 (1,072) (Fig 7G, lanes 4 versus 1; Wilcoxon *q* = 1). Additionally, the inclusion of EGS (Fig 7G, lanes 3 and 5 versus 2 and 4; medians 1,168, 1,274, 822, and 1,140, respectively) or ABF (Fig 7G, lanes 6 versus 4; medians 1,296 and 1,140) enhanced gene detection sensitivity in the CuC dataset compared with methanol-only samples (all Wilcoxon *q* < 0.001). This effect is consistent with the RNA-protection role of these agents and their improvement of sensitivity in the PBMC dataset (Fig 4A and 4B). Notably, the improvement was more pronounced in the 2,000-cell dataset, as reflected by the larger difference in medians (346 genes) compared with the 1,000-cell dataset (EGS: 134 genes; ABF: 156 genes). The sensitivity of USPPAR_CuC (1,000 cells) with EGS or ABF was statistically indistinguishable from the second-highest quality output with 10× Chromium (Fig 7G, lanes 5 and 6 versus 7; medians 1,274, 1,296 versus 1,289; Wilcoxon *q* = 1 and 0.5, respectively).

Beyond gene detection sensitivity, we examined nuclei fixed with MeOH alone or with ABF and EGS to assess their effect on cell-type composition. All three treatments, methanol fixation alone and methanol combined with EGS or ABF, revealed all major liver cell populations in 2D UMAP space (S12D Fig). Notably, minor compositional differences were observed among the datasets generated under the three fixation conditions: the EGS dataset showed increased blood cells (Kupffer, B, and T) and reduced stellate cells; the ABF dataset enriched stellate cells while lowering Kupffer cells; and the methanol-only dataset showed reduced B and T cells (S12E Fig; chi-squared test with FDR-BH *q* < 0.05).

Instead of homebrew enzymes, we used liver samples to test USPPAR compatibility with commercial enzymes: T4 PNK to phosphorylate oligos for RT and ligation, T4 DNA ligase for ligation-mediated barcoding, Maxima H^−^ for RT, and S.I. as an RNase inhibitor throughout the Barcoding stage. First, to identify T4 PNK and DNA ligase amounts sufficient for complete phosphorylation and ligation of a substrate oligo, we designed a looped-oligo-based assay. (S12F Fig). Using this assay, we determined the optimal amounts of T4 PNK and DNA ligase (S12G Fig, lane 4). Next, liver samples lysed with CuC buffer (Fig 7A) were processed through Barcoding and Amplification stages using either homebrew or commercial enzymes in parallel. With a relatively low amount of Maxima H^−^ (10 U) and a comparatively large number of liver nuclei (2,000) per 20 µL RT reaction, sensitivity was lower than with nuclei barcoded using homebrew enzymes, regardless of EGS treatment (Fig 7H, lanes 2 and 4 versus 1 and 3; medians 655, 828, 822, 1,158; Wilcoxon *q* < 0.0001). This is consistent with reduced gene detection when 10 U of Maxima H^−^ per 20 µL were used for 1,000 HEK293T cells, compared with 50 U, which yielded higher sensitivity (S2D Fig, lanes 5 versus 6; Wilcoxon *q* < 0.0001). However, even with a relatively small number of nuclei (1,000) and increasing, higher amounts of Maxima H^−^ (100, 200, and 400 U) per 20 µL RT, sensitivity did not reach the level achieved with homebrew enzymes (Fig 7H; lanes 8, 7, and 9 versus 5; medians 1,109, 1,092, and 1,000 versus 1,296; Wilcoxon *q* < 0.0001). Notably, the highest amount (400 U) paradoxically reduced sensitivity compared with 100 and 200 U (Wilcoxon *q* < 0.001), possibly due to inhibitory components in the Maxima H^−^ storage buffer, which may limit its use at high concentrations during RT. Finally, sensitivity was restored only by replacing the commercial S.I. with homebrew rRi during RT and the pre-RT wash step (Fig 7H, lanes 6 versus 5; medians 1,260 versus 1,296; Wilcoxon *q* = 1). This is consistent with the fluorescent RNase assay (S2E Fig), which showed relatively low RNase inhibition by S.I. Thus, the ambient or residual RNase activity in liver nuclei required more potent inhibition, achievable by using our homebrew rRi during RT and the pre-RT wash. Overall, USPPAR was compatible with oligo phosphorylation, ligation, and RT using commercial enzymes in snRNA-seq of liver samples, as nuclei prepared with homebrew or commercial enzymes showed similar distributions across major clusters in 2D embeddings and similar cell type compositions (S12H and S12I Fig, Homebrew versus Commercial).

Finally, to assess whether identical cell types remain co-clustered across organs and lysis conditions, nuclei from three murine organs and two lysis systems were modeled together in 2D UMAP. Appropriate aggregation by cell types was observed, such as Krt19+ bile and pancreatic ducts, Cd68+ monocytes, Col1a1+ mesenchyme, Cdh5+ endothelium, Gypa+ erythroid cells, and tissue-specific clusters of hepatocytes and pancreatic acinar cells (S13 Fig). The co-clustering of identical cell types across different organs (ductular, blood, and mesenchymal cells) and lysis buffers (pH3 and CuC) highlights the robustness of this platform. Not only does the platform perform well with nuclei prepared similarly, but its successful integration with the dataset from whole-cell splenocytes further demonstrates its reliability across different sample sources, preparation methods, and barcoding and library preparation strategies.

In conclusion, unlike previous methods that required sorting incomplete subsets of individual cells from challenging organs such as the spleen [20–23], USPPAR efficiently and comprehensively captures native cell states in nuclease-rich organs without compromising sensitivity. This feat was achieved through a special combination of lysis buffer with a broad-spectrum nuclease inhibitor, fixatives, and gentle nuclear washes, maximizing the number and quality of RNA-containing nuclei.

### The extension to plant tissues demonstrates transkingdom compatibility with cell-wall-bearing tissues

To assess the broad compatibility of the system, we extended the animal-tissue protocol to roots and shoots of *Zea mays* seedlings. Unlike animal tissues, plant tissues have tough cell walls around their cells that hinder nuclear release. The varying cell wall compositions across different plant species make it challenging to find a universal method for releasing individual cells through enzymatic digestion (protoplasting). By contrast, direct one-pot nuclear lysis bypasses this limitation, allowing us to demonstrate the general applicability of our snRNA-seq system to plant tissues. Here, instead of using plastic pestles to crush murine tissues, Dounce homogenizers were employed to more effectively homogenize cell-wall-containing plant tissues (Fig 8A). Regarding nuclear extraction in plants, divalent cations (M^2+^) such as Mg^2+^ and positive-charged polyamines like spermidine are commonly used as nuclear stabilizers [56]. Thus, the lysis buffers containing M^2+^ (both Ca^2+^ and Mg^2+^) used for spleen nucleus extraction, along with a spermidine-based buffer, were separately employed to lyse maize roots (S2 Table, SampleID 6). Subsequently, the extracted nuclei were differentially barcoded (Barcoding stage) during RT to identify the better lysis formulation. Judged by the number of genes detected per barcode, the sensitivity was higher in root nuclei extracted with the buffer containing M^2+^ compared to those extracted with spermidine (Fig 8B, Roots, M^2+^ versus Spd). The discovery of a better formulation for extracting root nuclei underscores another advantage of this split-pool method, enabling the barcoding and comparison of different sample sources within the same experiment, thus minimizing batch bias.

To benchmark gene detection sensitivity, we used a reference whole-cell protoplast scRNA-seq dataset from maize roots generated with the 10× platform [26]. The number of genes detected with 2,500 sampled reads per nucleus closely approached the reference data obtained using whole cells (S14A Fig, USPPAR versus 10×_v3). When only nuclei prepared using the superior M^2+^ buffer were included, gene detection sensitivity was even more comparable (Fig 8C). This comparable gene-detection sensitivity indicated that nuclei isolation by direct lysis with the RNase-blocking complex could substitute tedious protoplasting, which requires a long processing time and species-dependent optimization. To test whether snRNA-seq can identify tissue-specific clusters, nuclei with more than 120 detected genes in this pilot dataset were clustered unsupervised. This revealed tissue-specific clusters, including the exodermis and meristem (L6), hair and epidermis (L7), vascular cylinder tissues (including the stele, endodermis, protophloem, and xylem; L1 and L8), pericycle (L2), and cap (L4) [26]. Besides characteristic marker expressions within each cluster, differential zonal expression of markers was also evident. For example, CASPL1A1 representing the endodermis, Zm00001d052034 representing the phloem, and Zm00001d008285 representing the stele were expressed in distinct subregions of the L1 (S14B Fig).

Due to the higher sensitivity of the M^2+^-based lysis buffer with roots above and the standard practice of excluding Ca^2+^ in procedures for plant nucleus isolation [56,57,58], likely attributed to the cell-wall strengthening effects of Ca^2+^ [59], we conducted formal snRNA-seq on maize shoots (S2 Table, SampleID 7 and 13) using a Ca²⁺-free M²⁺-based lysis buffer, equivalent to the mouse CuC buffer with Ca²⁺ replaced by EGTA. Additionally, the original gentle detergent mixture used for roots (0.1% NP-40, 0.1% Tween-20, and 0.01% digitonin) was replaced with 0.3% Triton X-100 to prevent the copurification of organelles, particularly green chloroplasts and possibly mitochondria, in the 30%–60% iodixanol interface. Compared to a reference snRNA-seq dataset prepared using the 10× platform [27], nuclei prepared with our method detected more UMIs and genes across all subsampled read counts per barcode in two independent experiments (Fig 8D). This improved sensitivity was likely the result of higher RNA retention in the shoot nuclei during nuclear extraction, since the detection sensitivities using USPPAR and 10× Chromium are similar when cultured cells were used as input (Fig 3). Moreover, multiplets were present at a very low level (0.2%) when reference nuclei from cultured human and mouse cells were included and prepared simultaneously (S15A Fig). To demonstrate the spectrum of cell types covered by the nuclear extraction system, the USPPAR dataset and a reference nuclear dataset using 10× Chromium were modeled together. In the co-embedded 2D UMAP space, the nuclei from 2 independent USPPAR experiments (Figs 8E, USPPAR; S15B, USPPAR1 and USPPAR2) integrated well with the reference nuclei (Figs 8E and S15B, 10×_v3), covering the cell types annotated in the reference. To further validate the independent informativeness of the USPPAR dataset, it was modeled and embedded in the 2D UMAP on its own. The inferred cell-type assignments remained clustered (S15C Fig, CellType) with the expression of corresponding canonical cell-type-specific markers (S15C Fig). The examples include: the mesophyll (PEPC1 and MDH6) and adjacent bundle sheath (DCT2); the vascular precursor tissue, procambium meristem (AIC2), with vascular marker (RTL2) and nearby small, separate clusters of differentiated xylem (NACTF103 and NACTF131) and phloem (zmSWEET12 and zmSUT1); the guard cell (KCH3) and stomatal precursor (EPF2); and different subclusters representing epidermal cells with differential marker expressions (GPAT12, RD22, KCS15, and Zm00001d025958) [27,60]. Thus, the lysis buffer containing the CuC also worked with cell-wall-containing plant tissues to extract RNA-harboring nuclei, providing single-cell transcriptomes of known cell types in maize seedlings with higher sensitivity compared to data obtained from the nuclei using the commercial 10× platform.

## Discussion

The key improvements in USPPAR are the complete tailing of ssDNA even if it ends in a recessed form and the nonconjugating Cu^2+^-chelator complex (CuC) that immediately and efficiently quenches nuclease activity that can be removed afterward.

Compared with the three other popular high-throughput methods that add PCR handles by template-switching (such as PIP-seq and SPLiT-seq) or directly tagmentation on double-stranded cDNA, USPPAR demonstrated improved gene-detection sensitivity. The increased sensitivity can be attributed to the highly efficient method of appending PCR handles, regardless of cDNA-end status, via TdT-mediated tailing in our engineered buffer. Unlike in current reaction systems, where monovalent cations (K^+^ and Na^+^) and the divalent alkaline earth metal ion (Mg^2+^) are ubiquitously present, their removal might disfavor the formation of DNA duplexes. Monovalent cations help to neutralize the negative charges on the phosphate backbone of the DNA, reducing the repulsive forces between the strands and stabilizing the duplex; Divalent Mg^2+^ ions coordinate with the phosphate groups, also stabilizing the DNA duplex structure through ion-mediated interactions. Thus, the removal of these stabilizing forces prevents the substrate ends from remaining bound to complementary strands or forming secondary structures, thereby favoring DNA extension by The unexpectedly low working concentrations of Co^2+^ and Mn^2+^ (0.75 mM), despite the high dATP concentration (2 mM) which does not chelate them, further reduce DNA stabilizing forces while simultaneously allowing for trace-free removal after the reaction. Beyond the high percentage of cDNA ends with the poly(dA) PCR handle, the long length of the homopolymer tail could also help concentrate primers by providing multiple local docking sites.

Besides serving as an integrated module in USPPAR, the high-efficiency tailing could replace the other PCR-handle appending steps in other platforms to further improve their sensitivity. In addition to serving for cDNA amplification for mRNA- and ncRNA-seq, the tailing system can also be easily adapted to other TdT-dependent techniques. For example, the ability to tail DNA regardless of its end structure for further primer-mediated amplification can be employed to amplify samples such as free DNA in blood and environmental samples, as well as in aptamer screening and on-chip signal amplification [61], to achieve reduced bias and higher coverage and sensitivity in detection. Furthermore, the ability to label recessed DNA ends can extend to improving the efficiency of labeling DNA nicks in cells, thereby enhancing the sensitivity of techniques such as TUNEL or CRISPR nick-site detection through high-throughput sequencing [62]. Overall, the simplicity and trace-free nature of this TdT reaction system make it straightforward to extend to other applications.

RNase depends on histidine residues for catalysis and becomes inactive when these residues are fully protonated [63], which explains the effectiveness of the pH 3 citrate buffer in extracting pancreatic nuclei. However, the requirement for very low pH limits the widespread application of this strategy, as changes in ionic bonds can denature proteins or dissociate them from their targets. The affinity of Cu^2+^ to histidine residues at higher pH values [64] indicates that the ion selectively targets the deprotonated residues. This binding to the deprotonated histidine renders RNases inactive regardless of the histidine’s protonation status, across acidic to neutral pH values. Besides the pH3 strategy, there are several other methods for blocking RNases, each with its own drawbacks. Vanadyl ribonucleoside [65] depends on divalent cations and potentially interferes with downstream enzymatic activities. Similar to recombinant RNase inhibitors, this nucleoside analogue is difficult to acquire in large amounts and is ineffective against samples containing various types of nucleases, particularly DNases. Reducing agents [66] require prolonged or high-temperature incubation, which is incompatible with one-pot lysis, where RNAs are immediately exposed to nucleases. The use of highly negatively charged polymers, such as polyvinyl sulfonic acid [67], to bind and inactivate RNases also competitively binds positively charged nuclear proteins and distorts the structure of unfixed nuclei. Furthermore, these large polymers are difficult to remove from the extracted nuclei, which complicates downstream enzymatic reactions. Finally, DEPC carboxymethylates RNA purines [53] and readily reacts with amino acid side chains, particularly lysine, competing with crosslinkers such as formaldehyde and NHS esters for efficient crosslinking [48]. In contrast to the shortcomings of the strategies mentioned above, CuC remains active up to neutral pH and effectively blocks both RNase and DNase. It is immediately active at low overall salt concentrations, easy to remove from nuclei by washing with low concentrations of strong chelators such as EDTA, and free from protein or nucleic acid modifications. Additionally, it is compatible with downstream RT and ligation enzymatic reactions. These features also make this complex a potential component in other methods, such as microarray, in situ hybridization, hybridization-mediated RNA enrichment, RNA immunoprecipitation, and systems requiring reversible inhibition of nuclease activities.

Although the CuC is a potent RNase inhibitor, it is non-covalent and therefore transient. Because copper also inhibits downstream enzymatic reactions, it must be washed away. However, if RNases are not removed during this wash, the RNA may be degraded before or during RT. The presence of residual RNases in nuclei is demonstrated by the increased number of genes detected per liver nucleus when the less potent Superase.IN is replaced with our homebrew RNase inhibitor. This limitation is addressed by the discovery of two compounds, EGS and ABF, that conjugate to lysine residues and inactivate RNase. This chemical-conjugation-mediated inhibition is expected to be permanent. When performed in methanol containing residual CuC, the permanent RNase inactivation ensures that RNA remains protected during pre-RT washes and RT, even after CuC is removed. The success of lysine-conjugation–mediated inactivation is evident from the significantly improved sensitivity in liver nuclei, especially at higher nuclear numbers per reaction during the Barcoding stage. Overall, rapid RNase inhibition by CuC combined with permanent inactivation by EGS or ABF ensures continuous RNA protection from sample preparation through final RT.

The CuC lysis system offers several advantages over pH3. First, it is compatible with methanol-based fixation, which is a good non-RNA-modifying fixative, and allows long-term storage without freeze–thaw damage. Second, although we used pH 4.5 to avoid RNase A’s optimal activity, mRNA protection remains robust up to physiological pH 7. This pH tolerance provides flexibility for experiments requiring higher pH, such as minimizing RNA secondary structure changes, preventing protein denaturation, or preserving molecular interactions. Third, building on this pH flexibility, the broader pH range also accommodates chemical reactions or modifications that require less acidic conditions. For example, lysine conjugation using PFA, EGS, or ABF is more effective at higher pH, where reduced lysine protonation facilitates the reaction. These additional conjugations improve detection sensitivity, as shown by side-by-side comparisons using liver and PBMC samples. Finally, CuC-based lysis produces cellular compositions of different spleen cell types that closely match those in the reference mouse splenocyte dataset.

Although CuC lysis demonstrated several theoretical and practical advantages in the organs examined here, no method provides a true ground truth for cell type composition, and it was not feasible for us to profile all tissues from all organisms with both pH3 and CuC buffers. Therefore, we cannot claim that CuC is universally superior to pH3 or the sole optimal solution for all tissues. Instead, we provide a defined framework for the Dissociation stage: Pulverization, Lysis, Purification, Wash, and Fixation. Among these steps, Lysis offers the greatest flexibility. Examples include pH3 versus CuC lysis, mechanical disruption with a Dounce homogenizer or plastic pestle, and different detergents. First, although pH3-based lysis has the disadvantages described above, it may be more effective for matrix-rich tissues, where acidic conditions can help dissolve collagen or calcium phosphate in the bone matrix and release osteocytes. Further, the pronounced mesenchymal/endothelial-to-lymphocytic bias in the pH3 system may be advantageous for enriching and characterizing mesenchymal or endothelial cell populations. Finally, pH3 appeared to preserve RNA integrity better than the CuC buffer (S9D Fig). Through further testing, compatibility with crosslinking agents could be improved by adjusting fixation time, temperature, or concentration, and pH3 could also be adapted to work with methanol fixation. With these changes, pH3 might offer higher sensitivity and reduce the lymphocyte loss seen with the original pH3 lysis method. Second, Dounce homogenizers are suitable for breaking down tissues with tough matrices or cell walls during lysis, but their strong mechanical force may damage nuclei. Third, the selection of detergents during the lysis stage can significantly affect nuclei recovery, with different detergents being optimal for different tissues, as noted in PanSci [13]. Thus, it is advantageous to leverage USPPAR’s strengths. First, USPPAR snRNA-seq data integrate well across the same cell types from different organs and dissociation strategies, demonstrating robustness to variations in dissociation parameters. Second, because the dissociation stage consists of five well-defined steps, multiple parameter combinations can be tested in parallel. Third, nuclei from these different conditions can be multiplexed during the first round of RT, as demonstrated in our datasets. Finally, sequencing a small aliquot from a single pilot experiment allows efficient identification of optimal conditions for new or unfamiliar samples at minimal cost. Overall, the defined five-step Dissociation workflow, adjustable parameters, and sample multiplexing make USPPAR highly flexible for optimizing nuclei preparation across diverse tissues.

Besides RNA, the preservation of nuclei also archives information like DNA sequence/modification/breakages, histone modifications, and possible nuclear/chromatin structures. The CuC system works with both conjugators and methanol fixatives, allowing further nuclear stabilization in a safe environment. As a result, nuclei can also be subjected to fixation, barcode hashing [68], or antibody detection in any order for subset enrichment, sample multiplexing, or RNA+ multimodal profiling. Additionally, fixing nuclei in an organic solvent allows them to be shipped easily at ambient temperature. Besides, the long-term storage of the nuclei in liquid form avoids the requirement of aliquots. Not only can nuclei be stabilized and archived using the complex, but so can free nucleic acids from biopsies, blood, and frozen tissues used for clinical diagnoses. Additionally, the complex is suitable for stabilizing and archiving environmental samples and organisms from seawater, soil, or feces for ecological studies. The ability to handle frozen specimens, perform further fixation, and gently wash nuclei indicates the broad applicability of the extraction system to a wide range of potential samples, including those kept in water-soluble OCT or in paraffin blocks [69]. Additionally, along with position-dependent multiplexing, the high detection sensitivity makes USPPAR suitable for high-throughput cell-state assays using the transduction or transfection of barcoded genes/sgRNAs in multi-well plates. Finally, USPPAR’s capability for one-pot collection of stabilized nuclei from tissues also makes it suitable for unbiased high-throughput in vivo screening with pooled libraries.

Compared with SPLiT-seq (S3 and S4 Tables, SPLiT-seq), the first system to use the split-pool barcoding strategy, now commercialized as Evercode kits (Parse Biosciences), the USPPAR system has several advantages. First, although the Evercode kits demonstrate higher sensitivity than the original version, most components in the newer releases (v2 or v3), such as fixatives and reagents used for barcoding and library preparation, are now undisclosed. In contrast, the USPPAR system is fully transparent yet still achieves higher sensitivity than the current commercial kit (v3) when applied to PBMCs. Beyond reducing costs, the open-source nature of USPPAR allows users to further improve the system by fine-tuning its components and parameters freely. Second, SPLiT-seq did not emphasize or characterize the Dissociation stage, making it necessary to use different procedures or commercial nuclear extraction systems for different tissue types. As a result, each tissue may require a distinct protocol, which can introduce procedure-dependent bias and complicate the integration of scRNA-seq data across specimens. Finally, at the amplification stage, SPLiT-seq requires several additional purification steps compared with USPPAR, including streptavidin–biotin capture after lysis, size selection after cDNA fragmentation, and post-ligation size selection. These extra steps increase hands-on time and may reduce the final library yield, as well as alter cDNA representation due to procedure- and operator-introduced artifacts.

EasySci (S3 and S4 Tables, EasySci), the successor to sci-RNA-seq3, is a high-input snRNA-seq method that provides an end-to-end workflow from samples to final library and has been demonstrated in at least 14 organs [13]. However, two major organs, the RNase-rich spleen and pancreas, were not included in its demonstration, despite the use of the well-known RNase quencher DEPC during tissue lysis, and sorting was applied to purify nuclei. Further, EasySci uses a fixed three-round barcoding scheme, consisting of one RT, one ligation, and final indexing PCR, totaling three rounds of barcoding. In contrast, standard USPPAR and SPLiT-seq perform three or more barcoding rounds plus an additional step after tagmentation, providing greater flexibility and scalability. Beyond scalability, EasySci’s limited combinatorial barcoding rounds create a trade-off between barcode diversity and reaction robustness: with the first two rounds using 384 wells each, EasySci generates 147,456 combinations before the final indexing PCR, allowing 8,000–10,000 cells to be pooled with low collision rates (~5%). However, this requires high reagent use and labor. Reducing to 96 wells per round (9,216 combinations) forces lower cell input per tagmentation reaction (<500 cells) to maintain low collision rates. Such low input leads to over- or under-fragmentation during tagmentation, as this step is highly sensitive to input DNA concentration, ultimately compromising sequencing quality. Finally, EasySci eliminates cDNA preamplification, replacing approximately 270 min of preamplification used in USPPAR with about 105 min of second-strand synthesis. Consequently, the first of EasySci’s two DNA purification steps is performed on unamplified second-strand products, increasing the chance of cDNA loss. In contrast, USPPAR performs both purifications on amplified cDNA, minimizing cDNA loss. This difference may explain the higher sensitivity of USPPAR compared with EasySci in liver datasets. Finally, EasySci is less self-sufficient than USPPAR because it requires specially modified oligos, in addition to phosphorylation, and relies entirely on commercial enzymes.

There are limitations associated with USPPAR. First, the isolation of nuclei from organisms or tissues inevitably leads to the loss of cytosolic mRNA. Although the information derived from nuclei was comparable to that from cells in most cases [70], the RNA and corresponding gene counts are usually lower with nucleus sequencing. Next, the mRNAs of non-polyadenylated genes, such as some histone mRNAs, and prokaryotes will be detected less effectively compared with the polyadenylated ones. Although the random 9-mer used during RT could ameliorate the loss of detection, this issue will be evident with short and highly modified transcripts. Polyadenylating all transcripts with poly(A) polymerase [14,71] before RT would be a remedy. In line with this, the issue of losing short transcripts with our non-crosslinking fixatives could be lessened by using crosslinkers like PFA or 1-Ethyl-3–3-dimethyl-aminopropyl carbodiimide [72]. Besides, it is time- and labor-consuming with split-pool barcoding regarding multiple rounds of ligation and washes, which results in inevitable cell and cDNA losses. The potential rescue would be the single-step barcoding using dissolvable polyacrylamide beads [73] after templated emulsion with cells [9]. Further, due to the nonselective nature of USPPAR, rare cell types (e.g., islet cells in the pancreas) in tissue might be obscured by abundant ones (e.g., acinar cells). This issue could be addressed by targeted sorting of known rare cell types or by selective reduction of abundant transcripts in the libraries [74]. Additionally, while cDNA amplification and RNA protection strategies could be extended to current spatial transcriptomic methods [75], the spatial information of single cells in tissues is lost with USPPAR during nuclear isolation. One potential solution to reveal cellular interactions could involve using crosslinkers to retain neighboring cytosolic fragments to allow for inferring cellular adjacencies based on exon-enriched cytosolic barcodes using intron-rich nuclear ones as the reference. Finally, we have not tested USPPAR with samples with mineral matrices, such as bones and teeth, with dense/abundant matrices, such as tendon and cartilage, or with thick capsules/shells, such as eggs of aqueous animals. Although minerals could be chelated by EGTA, an ingredient in our nuclear isolation buffer, and the extracellular materials are not expected to be a problem when the frozen samples are ground to fine powders, it would be interesting to try USPPAR on these tough, untested samples.

## Conclusions

Our systematic optimization of sample preparation, cDNA tailing, and single-tube amplification enabled the one-pot collection of single-cell transcriptomes from diverse sample types. Building upon the inherent advantage of high cell capacity through split-pool barcoding, this method combines several additional benefits, including low cost (S4 Table), low multiplet rates, equipment-free operation, and high sensitivity, which are not available with other current methods.

## Materials and methods

### Ethics statement

Collection of PBMCs was conducted in accordance with institutional and national ethical guidelines. Peripheral blood mononuclear cells (PBMCs) were obtained from a healthy volunteer after written informed consent was obtained. The study protocol was approved by the National Cheng Kung University Hospital Institutional Review Board (approval number B-ER-109-024).

Mouse tissue collection was conducted in accordance with institutional and national guidelines for the care and use of laboratory animals. All procedures were approved by the National Cheng Kung University Animal Care and Use Committee (approval number 104136).

### Materials

MS5 (ACC 441, DSMZ); 293T (ThermoFisher); OP9-DL1 (a kind gift from Juan Carlos Zúñiga-Pflücker [76]); iPS-DF19-9-7T (WiCell); D-MEM/F-12 (ThermoFisher, 12500-096); IMDM (ThermoFisher; 12440-053); αMEM (ThermoFisher, 12000-014); FBS defined (HyClone, SH30070.03); Polyvinyl alcohol (PVA; 87%–90%-hydrolyzed; Sigma, P8136); OptiPrep (iodixanol 60%; ProteoGenix, 1114542); SUPERase·In (Thermo Fisher, AM2694); M-MuLV Reverse Transcriptase (NEB, M0253S); SuperScript II Reverse Transcriptase (Thermo Fisher, 18064014);); Maxima H Minus Reverse Transcriptase (ThermoFisher, EP0752); T4 PNK (Enzymatics, Y9040L); T4 DNA ligase (Lucigen, F83911-1, 120 U/μL); EGS (CovaChem, 13308-100); Terminal deoxynucleotidyl Transferase (TdT, Qiagen, P7070L); PEG8000 (Sigma, P2139); Sepharose CL-6B (GE Healthcare, CAS 62610-50-8); HindIII-HF (NEB, R3104S); ThermoPol buffer (NEB, B9004S); carboxylate paramagnetic particles (Cytiva, 65152105050250); Halt protease inhibitor (ThermoFisher); proteinase K (MERCK, 1245680100); KAPA HiFi (Roche, KK2102); SYBR Green I, 10,000× (Lumiprobe, 20010); benzonase (BenzNuclease, BEE-N3116, ACROBiosystems); micrococcal nuclease (NEB, M0247S); RNase A (NEB, T3018L); mouse tissues were obtained from spare organs sacrificed by other laboratories (not specifically for this experiment; approved by IACUC); oligo-synthesis service (GENERAL BIOL, China).

### Preparation of his tag-binding resin

The preparation was modified from an established procedure [77]. Briefly, 853 mg of EDTA dianhydride, 111 μL of ethylene diamine, and 462 mg K_2_CO_3_ were added to 15 mL of DMSO and vortexed at ambient temperature overnight to obtain mixture A. Meanwhile, 25 mL of Sepharose CL-6B was added with water to 32 mL and, subsequently, added with 4.26 mL of NaOH (7.5 M) and 3.2 mL of epichlorohydrin, followed by shaking at ambient temperature for 2 hours. After the removal of supernatant by dripping from the resin in a column, the resin was washed 2 times with 50 mL of water and then added with 4.27 grams of ammonium chloride, 6 mL of ammonia, and water to reach a final volume of 40 mL. After shaking at ambient temperature overnight, the resin was washed 4 times with 5 bed volumes of water, followed by an additional 4 washes with the same volumes of DMSO. After dripping away DMSO, the resin was added to the whole mixture A and shaken overnight. Following the removal of supernatant by dripping, the resin was sequentially washed 2 times with 25 mL of DMSO, 2 times with 25 mL NaOH (0.3 M), 2 times with 25 mL of water, 1 time with 50 mL of NiCl_2_ (50 mM), and 2 times with 50 mL of water to acquire the immobilized metal affinity chromatography (IMAC) resin.

### Protein expression and purification

Rosetta 2(DE3)pLysS (Novagen) served as the host for the induction of his-tagged PNK, T4 DNA ligase, M5 RT, Rat RNase inhibitor, and C3013 (NEB) for TdT. These plasmids were cloned based on gene synthesis and Gibson assembly. The bacteria were shaken at 250 rpm in 2 mL of P-0.5G medium [78] containing 100 μg/mL of carbenicillin at 37°C overnight. The overnight growth was inoculated into 250 mL of ZYM-505 containing the identical concentration of carbenicillin and amplified for an additional 1.5–3 hours until A_600_ reached approximately 0.4. At that point, the temperature of the shaker was set to 25°C. After shaking for 30 min at the temperature, the bacteria’s growth was supplemented with IPTG to a concentration of 0.4 mM. The induction was carried out for 4 hours before pellet collection by centrifugation at 3,000*g* for 10 min at 4°C.

Bacterial pellets were lysed with 40 ml of lysis buffer composed of HEPES pH 7.2 (20 mM), NaCl (0.5 M), TCEP (1 mM), octyl glucoside (1%), and PMSF (20 μg/mL). After brief dispersion by pipetting, the lysates were shaken on ice for 10 min and clarified by centrifugation at 10,000*g* for 10 min at 4°C. The supernatant was further filtered through a PES syringe filter (0.45 μm pore size) and loaded onto a 500 μL bed volume of the IMAC resin that was washed and balanced with the lysis buffer. The binding, washing, and elution steps were carried out in a refrigerated environment to maintain the cold temperature throughout the purification procedure. After the supernatant slowly dripped away from the column equipped with a 25G needle beneath, the resin was washed twice with 10 bed volumes of the lysis buffer, with octyl glucoside replaced by Tween 20 (0.1%). The proteins were eluted using 2.5× bed volumes of the wash buffer without PMSF but containing imidazole (250 mM). The eluted fractions harboring high concentrations of the target proteins based on SDS-PAGE were pooled and dialyzed in 500 mL of HEPES pH 7.2 (20 mM), KCl (200 mM), EDTA (0.1 mM), and TCEP (1 mM) overnight. The dialyzed proteins were supplemented with equal volumes of glycerol, quantified for concentration using Pierce 660, and stored at −20°C or −80°C for short or long terms, respectively.

The induction and purification of Tn5 using pTXB1-Tn5 [31] for tagmentation followed the respective original references.

### Preparation of PCR products with blunt and 3′-recessed ends

The multiple-cloning site of pBluescript KS(+) was amplified using M13 forward and reverse primers (S1 Table) with KAPA HiFi. The reaction mixture (200 μL) contained 1× KAPA HiFi HF buffer, 0.2 mM dNTP mixture, 200 nM each of M13 primers, 2 μL KAPA HiFi enzyme, and 100 ng of template DNA. The reaction was heated at 95°C for 30 s, followed by 30 cycles of 95°C for 10 s, 55°C for 15 s, and 72°C for 30 s, and concluded with a final extension at 72°C for 10 min. After PCR purification with a silica column, the PCR product (900 ng) was digested with 0.5 μL of HindIII-HF in a 20 μL reaction containing 1× CutSmart Buffer at 37°C for 30 min. After purification using a silica column, the eluent served as the substrate, containing both blunt ends (the original PCR product ends) and 3′-recessed ends (the new ends produced by HindIII digestion).

### Denaturing urea polyacrylamide electrophoresis

The gels were prepared by mixing a gel solution containing acrylamide/bis-acrylamide (29:1) at the specified concentrations, TEMED (0.16% v/v), ammonium persulfate (0.05% w/v), urea (8 M), Tris-borate (pH 8.3, 89 mM), and EDTA (2.5 mM). The samples were mixed with an equal volume of 2× sample buffer (95% formamide, 0.025% SDS, 0.025% bromophenol blue, 0.025% xylene cyanol FF, 0.5 mM EDTA), heated at 95°C for 10 min, and then subjected to electrophoresis at 180 V in a water bath maintained at 55°C. Electrophoresis was performed until the bromophenol blue dye front migrated to approximately 2/3 of the gel length.

### Silver staining for polynucleotides

PAGE gels were fixed in ethanol (10%) and acetic acid (0.5%) for 5 min, rinsed with water, and incubated in staining solution composed of silver nitrate (0.15%) and formalin (0.15%) for 10 min. After the incubation, the gels were washed twice with water and developed in NaOH (375 mM) and formalin (0.3%) for 10 min. The development was stopped with water.

### Preparation of oligos for ligation and split-pool barcoding

For oligos requiring 5′ phosphorylation, 10 μL of 100 μM substrates were treated with PNK (50 ng) in 20 μL of reaction (Tris-HCl pH 7.5 70 mM, MgCl_2_ 10 mM, DTT 5 mM, ATP 1 mM) at 37°C for 2 hours, followed by heat inactivation at 65°C for 20 min. The reaction was added with 20 μL of EDTA (11.6 mM) and further diluted to the desired concentrations with loTE (Tris-HCl pH 8, 10 mM, EDTA 0.1 mM). To prepare annealed adapters, the PNK-treated or untreated oligos (25 μM each) were annealed in annealing buffer (Tris-HCl pH 8 20 mM, potassium acetate 40 mM, EDTA 0.1 mM) using the annealing program (94°C denaturation for 1 min, followed by a stepwise 1°C decrease per minute from 80 to 20°C) and further diluted with the annealing buffer. For oligonucleotide phosphorylation using commercial T4 PNK, 1 U of enzyme was used per 0.5 nmol of oligos in a final reaction volume of 10 µL, in the same buffer and reaction conditions as used with homemade PNK.

### Cell culture

MS5 and 293T cells were cultured in DMEM/F-12 medium supplemented with fetal bovine serum (FBS) (10%). OP9-DL1 cells were cultured in αMEM supplemented with FBS (20%). The FBS-containing media were replenished every 2 days, and the cells were dissociated with trypsin for replating every 4 days. The hPSCs were cultured in an E8 medium composed of E6 plus TGFβ1 (2 ng/ml) and zbFGF(115, 116) (100 ng/ml) with daily medium change [79]. The cells were dissociated with Accutase and neutralized with PBS containing PVA (0.1%) (PBSPVA) before centrifugation and resuspension with PBSPVA for cell counting and seeding. The hPSCs were seeded in the E8 medium supplemented with Y-27632 (10 μM) in wells coated with rhVTN-NC (2.5 μg/cm^2^).

### RT-qPCR

To benchmark the homebrew M5 reverse transcriptase against the commercial ones in S2C Fig, the cells (100 cells/μL) were lysed with a lysis buffer composed of Triton X-100 (0.2%) and proteinase K (200 μg/mL) at 37°C for 1 hour. cDNA synthesis was performed in a 20 μL reaction composed of the RNA lysate (5 μL), 0.2 μL of PMSF (50 mM in DMSO), 1× RT buffer (Tris-HCl pH 8.3 50 mM, KCl 75 mM, MgCl_2_ 3 mM, DTT 5 mM,), dNTP (final 0.5 mM each), homemade rat RNase inhibitor (100 ng), PVA (0.05%), T23VN (1 μM), and reverse transcriptases at 42°C for 20 min and 85°C for 5 min. Ten μL of the cDNA was used as the input for a triplicate of qPCR reactions (20 μL each). To demonstrate the protection of mRNA using CuC in Fig 5E, all of the washed pellets were used for lysis, RT, and qPCR as detailed in the figure legend.

### Fluorescence assays to detect nuclease activity

To detect nuclease activity, an RNaseAlert oligo composed of chimeric DNA–RNA–DNA, with a green fluorescent molecule at one end and a quencher at the other, was used. Standard reactions were performed in a final volume of 25 μL with pH 7.2 lysis buffer containing 50 nM of the reporter. Various inhibitors, pH levels, additives, and any deviations from the standard reaction conditions are specified in the respective figure legends. After incubating at the specified temperatures and times, the reactions were diluted to 150 μL with the same reaction buffers, excluding substrates, lysate, and nucleases, for fluorescence quantification in triplicate of 50 μL. Fluorescence detection was then performed in triplicate using a SpectraMax iD5, with excitation at 490 nm and emission at 520 nm.

The crude pancreatic lysate was prepared by lysing 10 mg of murine pancreas in 400 μL of lysis buffer (HEPES pH 7.2 20 mM, NaCl 146 mM, CaCl_2_ 1 mM, MgCl_2_ 21 mM, Tween 20 0.1%, NP-40 0.1%, and digitonin 0.01%). The lysis was performed by bead beating (Precellys, 10 seconds, 4,500 rpm) in 2 ml plastic tubes containing 6 glass beads of 2.5 mm diameter. The lysate was subjected to centrifugation at 17,000 *g* for 1 min before the cleared nuclease-laden supernatant was kept for the assays.

The Cu²⁺-citrate complex was prepared by adding citric acid in an equal molar ratio to 500 mM CuSO₄, adjusting the pH to 4.5 or 7.2, and then diluting the mixture with water to achieve a final concentration of 250 mM. The Cu²⁺-NTA complexes were prepared as a stock solution at pH 4.5 because severe precipitation occurred at pH 7.2. One exception is the experiment shown in S9D Fig, where concentrated CuSO₄ was added separately to the reactions, followed by the addition of the respective chelators at 500 mM (NTA and EDTA) or 1,000 mM (citrate) from their stock solutions.

### Procedure for collecting PBMCs

PBMCs were obtained from a healthy volunteer donor (NCKUH IRB B-ER-109-024). Approximately 4 mL of fresh blood was mixed with 3 mL of PBS and layered over 2.5 mL of Ficoll-Paque Plus. The samples were centrifuged at 1,200*g* for 20 min at 20°C. After removing most of the top plasma layer, the PBMCs at the interface were collected and washed twice with 50 mL of Ca²⁺- and Mg²⁺-free PBS containing 0.1% PVA, centrifuging each time at 250*g* for 10 min at 20°C. The resulting pellet was resuspended in ~135 µL of PBS to make a final volume of 150 µL. Ice-cold methanol (10× volume, 1,500 µL) was added to fix the PBMCs. After incubation at −20°C for 30 min, the cells were transferred to −80°C for long-term storage.

### Synthesis of 4-azidobenzoyl fluoride (ABF)

The procedure was performed according to the published protocol [44]. Azidobenzoic acid (30 mg, 1 eq., 0.184 mmol) was added to 900 µL of acetonitrile and mixed with pyridine (14.8 µL, 1 eq., 0.184 mmol). The mixture was vortexed at 25°C until a homogeneous suspension was obtained. Cyanuric fluoride (21.36 µL, 1.35 eq., 0.248 mmol) was then added, and the reaction mixture was vortexed at 25°C for 16 h. The reaction was quenched by adding 333 µL of ice-cold water, followed by brief vortexing. Subsequently, 833 µL of diethyl ether was added, and the mixture was briefly vortexed again. After centrifugation at 1,000*g* for 30 s, the upper ether layer containing ABF was collected. The ether phase was washed twice with 150 µL of water and twice with 150 µL of saturated NaCl solution (brine), with brief vortexing and centrifugation after each wash to separate the layers. The final ether layer was dried by adding 20 mg of anhydrous MgSO₄ and vortexing for 2 min, followed by centrifugation at 1,000*g* for 1 min. The solvent was removed using a Concentrator Plus (V-GV program, 45°C) for ~1 h, yielding the final ABF product. ABF was dissolved in dimethylacetamide to a final concentration of 100 mM and stored at −80°C for long-term storage.

### Test for pH- and chelator-dependent precipitation of Cu²⁺

The mixtures of CuSO₄, buffers, citrate, and BSA at various concentrations and pH levels are described in the legend of Fig 5A. Triplicates of the mixtures were aliquoted into a 96-well plate and quantified for optical density (600 nm) using a SpectraMax iD5. The plate was also scanned with a scanner for direct precipitation visualization.

### Cell preparation for USPPAR

The cultured cells were dissociated with Accutase, neutralized with an equal volume of PBSPVA, and washed again with the same volume of PBSPVA, with centrifugation in between at 3,00*g* for 5 min. The pellets were resuspended with 20−40 μL of PBS, fixed by adding 10× volumes of methanol at −20°C, and permeated/fixed at −20°C for at least 60 min before further use.

For PBMCs, the required volume (11 µL) of cells in methanol was taken. Eight times this volume (88 µL) of ice-cold methanol was added to the fixed PBMCs. Then, 1/99 volume (1 µL) of 100 mM ABF was added and mixed to achieve a final concentration of 1 mM. The mixture was incubated at 4°C for 2 hours prior to the Barcoding procedure, following the same protocol as for cultured cells.

### Mouse nuclei preparation for USPPAR

Pancreas, livers, and spleens were harvested from young mice of less than 3 weeks old (IUCAC approved) and immediately frozen in liquid nitrogen until use. For the initial pH3-based lysis, the tissue fragments were frozen in folded polyimide films in liquid nitrogen for at least 10 min, crushed using an aluminum block on a stainless iron plate submerged in liquid nitrogen to maintain the low temperature (Pulverization step), immediately lysed with an ice-cold lysis buffer (citrate pH 3 25 mM, sucrose 250 mM, 800 μL per ~50 mg), and further ground using plastic pestles in microcentrifuge tubes. The lysates were filtered through an 800-mesh nylon membrane (Lysis step) and layered over 500 μL of citrate pH 3 (5 mM), iodixanol (30%), and PVA (0.1%) with 10 μL of iodixanol (60%) as the bottom cushion. After centrifugation at 2,000*g* for 5 min, the nuclei were aspired from the iodixanol 30%−60% interface (Purification step), washed 2 times with 200 µl of acidic wash buffer (citrate pH 3 1 mM, PVA 0.05%) (Wash step), fixed with 320 μL of a fixation buffer (citrate pH 3 50 mM, PFA 1%) at 4°C for 10 min (Fixation step). The nuclei were centrifuged and washed 2 times with 200 μL of 1× RT buffer containing PVA (0.05%) and rat RNase inhibitor (5 ng/μL) before RT.

For CuC-based lysis of the spleen, the splenic fragments were crushed, lysed, and ground in the same way except that the CuC buffer (HEPES pH 4.5 20 mM, NaCl 146 mM, CaCl_2_ 1 mM, MgCl_2_ 21 mM, Cu-citrate 10 mM) containing a detergent mixture (Tween 20 0.1%, NP-40 0.1%, and digitonin 0.01%) [17] was used for lysis. The Cu-citrate solution was prepared by mixing CuSO_4_ (0.5 M) with sodium citrate at a 1:1 molar ratio to form a 250 mM stock solution. After the filtration, the filtrates were layered over the same buffer, replacing the detergent mixture with PVA (0.1%) and iodixanol (30%), with 20 μL of iodixanol (60%) as the cushion at the bottom. After centrifugation at 2,000*g* for 5 min, the nuclei were collected from the iodixanol 30%−60% interface, washed twice with 200 μL of the CuC buffer in a column bearing a hydrophilic PTFE membrane (3 μm pore size), and fixed with 200 μL of the CuC buffer containing PFA (0.125%) at 4°C for 15 min. The nuclei were added 10 μL Tris-HCl (pH 8, 1 M) to quench the PFA, fixed directly by adding 10× volumes of methanol containing 5 mM final of MgCl_2_ (methanol/MgCl_2_) at −20°C and remained at −20°C until downstream usage.

For CuC-based lysis of the pancreas and liver, the procedure was essentially identical to that used for the spleen, except that yeast tRNA (50 ng/µL), rat RNase inhibitor (5 ng/µL), and Halt protease inhibitor (1×) were included during Lysis and Wash. The protease inhibitor was omitted during the wash steps, and no PFA fixation was performed prior to methanol fixation. To demonstrate the effects of EGS and ABF, the required volume of nuclei in methanol/MgCl₂ solution was added with PVA to a final concentration of 0.1%, and the sample was centrifuged at 1,000*g* for 1 min. The supernatant was removed, leaving 90 µL of nuclei-containing solution. Then, 10 µL of 100 mM EGS or 10 mM ABF (diluted from the 100 mM stock) in DMSO was added to resuspend the nuclei. The Conjugation was performed at 4°C for 2 hours before the Barcoding stage.

### Assessment of RNA quality and DNA staining in nuclei extracted from three mouse organs after pH 3 or CuC lysis

To assess reduced RNA degradation in nuclei extracted using pH 3 and CuC lysis systems, three mouse organs were pulverized, and the resulting powders were processed using the respective lysis strategies through the Lysis and Purification steps described for the spleen above. Purified nuclei from the 30% to 60% iodixanol interface were washed four times with 200 µL of the corresponding wash buffer containing 0.1% PVA, using centrifugation at 1,000*g* for 1 min per wash. The control without inhibitors (“None”) consisted of the CuC-based lysis buffer lacking the Cu-citrate complex.

To extract RNA from nuclear pellets, 300 µL of TRIzol was added to lyse the nuclei. An equal volume of ethanol (300 µL) was mixed by pipetting, and the mixture was applied to Qiagen miniprep spin columns for RNA binding. Columns were washed sequentially with 500 µL of Qiagen PB buffer and 80% freshly prepared ethanol. RNA was eluted in 30 µL of water, with centrifugation at 17,000*g* for 1 min performed between binding, 2 washes, and elution. The eluted RNA was assessed using a Qsep RNA cartridge.

One-hundredth (2 µL) of the nuclei suspension after the third wash above was added to 50 µL of PBS containing 0.1% PVA and 10 ng/µL Hoechst 33,258. After centrifugation at 1,000*g* for 1 min, the nuclei were resuspended in the same buffer for fluorescence microscopy imaging of nuclei extracted using pH 3- and CuC-based lysis.

### Plant nuclei preparation

The corn seeds were allowed to germinate on wet tissue paper for 4 days. The root or shoot fragments were frozen, crushed, and lysed in the same way as with the splenic fragments, except that 3 versions of buffers were used. For roots, a spermidine- (HEPES pH 4.5 20 mM, NaCl 40 mM, KCl 90 mM, EDTA 2 mM, EGTA 0.5 mM, and spermidine 0.7 mM) or a divalent cation-containing (HEPES pH 4.5 20 mM, NaCl 146 mM, CaCl_2_ 1mM, MgCl_2_ 21 mM, and EGTA 0.5 mM) buffer containing Cu^2+^-citrate (10 mM, diluted from 0.5 M of Cu-citrate) was used for lysis. The lysates were further Dounce homogenized for 20 strokes to release nuclei. After Dounce homogenization, Triton X-100 (0.3%) was used as a detergent to remove the abundant green chloroplasts from the 30% to 60% iodixanol interface during density-gradient purification. The nuclei were purified with 30%−60% iodixanol in respective buffers as with the splenic nuclei, washed 2 times with 200 μL of respective buffer containing Cu-citrate (10 mM) and PVA (0.05%) by centrifugation at 250*g* for 1 min, resuspended with 20 μL of the same buffer containing Cu-citrate (10 mM), and fixed by adding 10× volumes of methanol/MgCl_2_ at −20°C for at least 30 min at −20°C. The collection of shoot nuclei was identical to that of the root ones, except that a Ca^2+^-free divalent cation-containing buffer (HEPES pH 4.5 20 mM, NaCl 146 mM, MgCl_2_ 21 mM, and EGTA 0.5 mM) was used.

### First-round barcoding based on RT

The required number of cells in the fixative was taken, PVA was added to a final concentration of 0.1%, and the cells were centrifuged and washed twice with 200 μL of low-salt buffer (Tris-HCl pH 8 10 mM, KCl 1 mM, EDTA 0.1 mM, PVA 0.1%) containing rat RNase inhibitor (5 ng/μL). The cells were resuspended in the same buffer and aliquoted into a 96-well PCR plate for first-round barcoding (4,000 cells per 20 μL) using RT: Tris-HCl pH 8.3 50 mM, KCl 75 mM, MgCl_2_ 3 mM, DTT 5 mM, dNTP 0.5 mM each, homemade rat RNase inhibitor 5 ng/μL, PVA 0.05%, PEG8K 0 or 7.5% as specified in the summary S2 Table, M5 reverse transcriptase 5 ng/μL, barcoded T16V 1 μM, barcoded N9 0 or 1 μM as specified in S2 Table. The reaction was performed by rotating the whole plate at 42°C for 40 min and stopped by adding 0.3 μL of EDTA 0.5 M to each well. With commercial Maxima H Minus reverse transcriptase, 10–400 U per 20 µL reaction were tested, as specified in the respective figure legends. For commercial SUPERase·In, 0.25 U/µL was used to replace each 5 ng of homebrew rat RNase inhibitor during all enzymatic incubation and wash steps.

### Subsequent rounds of barcoding based on ligation

The stopped reactions were pooled into 4–8 microtubes, centrifuged, and washed twice with 200 μL of the low-salt buffer containing PVA (0.1%) and rat RNase inhibitor (0.5 ng/μL) by centrifugation at 1,000*g* for 1 min. The pellets were resuspended in the wash buffer and aliquoted for 20 μL of the following ligation reactions: Tris-HCl pH 7.5 70 mM, MgCl_2_ 10 mM, DTT 5 mM, ATP 1 mM, PVA 0.1%, rat RNase inhibitor 5 ng/μL, T4 DNA ligase (4 ng/μL homebrew or 2 U/μL commercial), and barcoding adapters 1 μM. The reaction was performed by rotating the whole plate at 37°C for 40 min and stopped by adding 0.5 μL of EDTA 0.5 M to each well. The pooled reaction underwent the same wash procedure as that applied after the RT reaction. After the last round of ligation, the stopped reactions were pooled and washed twice with the same wash buffer by centrifugation (1,000*g*, 1 min). The cells were filtered through an 800-mesh nylon membrane before the first wash and counted after the 2nd wash. Pellets were resuspended with appropriate amounts of the wash buffer and aliquoted (~10,000–20,000 cells in 3 μL per PCR tube) for long-term storage at −80°C.

### Single-tube amplification of cDNA

The cells in 3 μL of low salt buffer were lysed by adding 8 μL of lysis buffer comprising Triton X-100,0.275% and proteinase K 275 μg/ml. The lysis was conducted by vortexing at 1,500 rpm at 55°C for 50 min. Each lysate was added with 2 μL of 10× TdT buffer (Tris-acetate pH 7.9,200 mM, Triton X-100 1%), 2 μL of dATP (20 mM), 2 μL of CoCl2 (7.5 mM), 0.2 μL of PMSF (50 mM in DMSO), homemade (900 ng) or commercial TdT (amounts specified in S2 Table, 2 μL were sufficient for 10,000 cells) and water to achieve a final volume of 20 μL for polydeoxyadenylation at 37°C for 60 min and 42°C for 10 min, followed by heat inactivation at 75°C for 20 min. After adding 1 μL of EDTA (16 mM), the reactions were added to a 58 μL mixture containing 16 μL of KAPA HiFi HF buffer (5×), 1.6 μL of dNTP mixture (10 mM each), and 8 μL of supT25*V (2 μM). The mixture was split into 2 equivalent 39 μL portions, heated at 94°C for 1 min, and held at 45°C for adding 1 μL of KAPA HiFi to each reaction. After adding the polymerase, second-strand synthesis was performed with 54 cycles of 0.5°C increase for 20 s each cycle, followed by 72°C for 10 min and 4°C indefinitely. The reactions were pooled and added to an 80 μL mixture containing 16 μL of KAPA HiFi HF buffer (5×), 1.6 μL of dNTP (10 mM each), 16 μL of suppressive primer (5 μM), 8 μL of DMSO, and 2 μL of KAPA HiFi. The reaction was heated to 98°C for 1 min, followed by 12 cycles of 98°C for 10 s, 68°C for 20 s, and 72°C for 4 min. The PCR reaction was added with EDTA to a final concentration of 3 mM to stop the enzymatic activity.

### Purification of PCR products

To purify the preamplified PCR product, the PCR reactions were supplemented with 1 μL of carboxylated paramagnetic beads and a PEG/NaCl solution composed of Tris-HCl pH 8 (10 mM), PEG8000 (20%), NaCl (2.5M), EDTA (1 mM), and Tween 20 (0.05%) to achieve a final PEG concentration of 7%. The mixture was incubated for 10 min and then placed on a magnetic stand for 5 min. After removing the supernatant, the beads were washed twice with 500 μL of freshly prepared ethanol-water (80%) by inverting the tubes horizontally 10 times on the magnetic stand before removing the supernatant. The beads were left on the magnetic stand for 5 min to evaporate the remaining ethanol, followed by elution with 8 μL of loTE for 10 min. A similar procedure was used to perform size selection on the tagmented cDNA, except that a PEG concentration of 5.5% was used to remove high molecular-weight DNA first. The supernatant was removed from DNA-coated beads and supplemented with 1 μL of paramagnetic beads and additional PEG/NaCl solution to a final PEG concentration of 7% before incubation, washes, and finally elution using 8 μL of loTE containing Tween 20 (0.1%).

### Tn5 tagmentation of preamplified DNA

The eluted DNA from the first-round PEG/NaCl purification was assayed using SYBR Green dye with a calibration curve produced by plasmid DNA of known concentration. Fifty ng of the preamplified DNA and 50 ng of the carrier plasmid were tagmented with 0.5 μL of Tn5 (2 μM) loaded with an annealed adapter containing an i5 PCR handle (S1 Table) in 10 μL of tagmentation buffer (Tris-HCl pH 8.5 10 mM, MgCl_2_ 5 mM, DMF 10%) at 55°C for 30 min. The inactivation of Tn5 was performed by adding 2.5 μL of a quench buffer (SDS 0.25%, EDTA 21 mM) and heating at 55°C for an additional 10 min. The optimal Tn5 amount was determined by pilot tagmentation with 100 ng of plasmid DNA, followed by inactivation and gel electrophoresis to target an intensity peak at around 800 bp. One μL of the quenched reaction was used to determine the optimal cycle number in 10 μL of qPCR reactions composed of KAPA HiFi HF buffer (1×), dNTP (0.2 mM each), i5 primer (200 nM), supAGC primer (200 nM), Triton X-100 (0.1%), SYBR Green (1/30,000 v/v), ROX (500 nM), and 0.125 μL of KAPA HiFi. The thermocycling includes initial denaturation at 98°C for 1 min, followed by 20 cycles of 98°C for 10 s, 63°C for 15 s, and 72°C for 30 s. The cycle number that yielded half of maximum fluorescence was used to amplify the rest of the tagmented samples with identical PCR conditions, replacing SYBR Green, ROX, supAGC, and i5 with DMSO (5% in the reaction), water, P7supAGC, and Nextera i5, respectively. Following adding EDTA (0.5 M) to achieve a final concentration of 3 mM, the PCR product underwent size selection using PEG/NaCl, as described above.

*The raw sequencing reads, processed counts, scripts, and associated datasets to reproduce all sequencing-associated figures are available at GEO and Zenodo (data availability section). Below is the description of the processing.*

### Read processing for gene-count tables

Libraries were sequenced using Illumina HiSeq X or NovaSeq X Plus platforms with the strategies specified in S1B Fig. The output fastq files served as the input for zUMIs with the following custom parameters: ‘additional_STAR_params: --outFilterMismatchNoverReadLmax 0.05’, ‘BarcodeBinning: 0’, ‘strand: 1’, ‘primaryHit: no’, and ‘twoPass: no’. The first 50 base pairs of the Read-1 sequencing outputs served as the cDNA input for the alignment using STAR [80]. The reference genome fasta and gtf files for human, mouse, and maize were GRCh38, GRCm38, and B73 version 5, respectively. The ‘downsampling’ parameter of zUMIs was used to obtain subsampled reads of various depths from each barcode. The intronic, exonic, and total reads were derived from the respective ‘intron’, ‘exon’, and ‘inex’ matrices in either the ‘readcount’ or ‘umicount’ output of zUMIs.

### The reference datasets used for benchmarking

For the benchmark using cultured cells, the reference datasets of 10× Chromium (500 1:1 Mixture of Human HEK293T and Mouse NIH3T3 cells, 3′ LT v3.1, Chromium X and Chromium Controller), Quartz-Seq2 (SRR9621763-6) [5], VASA-seq (SRR14783059) [4], sci-RNA-seq (SRR5509657-754) [40], PIP-seq v4 (https://fbs-public.s3.us-east-2.amazonaws.com/public-datasets/mixed_human_mouse_v4/fastqs.tar.gz, random subset of 1,500 cells for the benchmark), and SPLiT-seq (SRR6750057) [10] were used for the benchmark. The respective barcodes, UMIs, and the first 50-bp cDNA reads were processed identically as the USPPAR dataset (SampleID1) to derive count tables using the identical input parameters, including the downsampled counts for Figs 3E, 3F and S4B–S4D. For Fig 3B, the count tables of the reference and USPPAR datasets were merged using the ‘concatenation’ function (join = ‘outer’) of the Python package ‘SCANPY’ [81]. The same tool was used to subset the merged dataset, keeping barcodes containing more than 500 genes and, subsequently, retaining the top 2,500 highly variable genes using the default parameters. Further, the subset dataset served as the input for the Python package ‘scVI’ [28] (default parameters except batch_key = ‘technologies’, max_epochs = 600, early_stopping = True, train_size = 0.9) to model 2-dimensional (2D) UMAP embedding (min_dist = 0.3), Leiden identities (resolution = 0.1), and normalized gene expression. For the sample correlation in Figs 3C and S4A, the human cells (downsampled 20,000 reads) from Leiden0 and 2 clusters were summed based on both sequencing platforms and Leiden clustering. The sums were subjected to regularized log transformation using the ‘rlog’ function of the R package ‘DESeq2’ [82]. The transformed values were used to derive the PCA plot (Fig 3C) and the correlation heatmap [83] (S4A Fig). The Leiden0, representing HEK293 cells, in Fig 3B, was used for further benchmarks [39] for UMIs and genes detected in Figs 3E, S4B, and S4C. The intronic percentages in Fig 3D were acquired by dividing the intronic counts by the sum of intronic and exonic ones for each HEK293 cell in Leiden0. For the cumulative gene counts in S4D Fig, the Leiden0 cells were randomly sampled increasingly from 1 to 50 cells per platform. Each sampling was repeated 50 times, and the average numbers of genes detected were plotted. The estimation of dropout probabilities in Fig 3F was performed using the P package ‘SCDE’ [39,42]. Specifically, 50 subsampled Leiden0 HEK293 cells from each of the 7 datasets had their counts cleaned (min.lib.size = 500, min.reads = 1, min.detected = 1) to model platform-dependent dropout probabilities by expression magnitudes.

For the benchmark using PBMCs, the reference datasets of 10× Chromium (GEM-X v4, 20k Human PBMCs Multiplex Sample (Donors 1-4)), Quartz-Seq2 (SRR9621763-6) [5], SPLiT-seq (Evercode v3, SRR33305102), and PIP-seq V (SRR33305141) [43] were used. The processing to derive count tables and downsampled counts was identical to that used for HEK293 above. After retaining barcodes with 500–10,000 detected genes, 8,176 barcodes were randomly subsampled from each dataset, except for Quartz-Seq2, for which only 1,006 barcodes were available due to its low cell throughput. Next, the top 6,000 highly variable genes were used to generate a 2D UMAP embedding, following the same procedure as for cultured cells above, except with ‘continuous_covariate_keys = [‘n_genes_by_counts’]’, ‘n_layers = 2’, and ‘min_dist = 0.1’. For embeddings derived from the USPPAR dataset alone, the 8,176 cells were modeled using the same approach as the joint model, except that all genes were included, ‘batch_key = ‘batch’’, and ‘min_dist = 0.3’.

### Comparison and plotting of gene detection efficiencies

To compare treatment-dependent gene detection efficiencies of barcodes prepared in the same experiments and libraries shown in S2D, S2I, S2K, S8B, and S8C Figs, the ‘n_genes_by_counts’ output from the QC metrics of SCANPY was used to obtain gene counts per barcode. For comparisons across libraries or techniques (Figs 3E, 3F, 4A, 6C, 6E, 7C, 7G, 7H, 8C, 8D, S2G, S4B–S4D, and S14A), the same number of subsampled reads or UMIs was obtained from the zUMIs output to ensure a fair comparison. This approach eliminated counting artifacts that could be introduced by different read depths. The split-violin and violin plots were plotted using the R packages ‘ggplot2′ [84], ‘ggpubr’ [85], and ‘introdataviz’ [86]. Statistical comparisons of medians were performed using the Wilcoxon signed-rank test from the ‘ggpubr’ package [85].

### Mixed-species plotting

For Fig 3A, barcodes were classified as ‘human’ or ‘mouse’ if the UMI counts for human or mouse genes constituted more than 90% of the total. Otherwise, they were categorized as ‘multiplet’. A similar rule was applied to S15A Fig, with the analysis including human, mouse, and maize species.

### Reveal and correct the frameshift barcodes

FastQC [87] was used to derive the per-base sequence contents of A, T, C, and G from the Read-2 sequences in the SampleID 4 (mixed spleen and liver). The analysis was performed using either the sequences from the whole library (All) or those belonging to the Leiden6 (Leiden 6) barcodes. The Leiden 6-specific Read-2 and Read-1 sequences were obtained using the ‘grep’ and ‘pair’ functions, respectively, of the Python package ‘SeqKit’ [88]. The Read-2 sequences containing the frameshifted Leiden 6 barcodes were used to acquire the correct barcodes using Cutadapt [89] to refer to the contributing barcodes in S8E and S8F fig.

### Tests for differential expression

The R package ‘glmGamPoi’ [90] was used to test for differential gene expression between samples. The cutoffs of adjusted p-values and log2(fold changes) in each comparison were specified in the respective figures. The values beyond the plottable range were capped. To identify genes belonging to histones or snoRNAs, as shown in S2L Fig, histone-coding genes were retrieved from the HUGO database, while snoRNA genes were identified using the gene_biotype == ‘snoRNA’ annotation in the GTF files of GRCh38 and GRCm38. To reveal the GO terms differentially enriched in the two monocytic subclusters shown in S10D Fig, the output of the differential expression analysis (adjusted *p*-value < 0.05 and log2(fold change) > 1) served as input for the ‘enrichGO(ont = “BP”, pAdjustMethod = “BH”, pvalueCutoff = 0.01, qvalueCutoff = 0.05, readable = TRUE)’ function of the R package ‘clusterProfiler’ [91]. The top 12 enriched terms with the lowest *q*-values (*p*.adjust < 0.05, *q*-value < 0.05) were selected for the dot plot in S10E Fig.

### Modeling cell connectivity and gene expression with scVI

For mouse organs, the mouse cells were defined by the percentages of mouse UMI counts higher than 90%. The count matrices were filtered to retain only protein-coding genes with gene IDs starting with ‘ENSM’. The nuclear datasets from individual and pooled mouse organs (cells with >200 detected genes; 200–2,500 genes for the CuC liver dataset) were modeled with the parameters used in Fig 3B except for genes with summed counts of more than 3 across all cells being kept, batch_key = [‘library preparation batches’, ‘fixatives’] for the CuC liver dataset and batch_key = [‘library preparation batches’] for all other datasets, and continuous_covariate_keys = [‘n_genes_by_counts’]. The joint modeling of mouse pancreatic nuclei from pH3 and CuC lysis buffers was the same as modeling each buffer separately, except that the batch key was set to [‘lysis’] instead of [‘batch’]. The models were used to infer UMAP embeddings (min_dist = 0.3 for liver, pancreas, and spleen with pH3 buffer and spleen with CuC buffer; 0.1 for the others), Leiden identities (resolution: pancreas = 0.25; liver = 0.3; spleen = 0.3), and normalized gene expressions. The cell-type markers were mostly chosen from references [23,54,92]. For the integration of all mouse nuclei across organs (pancreas, liver, and spleen) and lysis buffers (pH3 and CuC) in S13 Fig, the same modeling strategy was used, with the addition of categorical_covariate_keys = [‘sources of mouse organs’, ‘lysis buffer used’, ‘batch’] during modeling. The batch-corrected, normalized expression levels from scVI modeling were used to plot gene expression on UMAPs in Figs 6D, S5A, S5B, S6F, S6G, S7B, S7C, S8B, S10B, S10C, S11A, S11B, S12B, S12C, S13, S14B, and S15C. Marker expressions per Leiden cluster in S5A, S6F, S7B, S10B, S11A, and S12B Figs were plotted using the pl.stacked_violin(standard_scale=’var’, layer=’scVI normalized expression values’) function in SCANPY.

The dataset from purified whole-cell splenocytes, generated using the 10× Chromium v3 (10k_Mouse_Splenocytes_5p_gemx_Multiplex), was used for comparison with nuclear data derived from one-pot lysis using USPPAR. In each dataset, only barcodes containing 200–1,500 genes were included for the modeling. The top 2,000 highly variable genes, identified using ‘pp.highly_variable_genes()’ in SCANPY, were retained for integration with scVI using the same parameters as for mouse organs (batch_key = ‘source of data’), except with ‘n_layers = 2’. The UMAP inference and the derivation of batch-corrected normalized expression values were identical to those used for the mouse organs above. A lower resolution (0.2) was applied to identify the main cell types in Figs 6D and S10F, while a higher resolution (0.9) was used to reveal subclusters within the monocytic cluster in S10D Fig.

For the benchmarking using mouse liver, we used a randomly selected subset of datasets from the Mouse Liver Cell Atlas [93] (SRR17374901–5 and 12, generated with 10× Chromium v2 and v3) and from PanSci (20230208_EXP101_08_S6, 67_S65, 20230322_EXP101_035_S131, 20230323_EXP101_067_S67, and 003_S3). For PanSci, shortT indices were mapped to the corresponding randomN indices for the same cells before generating count tables with zUMIs. Count tables were combined and barcodes filtered (minimum mouse protein-coding genes = 3). From each source (USPPAR_CuC, USPPAR_pH3, LCA, and PanSci), 12,646 barcodes were subsampled for joint modeling. The top 4,000 highly variable genes were used, with categorical covariates set to the four sources and the fixatives used in USPPAR_CuC, and continuous covariates set to ‘n_genes_by_counts’. Models were trained with three layers to infer 2D UMAP embeddings (min_dist = 0.1).

For the maize roots and shoots (seedlings), the maize cells were defined by the percentages of mouse UMI counts higher than 90%. The count matrices were filtered to retain only genes with IDs starting with ‘Zm00’. For roots, barcodes with more than 120 detected genes were used for scVI modeling with the same parameters as the mouse datasets, except for continuous_covariate_keys=’None’. For shoots, barcodes with more than 400 detected genes were modeled with the same parameters as the mouse datasets, with the following adjustments: (i) For S2H Fig (USPPAR1 dataset in S15B Fig), no batch correction between homebrew and commercial TdT was applied, to demonstrate the absence of batch artifacts in the UMAP plots; (ii) For joint USPPAR1 and USPPAR2 analysis in S15C Fig, ‘batch_key = [‘source of datasets (USPPAR1 & 2)’]’; (iii) For Figs 8E and S15B (joint REF, USPPAR1, and USPPAR2), genes detected in > 1 barcode were included, and models were trained with ‘n_layers = 2’ and ‘categorical_covariate_keys = [‘batch’, ‘source of datasets (10×_v3, USPPAR1 & 2)’]’. These models were used to infer UMAP embeddings (min_dist = 0.1), Leiden clustering identities (resolution: root = 0.6; shoot = 3 for cell-type annotation), and normalized gene expressions.

### Cell type annotation of PBMCs, mouse, and maize shoot nuclei

For annotating the shoot cells in the USPPAR dataset using the reference dataset [27], the reference shoot dataset was modeled first using the same approach as for the shoot USPPAR dataset. Leiden clusters at high resolution (resolution = 2) were used to annotate the unannotated cells based on the average expression values of the remaining annotated cells in the reference dataset, using the R package ‘clustifyr’ [30]. Next, the joint model in Figs 8E/S15B was used to perform Leiden clustering (resolution = 3). These Leiden clusters were used to annotate barcodes belonging to the USPPAR dataset based on the average expression values of cell types in the reference dataset.

For PBMC annotation, the same procedure and parameters were applied to both the joint model (resolution = 2) and the USPPAR model (resolution = 4), using the 11,990-cell scVI internal PBMC dataset as the reference. For cross-species annotation of the CuC, pH3, and joint pancreatic datasets, we used the human pancreatic reference (Tosti and colleagues [25]), applying the same procedure and tool as above(resolution = 3). For the CuC and joint liver datasets, we used the PanSci liver reference dataset and selected the top 4,000 highly variable genes(resolution = 3).

### Statistical tests

The statistical tests for each figure were specified in the respective legends or the associated methods above.

## Supporting information

S1 Fig The stepwise sequence modifications (A) to generate the final library structure (B) shown in Fig 1B. The adapter 1.5 is omitted for 3-round barcoding. The splinters used for ligation are underlined above and beneath the final library structure for 3- and 4-round barcoding, respectively, in the final library **(B)**. The cDNA sequence was derived from 50-bp sequences obtained using the Read-1 sequencing primer. The barcodes and unique molecular identifiers (UMIs) were derived from Read-2 (3-round) or Read-2 and Read-3 (4-round) sequencing results. The 8-bp index reads from the Index-2 sequencing primer were used to demultiplex multiple libraries. Because the NovaSeq X Plus does not support custom Read-2 primers, the final library PCR (step 9a in A) used primer P7NextR2supAGC (S1 Table), which contains the full Nextera Read-2 sequence (S1B Fig, blue sequence in 3 Rounds-A), enabling sequencing with Illumina’s default primers.
(TIF)

S2 Fig Quality control of homebrew enzymes and parameter testing. **(A)** Silver staining of TBE-PAGE (20%) validating the activities of homebrew T4 polynucleotide kinase (PNK) and T4 DNA ligase. 1 nmole of BC1 (TACCCTACTACTCTCACCACCATCTCTACCACTTC) was treated with 50 ng of homebrew PNK in 20 μL reaction at 37°C for 2 hours and heated at 65°C for 20 min. 2.5 pmole of the phosphorylated BC1 (pBC1), 5 pmole of the annealed BC2 adapter (5.625 pmole TATAGAATTCGCGGCCGCTCGCGATAGCNNNNNNNNATCCTCCTACTCTCACCAA annealed with 5 pmole GTAGGGTATTGGTGAGAGTAGGAGGA) were incubated in 10 μL of ligation reaction mixture composed of Tris-HCl pH 7.5 (70 mM), MgCl_2_ (10 mM), DTT (5 mM), ATP (1 mM), PEG8000 (7.5%), and homebrew T4 DNA ligase (80 ng) at 37°C for 1 hour. Five μL of the reaction was used for electrophoresis. Arrowhead indicates that pBC1 (arrow) was almost completely converted to the BC2-to-BC1 ligation product. Lanes 1−3 served as the non-ligation controls. **(B)** Silver staining of TBE-PAGE (20%) to verify the adapters used for 3-round split-pool barcoding based on ligation. The ligation was performed with combinations of oligos pBC0 (phosphorylated BC0) and annealed adapters pBC1 (equimolar annealed phosphorylated BC1 with Splinter-1) and BC2 (equimolar annealed BC2 with Splinter-2) in 10 μL of reaction mixtures composed of Tris-HCl pH 7.5 (70 mM), MgCl_2_ (10 mM), DTT (5 mM), ATP (1 mM), rat RNase inhibitor (10 ng), PVA (0.1%), and homebrew T4 DNA ligase (160 ng) at 25°C for 1 hour. 2.5 pmole of donors (D) and 10 pmole of acceptors (A) were used in each reaction to demonstrate a complete upshift of the donor pBC1 in lane 8. 4 μL of the reaction was used for electrophoresis. Asterisks denote the location of the expected ligation products, and the arrow denotes the unligated pBC1 that barely remained after the reaction in lane 8. **(C)** RT-qPCR to benchmark the homebrew thermostable M-MuLV reverse transcriptase (M5) against the commercial ones. 500-cell lysates of hPSCs were used for RT reaction (20 μL), and half of the cDNA was used for qPCR in triplicate. The Y-axis shows the relative enrichment of cDNA with reverse transcriptases against the no-enzyme control (No RT) [94]. Primers targeting PSMB4 exon and VEGFA promoter regions served to amplify the cDNA and reference genomic DNA, respectively. MMLV, SSII, and M5 denote commercial wild-type M-MuLV (0.25 μL), SuperScript II (0.25 μL), and homebrew thermostable reverse transcriptase (50 ng), respectively. **(D)** Violin plot with jittered points for genes detected per barcode to compare sensitivity with different enzymes, incubation temperatures, and random primers used during RT. HEK293T cells were processed under eight RT conditions (1,000 cells per 20 µL reaction), with each condition performed in separate PCR strips before pooling for washes and ligation during the Barcoding stage. Conditions varied by enzyme (M5, 5 ng/20 μL, or Maxima H^−^ at different U), incubation temperature (42: 42°C for 40 min; 2,542: 25°C for 10 min then 42°C for 40 min; 4,250: 42°C for 20 min then 50°C for 20 min), and primer type (N6, random hexamer; N9, random nonamer). Cells with 1,000–8,000 detected genes were retained for analysis. **(E)** Fluorescence assay to show the inhibition of the RNase A activity by homemade and commercial recombinant RNase inhibitors. The releases of quenched FAM reporter (50 nM) by the RNase A (RA, 0.5 ng) were studied in the presence of commercial SUPERase•In RNase inhibitor (0.5 μL, S.I. in lane 2) or homebrew recombinant rat RNase inhibitor (100 and 200 ng, rRi in lanes 3 and 4, respectively). These experiments were conducted in 50 μL of reaction buffer composed of Tris-HCl pH 7.5 (50 mM), NaCl (35 mM), KCl (10 mM), MgCl_2_ (1.5mM), CaCl_2_ (0.5mM), and Triton X-100 (0.05%). The fluorescence collection of triplicates was performed after 30 min of incubation at 37°C. Lanes 1 (in the circle) and 5 (in the square) served as RNase A (−) negative and RNase inhibitor (−) positive controls, respectively. **** denotes *p* < 0.0001 based on one-way ANOVA with Dunnett post hoc tests using lanes 5 as the reference. Individual replicate values are shown as black filled circles, and means are indicated by blue horizontal lines in scatterplots (C) and (E). **(F)** Silver staining of TBE-PAGE (20%) to demonstrate the activities of homebrew terminal deoxynucleotidyl transferase (TdT) against the commercial one. 3′ recessed dsDNA was prepared by annealing 25 μM each of TACCCTACTACTCTCACCACCATCTCTACCACTTC and GTGATGGTTAGTGAGGAAGTGGTAGAGATGGT (hybridized regions underlined) in Tris-HCl pH 8 (25 mM), NaCl (12.5 mM), and EDTA (0.25 mM). 2.5 pmol of the 3′-recessed dsDNA was used as the tailing substrate for the commercial (C, 1.5 μL) and homebrew (H, 1,300 ng) TdT in 15 μL of TdT reaction containing Triton X-100 (0.1%), Tris-acetate pH 7.9 (20 mM), dATP (2 mM), CoCl_2_ (0.75 mM), PMSF (final 0.5 mM), and commercial or homemade TdT at 37°C for 60 min followed by 75°C for 20 min. Half of the reaction was used for electrophoresis. Lysate refers to the use of a 5,000-cell lysate to demonstrate sufficient tailing, even in the presence of additional background DNA ends. This lysate was derived from a 5,000-cell subsample of 250,000 human cells that underwent 3-round split-pool barcoding using the same procedure as described in Fig 3. The cells were lysed in Triton X-100 (0.2%) and proteinase K (200 μg/mL). Lane 1 is the lysate- and enzyme-free control to locate the untailed substrates (arrows). **(G)** Line-and-point plot showing the gene-detection efficiencies of maize root (Root) and shoot (Shoot) nuclei by single-nuclei RNA-seq, using commercial TdT (cTdT, dark red-brown) and homebrew TdT (hTdT, blue). Median numbers of reads (solid), UMIs (dashed), and genes (dotted) per nucleus are plotted at down-sampled sequencing depths. Barcodes containing more than 120 genes (Root) or more than 400 genes (Shoot) were retained for the analysis. **(H)** UMAPs of shoot nuclei (genes detected > 400) from libraries prepared using commercial TdT (cTdT, 4,522 cells) or homemade TdT (hTdT, 5,459 cells). The same nuclei from the USPPAR dataset in Fig 8E were modeled without batch information to reflect batch artifacts introduced by libraries prepared using different enzymatic sources. **(I)** Split-violin plot for genes detected per cell for HEK293T cells directly fixed in methanol (−Glyoxal) or those undergone additional fixation in 3% glyoxal (+Glyoxal). For methanol fixation alone, the dissociated cells were resuspended with 10 μL of PBS and fixed immediately by adding 200 μL of −20°C methanol. For extra fixation with glyoxal, the cells in 10 μL PBS were fixed by adding 100 μL of 3% glyoxal, pH 4 [95], rotated at 4°C for 15 min, and quenched with 1,000 μL of PBS containing PVA (0.1%) and Tris-HCl pH 7.5 (250 mM) before centrifugation and fixation as the -Glyoxal group. **(J)** Stacked bar plots showing the cumulative UMI counts for different RNA biotypes from pseudobulked reads in the +N9 and −N9 groups (608 cells each) in (K). **(K)** Split-violin plot showing the total number of genes detected per cell (left) and the number of genes from different RNA species (right) when T16V alone (−N9) or T16V plus random nonamer (+N9) was used as the RT primer. The cells, in the experiment in Fig 3 (USPPAR), had cDNAs from the first 48 wells barcoded with T16V alone (−N9) and the other 48 wells barcoded with T16V+N9 (+N9). Different RNA species were classified by biotype according to Ensembl GRCh38.99. Only RNAs with median counts > 5 in either the +N9 or −N9 group were included for plotting. Diamonds denote the medians of each group, and the p-values are obtained with the Wilcoxon rank-sum test in (D), (I), and (K), and Bonferroni-corrected post hoc adjustments (*q* values) were applied for comparisons in (D). **(L)** Volcano plot showing differentially expressed genes in HEK293T cells prepared with different primer compositions during RT in the experiment depicted in S2K Fig. The plot displays log2(fold change) on the x-axis and −log10(adjusted *p*-values) on the y-axis. Genes are colored green (adjusted *p* values < 0.05, log2(fold change) > 1), pink (adjusted *p* values < 0.05, log2(fold change) <−1) or gray. The enlarged points denote histone- (left, HUGO) or snoRNA- (right, biotype ‘snoRNA’ in Ensembl GRCh38.99) coding genes, most of them being non-polyadenylated [96]. The blue ratios represent the intersected genes (colored + enlarged) over all differentially expressed ones (colored) in respective categories. **(M)** Photographs of columns assembled with 800-mesh nylon membranes to filter clumps/multiplets, minimizing sample loss compared to regular strainers. The silica-membrane containing spin columns (e.g., Miniprep columns) were disassembled to obtain the column shaft (a) and the holding rings (b). 800-mesh nylon membranes were cut into 7 mm-diameter disks and held between 2 holding rings in the column shown in (c). Dissociated tissues and barcoded cells/nuclei could be filtered through the membrane using low-speed centrifugation or dripping by gravity. The uncropped images of the denaturing PAGE for (A), (B), and (F) can be found in S1 Raw Images. Numerical data for (C) and (E) are available in S1 Data. Raw count data are available at GEO (GSE256014) and code for analysis and plotting at Zenodo (https://zenodo.org/records/13927875) for panels (D), (G)–(L).
(TIF)

S3 Fig The effects of deoxynucleotide types (dNTP), buffers (Buffer), a reducing agent (DTT), and ion types/concentrations (M^2+^/Concentration) on tailing the recessed-end DNA substrate. **(A)** Silver staining of denaturing TBE-PAGE (15%) showing differential polydeoxyadenylation with different concentrations of Mg²⁺ and Co²⁺. The annealed 3′-recessed-end duplexes (2.5 pmol) were incubated in 10 μL reaction mixtures containing dATP (2 mM), TdT (0.5 μL), and various concentrations of MgCl₂ or CoCl₂ in 20 mM Tris-acetate pH 7.9, and 0.1% Triton X-100. The reactions were performed at 37°C for 60 min, followed by heat-inactivation at 75°C for 20 min. Subsequently, half of each reaction was loaded for electrophoresis. Arrows indicate the unextended oligos. **(B)** Silver-stained 20% denaturing TBE-PAGE showing effects of additional buffer recipes. 5 pmole of the substrate was incubated in 10 μL of reaction containing HEPES (H) or Tris-acetate (T) pH 7.9 (20 mM), dATP (dA) or dGTP (dG) (2 mM), CoCl_2_ (Co) or MnCl_2_ (Mn) (0.75 or 2 mM), DTT (0 or 0.2 mM), and TdT (0 or 0.5 μL) at 37°C for 60 min followed by 75°C for 20 min before loading half of the samples for electrophoresis. The 3′ recessed-end dsDNA substrate was prepared as in Fig 2D. Arrows denote the untailed oligo substrates. The uncropped images of the denaturing PAGE can be found in S1 Raw Images.
(TIF)

S4 Fig Additional benchmarking of scRNA-seq using libraries prepared with the optimized TdT reaction system. **(A)** The heatmap for the Pearson correlations of the pseudobulk expression values in Fig 3C. The UMI counts from subsampled (20,000) reads of Leiden0 (HEK293) and Leiden2 (HeLa S3) cells in Fig 3B were regularized and log-transformed prior to Pearson correlation calculation and plotting. Blue open and solid circles denote USPPAR with (+N9) and without (−N9) random nonamers during RT. **(B, C)** Medians of detected UMIs (B) and genes (C) at different subsampled read depths per HEK293 cell across technologies. The reads from cells in Leiden 0 in Fig 3B, representing the 7 platforms, are included in the analysis. **(D)** Cumulative numbers of genes detected in HEK293 cells across technologies. The cells in Fig 3E are randomly sampled increasingly from 1 to 50 cells per dataset. Each sampling was repeated 50 times, and the average numbers of genes detected are plotted. Raw count data are available at GEO (GSE256014) and code for analysis and plotting at Zenodo (https://zenodo.org/records/13927875).
(TIF)

S5 Fig Normalized gene expression (A, B) and cell-type compositions by treatments (C, D) in PBMC scRNA-seq data. (A) Stacked violin plot showing scaled, normalized expression of marker genes for each cell type in Fig 4E (Independent CellType). The color scale ranges from white (lowest value) to dark blue (highest median expression). (B) UMAPs of 8,176 PBMCs in Fig 4E, colored by cell-type annotation (CellType) and the expression of marker genes for T (CD3E, CD3G), CD4 T (CD4), CD8 T (CD8A, CD8B), B (CD19, MS4A1), NK (NCAM1, KLRB1, NKG7), plasmacytoid dendritic (LILRA4), monocytic (CD14, CD68, FCGR3A), and dendritic (CD1C) cell types. The color scale for gene expression ranges from dark purple (lowest normalized expression) to yellow (highest). (C) UMAPs of 8,176 USPPAR PBMCs from Fig 4E (Independent CellType), split by ABF treatment (ABF) or methanol only (MeOH). Treatment information was not provided during modeling to demonstrate unsupervised integration of the two conditions. (D) Stacked bar plots showing the percentages of annotated cell types in the ABF and MeOH treatment groups in (C). No significant differences in cell counts across conditions by chi-squared test with FDR-BH(all *q* > 0.05). Raw count data are available at GEO (GSE256014) and code for analysis and plotting at Zenodo (https://zenodo.org/records/13927875).
(TIF)

S6 Fig Nuclei extracted using a pH3 lysis buffer enabled single-nucleus RNA-seq (snRNA-seq) from the murine pancreas. **(A)** Photographs of the process for making folded polyimide (PI) films used for pulverizing tissue at liquid nitrogen temperature. The PI films (0.1 mm thick) were cut into 8-cm squares (a), rolled (b), and folded into a tube (c) with one side sealed using tape (d). After being kept at liquid nitrogen temperature for more than 10 min, a frozen tissue fragment was placed into the tube and crushed on a liquid nitrogen-frozen steel plate using an aluminum block (Pulverization step in (B)). The pulverized powder was immediately lysed using lysis buffers, followed by pestling (for mouse tissues) or Dounce homogenization (for maize tissues) to achieve the immediate quenching of nuclease activities (Lysis step in (B)). **(B)** The dissociation procedure for extracting nuclei using the pH3 buffer. Pulv.: Pulverization; Purif.: Purification. **(C)** Scatter plot showing the inhibition of green fluorophore release by pancreatic nucleases using low-pH buffers. The nuclease-cleavable oligo (50 nM) was prepared in 25 μL of reactions. The reactions were supplemented with various buffers (-: 10 mM Tris-Cl, pH 8.0; 2.4, 3, 3.5, 4: 25 mM sodium citrate at the respective pH levels) in the presence or absence of 250 mM sucrose or 4 μL of pancreatic lysate. After incubating at ambient temperature for 60 min, the release of the fluorophore due to cleavage was quantified using the green fluorescent channel. **** denotes *p* < 0.0001 based on one-way ANOVA with Dunnett’s post hoc tests using lane 7 (in square) as the reference. Individual replicate values are shown as black filled circles, and means are indicated by red horizontal lines. Lane 2 (in circle): additive-free control. **(D)** Fluorescence DNA staining of nuclei extracted using pH3 or Cu²⁺-chelator complex (CuC) lysis buffer. The three organs underwent the Pulverization, Lysis, and Purification steps as shown in S6B Fig (pH3) or Fig 6A (CuC). Nuclei at the 30%–60% interface were collected and washed three times with the respective wash buffers. The resuspended nuclei were washed again and stained with Hoechst 33258 in nuclear staining buffer for microscopic imaging. Scale bar: 50 µm. **(E)** UMAPs of 11,630 murine pancreatic nuclei (genes detected > 200), colored according to Leiden labels. Cells are colored based on Leiden clusters derived from all reads. The inferred cell types, identified according to marker expressions in (F, G), are specified after the respective Leiden labels. **(F)** Stacked violin plot showing the scaled normalized expression of marker genes in pancreatic Leiden clusters in (E). The color scale ranges from white (lowest value) to dark blue (highest median expression). **(G)** UMAPs of 11,630 murine pancreatic nuclei (genes detected > 200), colored according to Leiden clustering (Leiden) and the expression levels of ductal (Krt19, Krt8, Epcam, and Cdh1), acinar (Pnlip, Try5, Ctrc, Cpa1, Ptf1a, Sox9, and Prom1), islet (Pax6), erythroid (Gata1 and Gypa), mesenchymal (Col1a1 and Dcn), endothelial (Cdh5), mesothelial (Msln and Upk1b), and hematopoietic (Ptprc, Mpo, Cd3d, and Cd79b) markers. The color scale for gene expression ranges from dark purple (lowest normalized expression) to yellow (highest). Mes: mesenchymal; Endo: endothelial. Numerical data for (C) are available in S1 Data. Raw count data are available at GEO (GSE256014) and code for analysis and plotting at Zenodo (https://zenodo.org/records/13927875) for panels (E)–(G).
(TIF)

S7 Fig The pH3 lysis buffer also enabled snRNA-seq from the murine liver. **(A)** UMAPs of 17,487 murine hepatic nuclei (genes detected > 200), based on all reads (Exon + Intron) or intronic reads alone (Intron Only). Cells are colored based on Leiden clusters derived from all reads. The inferred cell types, identified according to marker expressions in (B, C), are specified after the respective Leiden labels. **(B)** Stacked violin plot showing the scaled normalized expression of marker genes in hepatic Leiden clusters in (A). The color scale ranges from white (lowest value) to dark blue (highest median expression). **(C)** UMAPs of 17,487 murine hepatic nuclei (genes detected > 200) colored according to Leiden clustering (Leiden) and the expression levels of hepatic (G6pc, Hnf4a, Alb, Cyp2e1, Afp, and Lgr5), stellate (Reln and Dcn), ductal (Spp1, Krt19, and Epcam), Kupffer (Adgre1 and Spic), mesenchymal (Col1a1 and Pdgfra and Eln), mesothelial (Msln and Upk1b), endothelial (Kdr and Cdh5), and hematopoietic (Mpo, Cd3d, and Cd79b) markers. The color scale for gene expression ranges from dark purple (lowest normalized expression) to yellow (highest). Mes: mesenchymal; Endo: endothelial. Raw count data are available at GEO (GSE256014) and code for analysis and plotting at Zenodo (https://zenodo.org/records/13927875).
(TIF)

S8 Fig Nuclei extracted with the pH3 lysis system showed reduced lymphocyte representation in the spleen, with a small portion showing erroneous cell identities due to truncated reads. **(A)** Integrated UMAP of the combined liver (17,487 cells) and spleen (1,607 cells) prepared using the pH3 buffer. Colors represent the Leiden clusters of the barcodes belonging to the liver, while black denotes those belonging to the spleen. **(B)** Integrated UMAPs of splenic nuclei extracted using the pH3 lysis buffer (1,607 cells) and a buffer containing Cu^2+^-citric acid complex (4,455 cells). Clusters of nuclei extracted using the pH3 buffer are colored according to Leiden labels; nuclei extracted with the Cu^2+^-citric acid complex are shown in gray. The percentages of barcodes belonging to each cluster are shown to the left of the respective color labels. The ‘#’ symbols and asterisk denote clusters with lymphocytic and hepatic markers, respectively. Cd19, Cd3e, and Alb: Normalized expression levels of B-lymphocytic, T-lymphocytic, and hepatic markers for all 6,062 splenic cells, with the color scale ranging from dark purple (lowest normalized expression) to yellow (highest). **(C)** Violin and box plots showing the total genes detected per Ptprc+ white blood cells in the liver (A, Leiden 7 and 8) and spleen (B, Leiden 0, 1, 3, 5, 7), prepared with the pH3 buffer and differentially barcoded in the same experiment. The horizontal bars denote the upper-quartile, median, and lower-quartile values descendingly in each box plot. P-values are obtained with the Wilcoxon rank-sum test. **(D)** Per base sequence content of the barcoding reads assigned to the nuclei (gene detected > 200) shown in (A). All: 96,165,705 reads assigned to all 19,094 cells; Leiden 6: the 70,242 reads belonging to the 83 Leiden 6 cells (B, Leiden, asterisks). The pink shade denotes the 9-bp window of 2 possible 8-bp deletions (8-bp Del) by shifting 1 bp. Gray horizontal bars underline the 3 original 4-bp barcode regions. **(E)** Integrated UMAP of (A) showing the location of corrected barcodes contributing to the aberrant Leiden 6 cluster (sky-blue) due to truncation, alongside the uncorrected barcodes assigned to the aberrant Leiden 6 of the spleen (red). The reads from Leiden 6 in (D) were used to extract the corrected barcodes, accounting for the 8-bp deletion. **(F)** Stacked bar plots showing the composition of Leiden clusters: one for the entire hepatic dataset (left; gray, red, and sky-blue cells in (E)) and the other for the cells contributing to the aberrant Leiden 6 cluster (right; sky-blue cells in (E)). The plots are stretched to the same total height to highlight the similarly high percentages of barcodes belonging to hepatocytes (vertical red bars). Mes: mesenchymal; Endo: endothelial; Gran: granulocytic; Dend: dendritic. Raw count data are available at GEO (GSE256014) and code for analysis and plotting at Zenodo (https://zenodo.org/records/13927875).
(TIF)

S9 Fig Additional demonstrations of the use of the Cu²⁺-chelator complex. **(A)** Photograph showing nuclear purification using iodixanol density-gradient centrifugation. Mouse pancreas fragments (10 mg) were pulverized and lysed with 600 μL of lysis buffer (50 mM HEPES pH 7.2, 146 mM NaCl, 1 mM CaCl₂, 21 mM MgCl₂, with 0.1% each of TWEEN-20 and NP-40, and 0.01% digitonin), containing either DEPC (1%) or Cu²⁺-citric acid (CuC, 10 mM). After further grinding with plastic pestles in microtubes, the lysates were filtered through columns containing 800-mesh nylon to remove large tissue clumps. The filtrate was layered over 500 μL of the same lysis buffer, with detergents replaced by 0.05% PVA and iodixanol (30%), and with 20 μL of 60% iodixanol at the bottom. After centrifugation at 2,000*g* for 5 min in a swing-bucket centrifuge, the photograph was taken. Asterisks: lysate-30% iodixanol interface; Arrows: 30%−60% iodixanol interface. **(B)** Fluorescence staining of the nuclei retained at the lysate-30% iodixanol interface, denoted by asterisks in (A). The nuclei from the interface prepared in (A) were withdrawn and washed once with 100 μL of lysis buffer (50 mM HEPES pH 7.2, 146 mM NaCl, 1 mM CaCl₂, 21 mM MgCl₂) containing 10 μg/mL Hoechst 33,258. After incubation at ambient temperature for 5 min and centrifugation at 1,000*g* for 1 min, 80 μL of the supernatant was removed, and the nuclei were resuspended in the residual supernatant for imaging using a fluorescent microscope. Scale bars: 200 μm. **(C)** Scatter plot showing the inhibition of pancreatic nucleases by metal ions. The nuclease-cleavable oligo (50 nM) was prepared in 25 μL of reaction buffer (20 mM HEPES pH 7.2, 146 mM NaCl, 1 mM CaCl₂, 21 mM MgCl₂, with 0.1% each of TWEEN-20 and NP-40, and 0.01% digitonin). It was supplemented with 4 μL of pancreatic lysate (except in the negative control) and the specified additives (CuSO₄ or ZnSO₄ at various concentrations). After incubating at ambient temperature for 30 min, the release of the fluorophore due to cleavage was quantified using the green fluorescent channel. Lane 2 (in square): positive control for reference in one-way ANOVA with Dunnett’s post hoc tests; Lane 1 (in circle): lysate-free negative control. **(D)** Scatter plot showing the effects of different chelators on the Cu²⁺-dependent inhibition of pancreatic nucleases. The same basal reaction buffer as in (C), containing the specified additives (pancreatic lysate, CuSO₄, Citrate, NTA, EDTA). The incubation and quantification were performed identically to (C). Lane 2 (in square): positive control for reference in one-way ANOVA with Dunnett’s post hoc tests; Lane 1 (in circle): lysate-free negative control. Citrate: diluted from a 1 M stock solution of sodium citrate at pH 7; NTA: diluted from a 0.5 M stock solution of sodium NTA at pH 7; EDTA: diluted from a 0.5 M stock solution of sodium EDTA at pH 8. **(E)** Scatter plot showing the effects of different agents on the inhibition of pancreatic nucleases. The nuclease-cleavable oligo (50 nM) was prepared in 25 μL of reaction buffers (H: 20 mM HEPES pH 7.2, 146 mM NaCl, 1 mM CaCl₂, 21 mM MgCl₂, with 0.1% each of TWEEN-20 and NP-40, and 0.01% digitonin; C: 25 mM citric buffer pH 3, 250mM sucrose), supplemented with 4 μL of pancreatic lysate (except in the negative control) and the specified additives (DEPC or CuC at specified concentrations). The incubation and quantification were performed identically to (C). However, in lane 7, labeled ‘2D’ (delayed), CuC (2 mM final concentration) was added after a 30-min incubation period, in contrast to lane 6, where CuC was included from the beginning of the incubation. Lane 2 (in square): positive control for reference in one-way ANOVA with Dunnett’s post hoc tests; Lane 1 (in circle): lysate-free negative control. The fluorescent quantifications in (C), (D), and (E) were diluted to 150 μL with the respective reaction buffers, excluding the substrate oligo, nucleases, and pancreatic lysate, before fluorescence measurements in triplicate. Individual replicate values are shown as black filled circles, and means are indicated by red horizontal lines in scatterplots (C)–(E). **** denotes *p* < 0.0001. **(F)** Electropherograms of RNA fragment analysis from nuclei prepared with pH3 or CuC lysis buffer. The three organs underwent the Pulverization, Lysis, and Purification steps as shown in S6B Fig (pH3) or Fig 6A (CuC and None). Nuclei at the 30%–60% interface were collected, washed four times with the respective wash buffers, and used for RNA extraction. ‘None’ refers to the CuC lysis procedure performed without the Cu²⁺-chelator complex. Pink and blue bars mark the 18S and 28S rRNA regions, and gray zones under the curves indicate fragments >200 bp. Percentages on each plot represent DV200 values. Numerical data for (C)–(F) are available in S1 Data.
(TIF)

S10 Fig Additional data related to the CuC-based splenic dataset. **(A)** Photograph of the PTFE-holding device used for gently washing cells. Dashed lines indicate the locations of cutting 15-ml tubes and vials. The foam liners of the vials were punched with a 5 mm hole using a hollow steel tube. The hydrophilic PTFE membranes (3 μm pore size) were punched out using a hollow steel tube with a 9 mm inner diameter and secured between the foam liner and the vial, with the cap screwed on top to hold the assembly in place. The cells were washed on the PTFE membranes in the vial, with the flow-through collected in the lower half of the cut 15 ml tube. **(B)** Stacked violin plot showing the scaled normalized expression of marker genes in splenic Leiden clusters in Fig 6B. The color scale ranges from white (lowest value) to dark blue (highest median expression). **(C)** UMAPs of 6,062 murine splenic nuclei (genes detected > 200) are colored according to Leiden clustering (Leiden) and the expression levels of various cell markers: B lymphocytic (Cd79b, Pax5), NK (Ncr1), T lymphocytic (Cd4, Cd8a), megakaryocytic (Pf4), erythroid (Tfrc), dendritic (Clec9a, Clec10a), neutrophilic (Csf3r), mesenchymal (Cal1a1), endothelial (Cdh5), monocytic (Cd5l, Csf1r), plasmacytic (Jchain, Xbp1), and hepatic (G6pc). The dark purple color denotes the lowest normalized expression value, and yellow the highest. **(D)** Integrated UMAP highlighting monocytic subclusters (asterisk in Fig 6D). Dark green: monocytic subset A absent in the USPPAR dataset. Light green: monocytic subset B present in both USPPAR and 10× datasets. Only barcodes belonging to the 10× dataset are colored. The cluster was identified using a higher resolution (0.9) during the inference of Leiden clustering in the same model used for Fig 6D. **(E)** Dot plot showing the biological-process GO terms enriched in the dark green subset in (D). Genes differentially expressed between the two clusters (adjusted *p*-values < 0.05 and log2 fold change > 1) were used to identify enriched GO terms in both subsets. There were no terms enriched in the light green subset. Among the terms enriched in the dark green subset, only the top 12 with the lowest q-values were plotted. The dots are colored from light red to dark red based on −log10(q*-*value), from low to high. GeneRatio is the number of differentially expressed genes associated with a GO term divided by the total number of differentially expressed genes. **(F)** Integrated UMAP highlighting the Leiden cluster of B (Leiden 0), T lymphocytes (Leiden 1), and other cell types (gray) for gene-detection sensitivity comparison between USPPAR and 10× in Fig 6E. The Leiden clusters were inferred identically to Fig 6D, and barcodes belonging to B and T lymphocytes were colored. Mes: mesenchymal; Endo: endothelial; Gran: granulocytic; Dend: dendritic. Raw count data are available at GEO (GSE256014) and code for analysis and plotting at Zenodo (https://zenodo.org/records/13927875) for panels (B)–(F).
(TIF)

S11 Fig The CuC lysis buffer also enabled snRNA-seq from the murine pancreas. **(A)** Stacked violin plots showing scaled, normalized expression of marker genes across pancreatic cell types in Fig 7B (Independent CellType). The color scale ranges from white (lowest value) to dark blue (highest median expression). **(B)** UMAPs of 11,697 murine pancreatic nuclei (genes detected > 200), colored by cell-type annotation and expression of marker genes: ductal (Krt19, Krt8, Epcam, Cdh1), acinar (Pnlip, Try5, Ctrc, Cpa1, Ptf1a, Sox9, Prom1), islet (Pax6), endothelial (Cdh5), mesenchymal (Col1a1, Dcn), mesothelial (Msln, Upk1b), hematopoietic (Ptprc), and monocytic (Itgam). The color scale for gene expression ranges from dark purple (lowest normalized expression) to yellow (highest). In (A) and (B), acinar cells were subdivided into four subclusters (Acinar_0–3) using Leiden clustering (resolution 0.4). **(C)** Joint UMAP of 23,327 pancreatic nuclei based on CuC (CuC, 11,637 nuclei) and pH3 (pH3, 11,630) lysis systems. Colors denote cell-type annotations based on merged human pancreatic [25] and immune [55] datasets in the joint model (Joint) or in the USPPAR_pH3 (pH3) and USPPAR_CuC (CuC) models alone. Raw count data are available at GEO (GSE256014) and code for analysis and plotting at Zenodo (https://zenodo.org/records/13927875).
(TIF)

S12 Fig The CuC lysis buffer also enabled snRNA-seq from the murine liver. **(A)** UMAPs of 15,459 liver nuclei (200–2,500 genes/nucleus) based on CuC lysis system. Colors denote cell-type annotations from either the joint model of four technologies (Fig 7E; Transferred) or independent modeling of USPPAR_CuC datasets (Independent), inferred from the reference PanSci dataset. **(B)** Stacked violin plots showing scaled, normalized expression of marker genes across liver cell types in (A) (Independent CellType). The color scale ranges from white (lowest value) to dark blue (highest median expression). **(C)** UMAPs 15,459 liver nuclei in (A), colored by cell-type annotation and expression of marker genes: hepatic (G6pc, Hnf4a, Alb, Cyp2e1, and Lgr5), stellate (Reln and Dcn), ductal (Spp1 and Krt19), Kupffer (Adgre1 and Spic), mesenchymal (Col1a1, Pdgfra, and Eln), endothelial (Kdr and Cdh5), and hematopoietic (Ptprc, Cd3d, and Cd79b) markers. The color scale for gene expression ranges from dark purple (lowest normalized expression) to yellow (highest). Endo: endothelial. In (B) and (C), hepatic cells were subdivided into two subclusters (Hepatic_0–1) using Leiden clustering (resolution 0.2). **(D)** UMAPs of 15,459 liver nuclei in (A) (Independent CellType), split by treatment: ABF, EGS, or methanol only (MeOH). **(E)** Stacked bar plots showing the percentages of annotated cell types in the ABF, EGS, and MeOH treatment groups in (D). Chi-square tests with FDR-BH correction were used for statistical comparison of cell type distributions across conditions. **(F)** Schematic of using a looped oligo (black) to test sufficient T4 PNK activity (phosphorylation, P) and T4 DNA ligase activity (ligation) in (G). Successful treatment results in an upshift of the substrate oligo (green). **(G)** Silver-stained TBE-PAGE (20%) validating the activities of commercial T4 polynucleotide kinase (PNK) and T4 DNA ligase. 0.25 nmol of BC0T16V (CTCACTAACCATCACTCnnnnccggTTTTTTTTTTTTTTTTV) was treated with 0.5 U of commercial T4 PNK in 5 μL reaction at 37°C for 2 hours, followed by heating at 65°C for 20 min. Next, 0.4 μL (20 pmol) of phosphorylated BC0T16V (pBC0T16V) and 40 pmol of the looped oligo (loopO, TTAGTGAGAACTCCAGTCACtttGTGACTGGAGTT, arrowhead) were incubated in 20 μL of ligation reaction mixture containing Tris-HCl pH 7.5 (70 mM), MgCl_2_ (10 mM), DTT (5 mM), ATP (1 mM), and commercial T4 DNA ligase (40 U) at 37°C for 40 min to mimic the ligation conditions used during the Barcoding stage. Finally, 2.5 μL of the reaction was used for electrophoresis. Asterisk indicates that pBC0T16V (arrow) was almost completely converted to the loopO-to-BC0T16V ligation product. Lane 3 served as the no-PNK control, while lanes 1 and 2 contained 2.5 pmol of untreated BC0T16V and 5 pmol of loopO for reference. **(H)** UMAPs of 15,459 liver nuclei in (A) (Independent CellType), split by the use of commercial (Commercial) or homebrew (Homebrew) enzymes during the Barcoding stage. Nuclei prepared with commercial enzymes, including those with homebrew rRi during RT and pre-RT washes (Fig 7H, lane 6), are included in the ‘Commercial’ group. **(I)** Stacked bar plots showing the percentages of annotated cell types in the Commercial and Homebrew groups in (H). No significant differences in cell counts across conditions by chi-squared test with FDR-BH (all *q* > 0.05). Raw count data are available at GEO (GSE256014) and code for analysis and plotting at Zenodo (https://zenodo.org/records/13927875) for panels (A)–(E) and (H)–(I). Uncropped images of the agarose gel (G) are in S1 Raw Images.
(TIF)

S13 Fig Integration of snRNA datasets from all three murine organs profiled with USPPAR. Integrated UMAP of 62,335 nuclei from murine pancreas (S11C Fig), liver (Fig 7E; USPPAR_CuC and USPPAR_pH3), and spleen (Fig 6B), assayed with USPPAR using CuC or pH3 buffers in this study. The nuclei were colored according to their organs of origin (blue for liver, orange for pancreas, and green for spleen) and the expression of various cell markers: mesenchymal (Col1a1), endothelial (Cdh5), hematopoietic (Ptprc), B lymphocytic (Cd19), T lymphocytic (Cd3e), monocytic (Cd68), erythroid (Gypa), hepatic (G6pc), ductal (Krt19), acinar (Ctrc), and islet (Pax6, arrow). The dark purple color denotes the lowest normalized expression value, while yellow denotes the highest. Raw count data are available at GEO (GSE256014) and code for analysis and plotting at Zenodo (https://zenodo.org/records/13927875).
(TIF)

S14 Fig Additional data related to maize roots. **(A)** Violin and box plots showing gene detection from 2,500 subsampled reads per root barcode, prepared with USPPAR (USPPAR; 1,044 cells), or from the protoplasts in the reference 10× dataset (10×_v3; 3,266 cells). The horizontal bars in each box plot represent the upper quartile, median (indicated by its value), and lower quartile values, listed from highest to lowest. P-values are obtained using the Wilcoxon rank-sum test. **(B)** UMAPs of all 5,746 nuclei (genes detected > 120) from maize roots based on USPPAR alone. The nuclei were modeled independently of the reference dataset for low-dimensional embedding and normalized gene expression levels. The cells were colored according to the Leiden labels (with accompanying inferred cell types) and the expression of the following genes related to inferred lineages and cell-type specificities: LTPG5 (exodermis, 0), Zm00001d049073 (meristem, 2.0e−156), Zm00001d002850 (meristem, 3.5e−124), Zm00001d022280 (epidermis, 4.2e−212; hair, 2.8e−281), XTH10 (hair, 0), ACT12 (hair, 7.8e−106), Zm00001d016733 (cortex, 4.6e−173), Zm00001d007160 (cortex, 5.1e−221), Zm00001d052034 (protophloem, 0; endodermis, 0), XRN3 (protophloem, 0; endodermis, 0), Zm00001d028228 (xylem, 8.8e−124), Zm00001d043477 (xylem, 0), UMAMIT5 (stele, 3.7e−258), Zm00001d008285 (stele, 6.6e−200), Zm00001d047775 (endodermis, 0), CASPL1A1 (endodermis, 0), Zm00001d042922 (meristem, 6.3e−127; cap, 1.1e−77), GRXC1 (meristem, 8.8e−136;cap, 1.7e−69), GSTU1 (meristem, 1.5e−65; cap, 1.3e−67; companion cell, 1.1e−83), Zm00001d032822 (stem cell niche, 0), Zm00001d032821 (stem cell niche, 0), Zm00001d051478 (stem cell niche, 2.1e−95; epidermis, 0; pericycle, 0), Zm00001d019045 (pericycle, 1.1e−246; stem cell niche, 1.6e−94; epidermis, 0). The cell-type specificity is defined by the differential gene expression in each cell type against the others in the reference [60] (cutoff: adjusted *p* values = e−60). The dark purple color denotes the lowest normalized expression value, to yellow the highest. Raw count data are available at GEO (GSE256014) and code for analysis and plotting at Zenodo (https://zenodo.org/records/13927875).
(TIF)

S15 Fig Additional data related to maize shoots. **(A)** Scatter plot of a mixed-species experiment. Starting at the RT step, the shoot nuclei were intermixed with nuclei from mixed human (HEK293T cells and hPSCs) and mouse (OP9-DL1 cells) sources at the approximate 30:6:1 ratio based on visual counting using a hemacytometer. The human and mouse nuclei were prepared and fixed with the identical procedure, replacing the iodixanol-based density-gradient purification with direct centrifugation in lysis buffer. Only barcodes having more than 400 genes were kept. Green denotes barcodes with mouse reads accounting for more than 90% of all reads, blue denotes human reads, yellow denotes maize reads, and red denotes those not falling into any of these categories. The numbers and corresponding percentages for each category are annotated on the right side. **(B)** Joint UMAPs of 20,760 nuclei from maize shoot, combining reference and USPPAR datasets. Colors indicate nuclei from the reference (10×_v3, 5,927 nuclei) or from independent Dissociation and Barcoding procedures of USPPAR: USPPAR1 (9,981 nuclei) and USPPAR2 (4,852 nuclei). **(C)** UMAPs of all 14,833 nuclei (genes detected > 400) from maize shoots based on USPPAR alone. The nuclei were modeled independently of the reference dataset for 2D UMAP embedding and normalized gene expression levels. The nuclei are colored according to the transferred cell-type annotations shown in Fig 8E (CellType) and the expression levels of the following marker genes (cell type affiliations [27,60]): PEPC1 and MDH6 (mesophyll), DCT2 (bundle sheath), AIC2 (procambium meristem), RTL2 (vessel), NACTF103 and NACTF131 (xylem), ZmSWEET12 and zmSUT1 (phloem), KCH3 (guard cell), EPF2 (stomatal precursor), GPAT12 (epidermis), RD22 (epidermis/pavement cells), and KCS15 and Zm00001d025958 (epidermis/leaf rim). Arrows highlight the small populations that express their respective markers in the first plots of the xylem, phloem, and epidermis categories. The dark purple color denotes the lowest normalized expression value, to yellow the highest. Raw count data are available at GEO (GSE256014) and code for analysis and plotting at Zenodo (https://zenodo.org/records/13927875).
(TIF)

S1 Table Oligonucleotides used in this work. (XLSX)

S2 Table Summary of experiments. (PDF)

S3 Table Flow chart comparison of USPPAR with EasySci(Ez)/PanSci and SPLiT-seq (Evercode v3), showing each step and the time required. Advantages and disadvantages at each stage are indicated with O or X. Asterisks mark potential multiplexing barcoding steps. In the USPPAR column, each time point is preceded by a number corresponding to the experimental step shown in Figs 1 and S1A.
(PDF)

S4 Table Estimating the cost of reagents using USPPAR compared to the other three high cell-capacity methods, SPLiT-seq [10], EasySci(Ez) [12], and sci-RNA-seq3 [49], which are also special-reagent and equipment-free. Only the costs associated with commercial enzymes are shown here, as they are the primary budget bottleneck. In contrast, the costs of other reagents, such as oligonucleotide synthesis and the homebrew enzymes used with USPPAR (#), become insignificant when spread across multiple experiments. The costs are based on a 3-round barcoding approach for all methods, considering 100,000 subsampled cells for library preparation, as stated in the protocols provided with sci-RNA-seq3. The cost of post-lysis library preparation for SPLiT-seq is assumed to be 1 reaction for all 100,000-cell lysate, while USPPAR uses 10 separate reactions because a high TdT-to-cell ratio is critical for completely extending cDNA and unligated adapters. The non-empty cells containing reagents and enzymes are highlighted with a yellow background. The last column shows the USPPAR procedure steps (B: Barcoding; A: Amplification; see S1 Text) and the amounts of enzymes used (in parentheses).
(PDF)

S1 Text Detailed experimental steps and accompanying suggestions for USPPAR. (PDF)

S1 Data Individual numerical values for the corresponding figure panels. (XLSX)

S1 Raw Images Original, uncropped, and minimally adjusted images supporting all blot and gel results presented in the manuscript. (PDF)

## Abbreviations:

ABF: 4-azidobenzoyl fluoride
BSA: bovine serum albumin
DEPC: diethyl pyrocarbonate
DTT: dithiothreitol
EDTA: ethylenediaminetetraacetic acid
EGS: ethylene glycol bis(succinimidyl succinate)
FBS: fetal bovine serum
GO: Gene Ontology
IMAC: immobilized metal affinity chromatography
NTA: nitrilotriacetic acid
PBMCs: peripheral blood mononuclear cells
PFA: paraformaldehyde
PMSF: phenylmethylsulfonyl fluoride
PNK: polynucleotide kinase
PTFE: polytetrafluoroethylene
rRi: rat ribonuclease inhibitor
RT: reverse transcription
scRNA: single-cell RNA
SDS: sodium dodecyl sulfate
S.I.: SUPERase·In
snRNA-seq: single-nucleus RNA sequencing
STA: single-tube preamplification
TdT: terminal deoxynucleotide transferase
USPPAR: Unified framework by Split-Pool barcoding with optimal-efficiency PolydeoxyAdenylation for scRNA detection
