# Supplementary material for: USPPAR is a cost-effective, scalable, and highly sensitive single-cell RNA sequencing workflow compatible with diverse specimens
Source: PLoS Biol. 2025 Dec 15;23(12):e3003537. doi: 10.1371/journal.pbio.3003537 (PMC12704895; doi:10.1371/journal.pbio.3003537)
Supplement: S2 Table — (PDF) [file pbio.3003537.s017.pdf]

Sample ID	Target Cells	Spike-in	Cell # in single exp.	Cells in plots	Design	Tissue Lysis Buffer	Barcoding Rounds	PEG8K (7.5%) during RT	N9 during RT	TdT per library	Illumina Platform	Associated Figures	GEO Samples	Folder Names
1	HEK293T cells & MS5 cells		100,000	1,204	Benchmark against the other scRNA-seq methods.		3	yes	1-48: no; 49-96: yes	commercial, 2 µL	HiSeq X Ten	S2J-L, 3, S4	GSM8526470-2	S1a-c
2	HEK293T cells		48,000	830	The direct fixation in methanol versus an additional step of glyoxal fixation.		3A	yes	yes	commercial, 2 µL	NovsSeq X Plus	S2I	GSM9204532-5	S2a-d
3	mouse pancreas	hPSCs	400,000	11,630	Test for one-pot collection of nuclei using the RNase-laden pancreas in a pH 3 citrate buffer.	pH3 buffer	3	yes	yes	commercial, 2 µL	HiSeq X Ten	7C-D, S6E-G, S13	GSM8526474-9	S3a-f
4	mouse liver and spleen	hPSCs	400,000	17487(liver)+ 1,607(spleen)	Test for one-pot collection of nuclei using the liver and RNase-laden spleen in a pH 3 citrate buffer.	pH3 buffer	3	yes	yes	commercial, 2 µL	HiSeq X Ten	7E-G, S7, S8, S10B-C, S11C, S13	GSM8526480-1	S4a-b
5	mouse spleen		400,000	4,455	Test for one-pot collection of splenic nuclei using our non-conjugating RNase-blocking CuC compex.	CuC bufer	3	yes	no	commercial, 2 µL	HiSeq X Ten	6B-E, S10B-F, S13	GSM8526482-8	S5a-g
6	maize root	hPSCs	400,000	5,746	Compare the spermidine- and Mg^{2+}-containing lysis for one-pot collection of nuclei from maize roots.	Spermidine- or M^{2+}-based CuC buffer	4	yes	no	commercial, 2 µL; homebrew, 900 ng	HiSeq X Ten	S2G, 8B-C, S14	GSM8526489-90	S6a-b
7	maize shoot	HEK293T cells, hPSCs, and OP9-DL1 cells	400,000	9,981	Test for one-pot nuclear collection from maize shoots using the optimized buffer.	M^{2+}-based CuC buffer	4	yes	no	commercial, 2 µL; homebrew, 900 ng	HiSeq X Ten	S2G-H, 8D-E, S15	GSM8526491-2	S7a-b
8	HEK293T cells		48,000	2,792	Test N6 or N9 primers, varying incubation temperatures, and different types and amounts of reverse transcriptase.		3A	yes	yes (N9 or N6)	commercial, 2 µL	NovsSeq X Plus	S2D	GSM9204536	S8
9	human PBMCs		48,000	8,176	Benchmark against the other scRNA-seq methods. Tested methanol fixation alone or in combination with ABF conjugation.		3A	yes	yes	commercial, 2 µL	NovsSeq X Plus	4, S5	GSM9204537-9	S9a-c
10	mouse pancreas		96,000	11,697	Test one-pot collection of pancreatic nuclei using CuC complex.	CuC bufer	3A	yes	yes	commercial, 2 µL	NovsSeq X Plus	7B-D, S11, S13	GSM9204540-2	S10a-c
11	mouse liver		96,000	10922	Test one-pot collection of liver nuclei using the CuC complex with either commercial or homebrew enzymes, and methanol fixation alone or combined with EGS conjugation.	CuC bufer	3A	yes	yes (N6)	commercial, 2 µL	NovsSeq X Plus	7E-H,S12A-E, S12H-I	GSM9204543-7	S11a-e
12	mouse liver		48,000	4,537	Test one-pot collection of liver nuclei using the CuC complex with either commercial or homebrew enzymes, and methanol fixation alone or combined with ABF or EGS conjugation.	CuC bufer	3A	yes	yes (N6)	commercial, 2 µL	NovsSeq X Plus	7E-H,,S12A-E, S12H-I	GSM9204548-52	S12a-e
13	maize shoot		48,000	4,852	Test for one-pot nuclear collection from maize shoots using the optimized buffer.	M^{2+}-based CuC buffer	3A	yes	no	commercial, 2 µL	NovsSeq X Plus	8D-E, S15B-C	GSM9204553-5	S13a-c
