# Supplementary material for: USPPAR is a cost-effective, scalable, and highly sensitive single-cell RNA sequencing workflow compatible with diverse specimens
Source: PLoS Biol. 2025 Dec 15;23(12):e3003537. doi: 10.1371/journal.pbio.3003537 (PMC12704895; doi:10.1371/journal.pbio.3003537)
Supplement: S3 Table — Advantages and disadvantages at each stage are indicated with O or X. Asterisks mark potential multiplexing barcoding steps. In the USPPAR column, each time point is preceded by a number corresponding to the experimental step shown in Figs 1 and S1A. (PDF) [file pbio.3003537.s018.pdf]

| Step | USPPAR | EasySci (Ez) | SPLiT-seq |
| --- | --- | --- | --- |
| **Dissociation** | O End-to-end;<br>O CuC and EGS/ABF mediated RNase inhibition;<br>O Validated with RNase-rich spleen/pancreas | O End-to-end;<br>O DEPC-mediated RNase inhibition;<br>? Not tested on RNase-rich tissues | ? Not well characterized |
| **Barcoding** | 4–5 rounds (incl. final multiplexing PCR);<br><br>O Greater scalability;<br>X Longer time | Fixed 3 rounds (incl. final multiplexing PCR);<br><br>X Less scalability;<br>O Shorter time | 4–5 rounds (incl. final multiplexing PCR);<br><br>O Greater scalability;<br>X Longer time |
| **Reverse Transcription (RT)** ✻ | ① 60 min | 60 min | 75 min |
| **Ligation 1** ✻ | ② 75 min | 75 min | 60 min |
| **Ligation 2** ✻ | ③ 75 min | — | 30 min |
| **(Ligation 3)** ✻ | (④ 75 min) | (—) | (30 min) |
| **Amplification** | O Preamplification;<br>O High-efficiency cDNA tailing (TdT);<br>O Purification after preamplification to avoid loss;<br>O 2 rounds purification | X No cDNA preamplification;<br>X First purification before preamplification (loss risk);<br>O Shorter time;<br>O 2 rounds purification | O Preamplification;<br>X First purification before preamplification (loss risk);<br>X Incomplete cDNA tailing (template-switch);<br>X 5 rounds purification (longer time, more loss) |
| **Lysis** | 50 min | — | 60 min |
| **Purification 1** | — | — | 60 min (Streptavidin–biotin cDNA capture) |
| **Adding Handle for Preamplification** | ⑤ 90 min (TdT tailing) | — | 120 min (Template-switch) |
| **2nd Strand Synthesis** | ⑥ 50 min (after poly(dA) tailing) | 105 min | — |
| **Preamplification** | ⑦ 70 min | — | 90 min |
| **Purification 2** | 60 min (incl. quantification) | 60 min | 60 min (incl. quantification) |
| **Tagmentation** ✻ | ⑧ 45 min | 55 min | 60 min (enzymatic fragmentation) |
| **Purification 3** | — | — | 30 min |
| **Adapter Ligation** | — | — | 30 min |
| **Purification 4** | — | — | 30 min |
| **Library PCR** ✻ | ⑨ 75 min | 45 min | 45 min |
| **Purification 5** | 60 min | 60 min | 30 min |
| **Total Time – Day 1** | 270 min | — | 285 min |
| **Total Time – Day 2** | 450 min | 520 min | 495 min |
