# Supplementary material for: USPPAR is a cost-effective, scalable, and highly sensitive single-cell RNA sequencing workflow compatible with diverse specimens
Source: PLoS Biol. 2025 Dec 15;23(12):e3003537. doi: 10.1371/journal.pbio.3003537 (PMC12704895; doi:10.1371/journal.pbio.3003537)
Supplement: S4 Table — Only the costs associated with commercial enzymes are shown here, as they are the primary budget bottleneck. In contrast, the costs of other reagents, such as oligonucleotide synthesis and the homebrew enzymes used with USPPAR (#), become insignificant when spread across multiple experiments. The costs are based on a 3-round barcoding approach for all methods, considering 100,000 subsampled cells for library preparation, as stated in the protocols provided with sci-RNA-seq3. The cost of post-lysis library preparation for SPLiT-seq is assumed to be 1 reaction for all 100,000-cell lysate, while USPPAR uses 10 separate reactions because a high TdT-to-cell ratio is critical for completely extending cDNA and unligated adapters. The non-empty cells containing reagents and enzymes are highlighted with a yellow background. The last column shows the USPPAR procedure steps (B: Barcoding; A: Amplification; see S1 Text) and the amounts of enzymes used (in parentheses). (PDF) [file pbio.3003537.s019.pdf]

Enzyme	Cost/µL	sci-RNA-seq3	EasySci (Ez)	SPLiT-seq	USPPAR	Step (Amounts)
**Superscript IV**	$7.11	55 µL($391)				
**Maxima H Minus Reverse Transcriptase (Thermo, EP0753)**	$3.77		52.5 µL($197.92)	222.2 µL($837.69)		
**SuperaseIN**	$1.00			43.4 µL($43.4)		
**Enzymatics RNase-In (Qiagen, Y9240L, 40U/ul)**	$1.19			98.7 µL($117.45)		
**M5 reverse transcriptase**	#				10.20 micrograms	B4 (5 ng/µL*20 µL*102)
**Rat RNase inhibitor**	#				34.51 micrograms	B3 (5 ng/µL*200 µL*2)<br>B4 (5 ng/µL*102 µL+5 ng/µL*20 µL*102)<br>B9 (0.5 ng/µL*200 µL*2*2)<br>B11 (0.5 ng/µL*200 µL*2*2)<br>B12 (0.5 ng/µL*102*2 µL + 5 ng/µL*20 µL*102*2)<br>B18 (0.5 ng/µL*200 µL)<br>B19 (0.5 ng/µL*100 µL)<br>B20 (0.5 ng/µL*200 µL)<br>B23 (0.5 ng/µL*200 µL)<br>B24 (0.5 ng/µL*200 µL)<br>B27 (0.5 ng/µL*3 µL *30)
**T4 DNA Ligase**	$1.04	65 µL ($68)	52.5 µL ($54.6)			
**T4 DNA Ligase (NEB, M0202L)**	$1.15			200 µL($230)		
**T4 DNA Ligase**	#				16.32 micrograms	B12 (4 ng/µL*20 µL*102*2)
**Second Strand Synthesis**	$2.95	35 µL ($103)	35 µL ($103)			
**NEBNext 2X PCR mix**	$0.06	2200 µL ($127)	2200 µL ($127)			
**Kapa Hifi 2x Master Mix**	$0.13			148.5 µL($19.31)		
**TdT (Qiagen, P7070L, Qiagen)**	$1.48				20 µL($29.6)	A3 (2 µL*10)
**KAPA HiFi (Roche, KK2102)**	$1.24				56.25 µL($69.75)	A8 (2 µL*10)<br>A10 (2 µL*10)<br>A28 (0.125 µL*10)<br>A30 (1.5 µL*10)
**Tn5**	$24.50	4.6 µL ($112)				
**Amplicon tagmentation enzyme & Nextera PCR mix (Nextera XT-DNA Sample Prep Kit (96))**	$25.68*			1 reaction ($25.68)		
**Tn5 (106 ng/µL)**	#				0.53 micrograms	A25 (0.5 µL*10)
**Total enzyme cost per plate (96×96×96)**		**$801**	**$482.52**	**$1,273.53**	**$99.35**	
