# Supplementary material for: USPPAR is a cost-effective, scalable, and highly sensitive single-cell RNA sequencing workflow compatible with diverse specimens
Source: PLoS Biol. 2025 Dec 15;23(12):e3003537. doi: 10.1371/journal.pbio.3003537 (PMC12704895; doi:10.1371/journal.pbio.3003537)
Supplement: S1 Text — (PDF) [file pbio.3003537.s020.pdf]

USPPAR Supporting Protocol

Table of Contents

MATERIALS 1

REAGENTS AND SOLUTIONS 1

Support Protocol 1: Oligo Preparation for RT and Ligation 4

- Verification of sufficient amounts of T4 PNK and DNA ligase 4
- Official PNK treatment of oligonucleotides 5
- Anneal Barcoding adapters for ligation-mediated barcoding 6

Support Protocol 2: Assemble & test Tn5 transposase complex 7

- Anneal mosaic-end oligoes and load to Tn5 7

BEFORE GETTING STARTED 9

- Optimizations 1: Enzymes, RNase Inhibition, and DNA Processing (step O1) 9
- Optimizations 2: Cellular samples - Barcoding and Amplification (step O2) 10
- Optimizations 3: Tissue samples - Dissociation (step O3) 12

DISSOCIATION STAGE (step D1-D13) 16

BARCODING STAGE 22

- Reverse Transcription (1st-Round Barcoding) (step B1-B7) 22
- Ligation-Mediated Barcoding (2nd and subsequent rounds) (step B8-B16) 24
- Aliquot Barcoded Cells for Long-Term Storage (step B17-B27) 25

AMPLIFICATION STAGE 27

- Lysis and Poly(dA)-Tailing of cDNA (step A1-A5) 27
- Single-Tube 2nd-Strand Synthesis and Preamplification (step A6-A13) 28
- Purification and Quantification of cDNA (step A14-A24) 29
- cDNA Tagmentation (step A25-A27) 30
- Library Amplification (step A28-A32) 31
- Size-selection for the library (step A33-A44) 32

USPPAR Supporting Protocol

MATERIALS

$CuSO_4$ (CAS 7758-98-7);
Sodium citrate (CAS 6132-04-3);
Halt protease inhibitor (ThermoFisher);
Nylon membrane, 800-mesh size (LVBANG filter);
Polyvinyl alcohol (PVA; 87-90%-hydrolyzed; Sigma, P8136);
Hydrophilic PTFE membrane, 3 µM pore size (Delvstlab);
OptiPrep (iodixanol 60%; ProteoGenix, 1114542);
EGS (ethylene glycol bis(succinimidyl succinate)) (CovaChem, 13308-100);
T4 PNK (Enzymatics, Y9040L);
T4 DNA ligase (Lucigen, F83911-1, 120 U/µL);
Maxima H Minus Reverse Transcriptase (ThermoFisher, EP0752);
Terminal deoxynucleotidyl Transferase (TdT, Enzymatics, P7070L);
KAPA HiFi (Roche, KK2102);
carboxylate paramagnetic particles (Cytiva, 65152105050250);
PEG8000 (Sigma, P2139);
SYBR Green I, 10,000X (Lumiprobe, 20010)

REAGENTS AND SOLUTIONS

10X Annealing buffer

Component	Amount (µL)	Final Centration
Milli-Q water	764.7	-
Tris-HCl pH 8, 1 M	100	100 mM
potassium acetate, 3 M	133.3	400 mM
EDTA, 500 mM	2	1 mM
Total	1000	-

10X PNK buffer

Component	Amount (µL)	Final Centration
Milli-Q water	50	-
Tris-HCl pH 7.5, 1 M	700	700 mM
$MgCl_2$, 1 M	100	100 mM
DTT, 1 M	50	50 mM
ATP, 100 mM	100	10 mM
Total	1000	-

10X RT buffer

Component	Amount (µL)	Final Centration
Milli-Q water	170	-
Tris-HCl pH 8.3, 1 M	500	500 mM
KCl, 3 M	250	750 mM
DTT, 1 M	50	50 mM
$MgCl_2$, 1 M	30	30 mM
Total	1000	-

10X Tagmentation buffer

Component	Amount (µL)	Final Centration
Milli-Q water	850	-
Tris-HCl pH 8.5, 1 M	100	100 mM
$MgCl_2$, 1 M	50	50 mM
Total	1000	-

10X TdT buffer

Component	Amount (µL)	Final Centration
Milli-Q water	700	-
Tris-acetate pH 7.9, 1 M	200	200 mM
Triton X-100, 10%	100	1%
Total	1000	-

5X Quench buffer

Component	Amount (µL)	Final Centration
Milli-Q water	933	-
SDS, 10%	25	0.25%
EDTA, 500 mM	42	21 mM
Total	1000	-

loTE

Component	Amount (µL)	Final Centration
Milli-Q water	989.8	-
Tris-HCl pH 8, 1 M	10	10 mM
EDTA, 500 mM	0.2	0.1 mM
Total	1000	-

USPPAR Supporting Protocol

Low-salt buffer

Component	Amount (µL)	Final Centration
Milli-Q water	968.27	-
Tris-HCl pH 8, 1 M	10	10 mM
KCl, 3 M	0.33	1 mM
PVA, 10%	10	0.1%
EDTA, 500 mM	0.2	0.1 mM
Rat RNase inhibitor, 445 ng/µL	11.2	5 ng/µL
Total	1000	-

PEG/NaCl solution

Component	Amount (µL)	Final Centration
Milli-Q water	83	-
Tris-HCl pH 8, 1 M	10	10 mM
PEG8000, 50%	400	20%
NaCl, 5M	500	2.5 M
EDTA, 500 mM	2	1 mM
Tween 20, 10%	5	0.05%
Total	1000	-

Support Protocol 1: Oligo Preparation for RT and Ligation

<u>Verification of sufficient amounts of T4 PNK and DNA ligase</u>

1. Test the optimal amount of PNK by preparing the following mixture:

Component	Amount (µL)	Final Centration
Milli-Q water	Variable	-
Oligonucleotides in loTE, 100 µM	10	50 µM
10X PNK buffer	2	1X
T4 PNK	Variable*	Homebrew: 0, 100, or 300 ng<br>Commercial: 0, 2, or 6 U
Total	20	-

*When using commercial T4 PNK, 2 units of enzyme were used for every 1 nmol of oligonucleotide substrate. One unit of PNK is defined as the amount of enzyme that catalyzes the incorporation of 1 nmol of $[^{32}P]$-ATP into substrate in 30 minutes at 37 °C in the standard reaction buffer. Nevertheless, before use, each batch of homebrewed or commercial T4 PNK should be tested by a pilot phosphorylation assay to ensure sufficient enzyme activity for phosphorylation.

2. Conduct the reaction at 37°C for 2 hours, followed by heat inactivation at 65°C for 20 minutes.

*Although phosphorylated oligonucleotides migrate faster (appearing lower on the gel), this difference is usually too subtle for reliable detection. Thus, we recommend using a looped oligonucleotide that ligates with the phosphorylated oligonucleotide as a more reliable assay (S12F-G Figs), as described below. Upon successful ligation, the larger conjugated product migrates to a higher position on the gel compared with the parental oligonucleotide, providing clearer visual confirmation of phosphorylation. Additionally, this assay validates the activity of the T4 DNA ligase used in the Barcoding stage.

3. Test the optimal amount of T4 DNA ligase by preparing the following mixture:

Component	Amount (µL)	Final Centration
Milli-Q water	Variable	-
Oligonucleotides, 50 µM (5′-phosphorylated, synthesized; ± PNK, as described above)	0.4	1 µM
Looped oligonucleotide, 50 µM	0.8	2 µM
10X PNK buffer	2	1X
T4 DNA ligase	Variable*	Homebrew: 0, 80 or 240 ng<br>Commercial: 0, 40 or 120 cohesive-end units
Total	20	-

*For ligation reactions using commercial T4 DNA ligase, we used 40 cohesive-end units of T4 DNA ligase per 1 µM of adapters in a 20 µL reaction (20 pmol potential ligation ends). Note that T4 DNA ligase activity is reported in two unit definitions: Weiss units and cohesive-end units (1 Weiss unit

equals approximately 67 cohesive-end units). To optimize enzyme concentration, we recommend performing a titration using a looped oligonucleotide (40 pmol) and phosphorylated substrate (20 pmol) combined with different amounts of T4 DNA ligase. In this assay, we use a 2-fold molar excess of looped oligonucleotide relative to the phosphorylated substrate to ensure complete ligation of all phosphorylated molecules. The unphosphorylated and chemically synthesized phosphorylated oligonucleotides serve as the negative and positive controls for PNK treatment, respectively. Note that the 5' 8-bp protrusion of the looped oligo shall be complementary to the 5' end of the phosphorylated substrate to test for ligation. The oligonucleotides 'BC0T16V' and 'loopO' listed in Table S1, which were used for S12G Fig, can serve as model substrates for this ligation test.

4. Incubate at 37°C for 40 minutes.
5. Add 20 µL of 2X sample buffer (95% formamide, 0.025% SDS, 0.025% bromophenol blue, 0.025% xylene cyanol FF, 0.5 mM EDTA), heated at 95°C for 10 minutes.
6. Load 5 µL for denaturing urea polyacrylamide gel electrophoresis and silver staining, as described in the Methods section.

**Using chemically synthesized phosphorylated oligonucleotides, a sufficient amount of T4 DNA ligase results in an almost complete upshift of the original oligo to a higher position (e.g., arrow to asterisk in S12G Fig). At this ligase concentration, an adequate amount of T4 PNK produces a similar upshift compared with the unphosphorylated oligo control. The range of T4 PNK and DNA ligase can be further refined based on the results above, leaving a safety margin (e.g., 1.5-2x the enzyme amount sufficient for complete reaction) to ensure the reaction reaches completion every time.*

Official PNK treatment of oligonucleotides

Component	Amount (µL)	Final Centration
Milli-Q water	7.8	-
Oligonucleotides in loTE, 100 µM	10	50 µM
10X PNK buffer	2	1X
T4 PNK, 491 ng/µL	0.2	5 ng/µL*
Total	20	-

**The amount is determined by the pilot assays described above. For reference, when using commercial T4 PNK, we used 2 units of enzyme per 1 nmol of oligonucleotide substrate.*

7. Conduct the reaction at 37°C for 2 hours, followed by heat inactivation at 65°C for 20 minutes.

**For the first-round RT oligos, add 20 µL of EDTA (11.6 mM) to the reaction and then dilute the oligos to 5 µM by adding 160 µL of loTE.*

Anneal Barcoding adapters for ligation-mediated barcoding

8. Prepare the following mixture for the annealing:

Component	Amount (all)	Final Centration
Milli-Q water	5* or 6	-
Phosphorylated (for BC1 and 1.5) or unphosphorylated (for BC2) oligonucleotides, 50 µM	20	25 µM
(EDTA, 232 mM)<br>**Only required for PNK treated oligo to quench free* Mg^{2+}	(1)*	5.8 mM
Respective splinter oligonucleotides in loTE, 100 µM	10	25 µM
10X annealing buffer	4	1X
Total	40	-

9. Conduct the annealing by incubating at 94°C denaturation for 1 minute, followed by a stepwise 1°C decrease per minute from 80°C to 20°C.
10. Dilute the annealed product to a final concentration of 5 µM using 160 µL of 1X annealing buffer.

The annealed adapters can be stored at -80°C for years.

Support Protocol 2: Assemble & test Tn5 transposase complex

Anneal mosaic-end oligoes and load to Tn5

1. Prepare the following mixture to obtain the annealed Tn5 adapter:

Component	Amount (µL)	Final Centration
Milli-Q water	11	-
pTn5Merev, 400µM	5	50 µM
i5ME, 100 µM	20	50 µM
10X Annealing buffer	4	1X
Total	40	-

2. Conduct the annealing by incubating at 94°C denaturation for 1 minute, followed by a stepwise 1°C decrease per minute from 80°C to 20°C.
3. Prepare the following mixture to load the adapter onto Tn5:

Component	Amount (µL)	Final Centration
Milli-Q water	5.2	-
Annealed duplex, 50 µM	3.6	5.625 µM
Tn5, 6 µM (320 ng/µL)	20	3.75 µM
10X Annealing buffer	3.2	1X
Total	32	-

4. Incubate at RT for 1 hour
5. Add glycerol to the loaded Tn5 for long-term storage at -20°C:

Component	Amount (µL)	Final Centration
Loaded Tn5 (already containing 2 µL glycerol)	32	2 µM
Glycerol, 100%	28	46.7%
Total	60	-

6. Prepare test tagmentation mixtures to optimize the amount of Tn5 for tagmentation:

Component	Amount (µL)	Final Centration
Milli-Q water	7-0.25, 0.5, 0.75 or 1	-
Plasmid carrier, 100 ng/µL	1	100 ng/10 µL
10X Tagmentation buffer	1	1X
DMF, 100%	1	10%
Loaded Tn5, 2 µM	0.25, 0.5, 0.75, or 1	0.05, 0.1, 0.15, or 0.2 µM
Total	10	-

7. Incubate at 55C for 30 minutes.
8. Stop the reactions and strip Tn5 from the tagmented DNA by adding 2.5 µL of 5X Quench buffer and heating the reaction at 55°C for 10 minutes.

9. Run half of the reactions on a 0.8% agarose gel to determine the optimal amount of loaded Tn5, aiming for the center of the smear at approximately 800 bp.

USPPAR Supporting Protocol

BEFORE GETTING STARTED

Since this is a multistage method involving many steps and reagents, we recommend that users become familiar with it in a sequential manner, starting from simpler samples such as cultured cells (which mainly involve only the Barcoding and Amplification stages) and then progressing to more complex samples such as nuclei from tissues (which include additional Dissociation stages). The following section outlines the recommended learning sequence and highlights the critical points for each stage.

Optimizations 1: Enzymes, RNase Inhibition, and DNA Processing

O1. To ensure successful library preparation, comprehensive quality control and optimization of enzymes, RNase inhibition, and DNA processing are required:

a. Optimize the concentration of T4 PNK to ensure complete phosphorylation of oligos for barcode ligation, assessed by denaturing PAGE and silver staining (see Support Protocol 1, steps 1-6).

b. Optimize the concentration of T4 DNA ligase to achieve complete ligation of phosphorylated substrate oligos, assessed by denaturing PAGE and silver staining (see Support Protocol 1, Steps 3-6).

c. Order oligos for the RT①, ligation②, and ligation③ for Barcoding (one sequence from the 96 possible in the 'BC0T16V', 'BC1', and 'BC2' tabs in Table S1). Perform phosphorylation of the BC0T16V and BC1 oligos as described in Support Protocol 1, step 7. Then, perform annealing of pBC1 with Splinter-1 and BC2 with Splinter-2 as described in Support Protocol 1, step 8. Finally, perform ligation (pBC0T16V with annealed pBC1, and annealed pBC1 with annealed BC2) following the strategy shown in S2B Fig. These steps validate the optimized concentrations of T4 PNK and T4 DNA ligase from steps O1a and O1b, as well as the correct ligation of the oligo combinations.

d. Perform quality control for the reverse transcriptase when using a homebrew preparation (strategy as shown in S2C Fig).

**The assay in S2C Fig uses lysate from 500 cells and 50 ng of homebrew M5 for reverse transcription in a 20 µL reaction volume. This serves only as a preliminary qualitative test to confirm that M5 can synthesize cDNA. In addition to this preliminary assay, an official assay designed to determine the sufficient amount of enzyme under the actual USPPAR reaction conditions is described below in step O2b.*

e. Perform quality control for the recombinant RNase inhibitor when using a homebrew preparation (strategy as shown in S2D Fig).

Under the specified reaction conditions, the recombinant RNase inhibitor should strongly suppress degradation of the fluorescent reporter. For guidance on establishing a more precise benchmark against commercial enzymes, refer to step B3*. In addition to the test using RNaseAlert (which may not fully reflect the context of true USPPAR), a more*

context-appropriate assay for evaluating recombinant and commercial RNase inhibitors is described below in step O2b (cells) and step O3e (tissues, which generally contain higher RNase levels).

f. If a homebrew TdT is used, perform quality control to test its tailing efficiency (Fig 2E). Cell lysate is not required at this stage; instead, use 5 pmol of annealed 3'-Recessed Duplex as the substrate, 10 U of commercial TdT as the positive control, no enzyme as the negative control, and various amounts of homebrew TdT (e.g., 100, 200, 400, and 800 ng) for the tailing reaction. Analyze the products by denaturing gel electrophoresis followed by silver staining to determine the approximate amount of homebrew TdT equivalent to 20 U of the commercial enzyme.

g. Assemble and test the Tn5 transposase complex to determine the optimal amount of loaded Tn5 complex required for 100 ng of DNA substrate (see Support Protocol 2).

h. Determine the appropriate concentration of PEG8000 required to achieve different DNA size cutoffs (see step A14*).

Optimizations 2: Cellular samples - Barcoding and Amplification

O2. After completing validations described above, we recommend performing a simple mixed-species experiment using RNA-abundant human (e.g., HEK293T) and mouse (e.g., NIH3T3) cells. The dissociated cells are fixed in ice-cold methanol, mixed at a 1:1 or 1:2 ratio, and then subjected to the following steps:

a. Take 10,000 mixed cells in methanol and add PVA (10% in Milli-Q water) to a final concentration of 0.1%. Centrifuge at 1,000 × g for 1 minute and perform a 10-time wash to ensure that cell loss remains within acceptable limits, as described in step B3**.

b. Perform the official USPPAR procedure as described in the Barcoding and Amplification stages. We recommend performing three rounds of 48 × 48 × 48 well reactions during the Barcoding stage and taking a 3,000-cell aliquot for the Amplification stage. Taking advantage of differential barcoding, we recommend performing additional parameter validation during the reverse transcription (RT) step. The table below provides an example of such a test, but users can design their own parameter combinations based on their experimental needs. For example, testing 1,000 and 2,000 cells can help determine whether cDNA conversion is approaching saturation in these RNA-abundant cell lines. Additionally, testing different combinations of RT enzymes and, similarly, RNase inhibitors allows assessment of their performance relative to commercial reagents. A sequencing depth of 60 million read pairs should be more than sufficient to assess the quality of the output, as demonstrated by: the mixed-species plot in Fig

3A, showing that each barcode captures transcripts from a single species with minimal cross-species reads; the number of genes and UMIs detected per barcode at subsampled read depths in S4B-C Figs; and the effects of input cell numbers, enzyme types, and concentrations, as shown in S2D Fig. Based on these results, parameters can be rapidly adjusted or optimized to maximize performance.

Row	Use [X] cells per well in a 20 µL reaction.	Reverse Transcriptase	RNase inhibitor
A	1,000	Commercial Maxima H- 100 U	Commercial 5 U
B	1,000	Commercial Maxima H- 100 U	Homebrew 100 ng
C	1,000	M5 100 ng	Homebrew 100 ng
D	2,000	Commercial Maxima H- 100 U	Commercial 5 U
E	2,000	Commercial Maxima H- 200 U	Commercial 5 U
F	2,000	M5 300 ng	Homebrew 100 ng
G	1,000	M5 300 ng	Homebrew 100 ng
H	2,000	Commercial Maxima H- 100 U	Commercial 15 U

c. If the small-scale pilot experiment (48 × 48 × 48) works with the model cells above, proceed to full-scale, high-multiplicity barcoding experiments (96 × 96 × 96) using your cells of interest when more than 50,000 nuclei are required. After Barcoding, split nuclei into 10,000-20,000 nuclei aliquots, with each aliquot used in a separate Amplification reaction (e.g., 10 parallel reactions with 10,000 nuclei each yields 100,000 total nuclei). This approach ensures complete tailing of all cDNA and adapters/primers and minimizes barcode collisions between different cells. We recommend performing a pilot test to confirm complete tailing using an aliquot of lysates in step A4*.

d. If cells are known to be rich in nucleases, such as PBMCs, lysine conjugators can be added to methanol fixed cells to reduce RNase activity (Fig 4A). We recommend verifying the effectiveness of RNase inactivation with lysine conjugation, especially if ABF is prepared in house, using the experimental design described in Fig 4B. Even when most cells are treated with lysine conjugators, we suggest leaving one or two rows(columns) of untreated cell controls (methanol fixation only) to confirm the effectiveness of the treatment. Even though the untreated cells contain a lower number of detected genes (Fig 4A), they are still useful for downstream analysis because they show a similar cellular composition (S5D Fig) and integrate well with the treated cells (S5C Fig).

Optimizations 3: Tissue samples - Dissociation

O3. Once the USPPAR protocol is successfully established with cells, and snRNA-seq on tissues is the next goal, we recommend starting with low-nuclease organs such as liver (S9F Fig) for benchmarking. Tissues with lower nuclease activity are easier to optimize initially and involve fewer troubleshooting parameters:

a. Follow the standard protocol (black, upright font) for steps D1-D13.
b. To become familiar with the extraction procedure, use a spare liver (not intended for official snRNA-seq, as validation of each step can be time-consuming) and take aliquots from every step, including the supernatant after centrifugation. These aliquots serve specific diagnostic purposes. For example:
    i. The morphology of fragments in the lysis buffer before and after pestling provides a rough idea of nuclear release.
    ii. Lysates before and after the 800-mesh nylon wash indicate the efficiency of large-fragment removal.
    iii. Aliquots from different layers after density-gradient centrifugation (step D8*) reveal whether most or all nuclei are correctly positioned at the interface, minimizing nuclear loss and biased representation of cell types.

    **Since some aliquots may contain only a few nuclei, an additional centrifugation at 1,000 × g for 2 minutes can help concentrate them for more confident identification. If the aliquot contains iodixanol, dilute it with four times the volume of tissue lysis buffer containing 0.1% PVA before centrifugation to ensure proper pelleting.*

    iv. The filtrate beneath the PTFE membrane after washing confirms that the membrane assembly is tight and leak-free.
    v. Nuclei after methanol fixation, especially following EGS treatment, should not be severely clumped.

    **To visualize nuclei after methanol fixation, add PVA to a final concentration of 0.1%, centrifuge at 1,000 × g for 1 minute, and resuspend in PBS for proper observation.*

**H33258 nuclear staining (described below in step D10*) will help you become familiar with nuclear morphologies, since not all tissue nuclei are oval or round in shape.*

c. After confirming that nuclei can be efficiently extracted as described above in step O3b, collect RNA from the following sources and extract it using the TRIzol method, as described in the Methods section "Assessment of RNA quality and DNA staining in nuclei extracted from three mouse organs after pH 3 or CuC lysis.":
    i. Crushed powder: Use as a control to assess the quality of total RNA in the source sample.
    ii. Nuclei after filtration through 800-mesh nylon: Take one-quarter of the filtrate and centrifuge at 1,000 × g for 1 minute. The resulting pellet represents nuclear RNA before purification.
    iii. Nuclei at the 30-60% iodixanol interface after density-gradient purification: Take half of the resuspended nuclei from step D9 (before on-column

washes), add PVA to a final concentration of 0.1%, and centrifuge at 1,000 × g for 1 minute. This pellet represents nuclear RNA after purification.

iv. Nuclei after on-column wash: Resuspend the nuclei in 200 µL of tissue lysis buffer without detergent, add PVA to a final concentration of 0.1%, and centrifuge at 1,000 × g for 1 minute. This pellet represents nuclear RNA quality after the entire procedure.

The eluted RNA can be directly analyzed for DV200 using an automated nucleic acid electrophoresis analyzer, and by RT-qPCR to assess cDNA enrichment compared with gDNA, as described in steps D3 and D3***.*

d. In-cell RT of methanol-fixed nuclei effectively verifies the presence of RNA in the extracted nuclei for USPPAR:
   i. Add PVA to the nuclei in methanol to achieve a final concentration of 0.1%.
   ii. Centrifuge at 1,000 × g for 1 minute and remove most of the supernatant.
   iii. Wash the pellet twice with 100 µL of low-salt buffer composed of 10 mM Tris-HCl (pH 8), 1 mM KCl, 0.1% PVA, 0.1 mM EDTA, and 5 ng/µL RNase inhibitor.
   iv. Prepare a 100-µL RT reaction containing 10 µL of 5,000 nuclei in low-salt buffer, 1X RT buffer, 0.5 mM of each dNTP, 1 µM T23VN primer, 0.1% PVA, 500 ng RNase inhibitor, and 500 ng of reverse transcriptase (use 0 ng reverse transcriptase for the gDNA-only control).
   v. Rotate at 42°C for 30 minutes.
   vi. Centrifuge at 1,000 × g for 1 minute and remove most of the supernatant.
   vii. Lyse the pellet with 25 µL of 0.2% Triton X-100 and 200 µg/mL proteinase K.
   viii. After adding the lysis buffer, vortex the pellet at 1,200 RPM at 55°C for 30 minutes.
   ix. Use the lysates for qPCR with cDNA and gDNA primers. Typically, 2-3 µL containing 300-500 nuclei per 20 µL qPCR reaction is sufficient. Include 0.5 mM PMSF (as used for TdT tailing in step A3) in the qPCR reaction to inactivate proteinase K. The RT sample with reverse transcriptase should show lower Ct values with the cDNA primers compared to the control without the reverse transcriptase.

e. Once all preliminary steps are successful, perform the official USPPAR experiment on liver nuclei. For the Barcoding stage, conduct three rounds of 48 × 48 × 48 well reactions as with cells. For the Amplification stage, use two separate 3,000-nucleus aliquots and process each in independent Amplification reactions. Processing separately prevents barcode collisions: combining 6,000 nuclei in one reaction at low multiplicity (48 × 48 × 48) risks assigning identical barcodes to different nuclei, making them indistinguishable.

Perform parameter validation during the reverse transcription step, as exemplified in the table below. (The table serves only as an example; users can adjust parameters as needed for their own samples.) Use the optimized amount of reverse transcriptase determined from cell-based tests (e.g., 100 ng for 2,000 cells is suitable). We recommend testing different dosages of RNase inhibitor at this stage, as nuclei isolated from tissues often contain higher levels of residual RNases. To verify successful Barcoding and Amplification, include HEK293T spike-in cells by adding them to the mouse nuclei after resuspending the nuclei and cells in low-salt buffer for the first wash.

**Row**	**Use [X] mouse liver cells per well in a 20 µL reaction.**	**Use [X] human methanol-fixed HEK293T cells per well in a 20 µL reaction as a spike-in.**	**Fixatives<br><br>None: methanol only**	**RNase inhibitor**
A	850	150	None	Commercial 5 U
B	850	150	None	Homebrew 100 ng
C	1,700	300	None	Commercial 5 U
D	1,700	300	None	Commercial 15 U
E	1,700	300	None	Homebrew 100 ng
F	1,700	300	None	Homebrew 300 ng
G	1,700	300	EGS	Homebrew 100 ng
H	1,700	300	EGS	Homebrew 300 ng

f. After obtaining successful results with liver nuclei, apply the same procedure (steps O3a-d) to the tissue of interest to determine whether RNA-containing nuclei can be extracted. Take into account potential variations or suboptimal conditions compared with the liver protocol. For example:

   i. If nuclei release is inefficient after pestling, as indicated by many remaining large fragments and few individual nuclei, consider using a Dounce homogenizer (see step D4**) or a stronger detergent, such as 0.3% Triton X-100 (see step D1**).

   ii. If RNA remains throughout the purification process, as determined by stepwise quality checks in O3c using DV200 and RT-qPCR, but the in-cell RT-qPCR fails to show enrichment of cDNA over gDNA in O3d, this suggests residual carryover of RNases in the nuclei. To further reduce RNase contamination, consider (i) replacing the original detergent combination with a stronger detergent (e.g., 0.3% Triton X-100; step D1**), (ii) performing two rounds of density-gradient purification (which still worked with USPPAR in our experience), (iii) using a lower number of cells for the RT step (e.g., from 2,000 to 1,000), or (iv) including yeast tRNA and

recombinant RNase inhibitor during purification (see step D1****). Alternatively, (v) lysine conjugators in methanol can be used to further inactivate RNases (see step D13*).

**Note that while the non-crosslinking ABF method performs well even with overnight treatment (-20 °C for 2 hours and -80 °C for the remaining time), prolonged use of the crosslinking reagent EGS can lead to clumping; therefore, extended EGS treatment should be avoided, or gentle pipetting at intervals should be performed to minimize clump formation.*

iii. If the presence of Ca^{2+} is undesirable, consider replacing $CaCl_2$ with EGTA (see D1***).

iv. For protease-rich tissues that may cause nuclei to become fragile and disintegrate during processing, consider including a protease inhibitor. The protease inhibitor can be removed during the wash step after density-gradient purification, and it remains compatible with the USPPAR procedure (see D1*****).

v. If many small fragments are present along with the nuclei after density-gradient purification, they may originate from cell walls or membrane-bound organelles. To remove RNA from these organelles, use a stronger detergent such as 0.3% Triton X-100 during lysis (step D1**). Small fragments and organelles can also be selectively removed by differential centrifugation. For example, replace on-column washes with a centrifugation step at 250 × g for 1 minute (step D9**). After centrifugation, carefully collect the supernatant just above the pellet to confirm that the nuclei have completely settled. A longer spin time can be used to achieve more complete pelleting, while a lower centrifugation force may be applied if gentler washing is desired.

g. If a small-scale pilot experiment (48 × 48 × 48) is successful with your target tissues, proceed to full-scale, high-multiplicity barcoding experiments (96 × 96 × 96) when more than 50,000 nuclei are required, as outlined in step O2c for the cells above.

If the procedure still does not work after all troubleshooting attempts, do not be discouraged. Please contact us with details of the steps you have taken during troubleshooting. Our goal is to make this open-source method as robust and broadly applicable as possible, and we are happy to assist in resolving any issues.

USPPAR Supporting Protocol

DISSOCIATION STAGE (1 hour)

**Use swing-bucket centrifuges to minimize nuclear loss throughout the procedure.*

***The CuC lysis system is preferred over the pH 3 lysis method for three key reasons. First, it does not exhibit the selective reduction of lymphocyte representation observed in spleen samples. Second, it is compatible with methanol fixation, which allows convenient sample storage without repeated freeze-thaw cycles. Methanol itself acts as a fixative and can also accommodate additional fixatives, such as ethylene glycol bis(succinimidyl succinate) (EGS), to provide enhanced fixation. Third, it exhibits RNase-inhibiting activity under neutral pH conditions, making it suitable for experimental manipulations that require a neutral environment.*

D1. Prepare the following buffers and keep them cold on ice.

Tissue lysis buffer with detergents

Component	Amount (ul)	Final Centration
Milli-Q water	882.8	-
HEPES-SO_4 pH 4.5, 2M	10	20 mM
NaCl, 5M	29.2	146 mM
$CaCl_2$, 1M***	1	1 mM
$MgCl_2$, 1M	21	21 mM
Tween 20, 20%**	5	0.1%
NP-40, 10%**	10	0.1%
Digitonin, 10%**	1	0.01%
Cu^{2+}-citrate complex pH 4.5, 250 mM	40	10 mM
Total	1000	-

**The CuC buffer was based on a standard lysis buffer (Tris-HCl pH 7.5, 20 mM NaCl, 146 mM $MgCl_2$, 21 mM $CaCl_2$) used by the Regev lab for snRNA-Seq[1]. Tris-HCl was replaced with HEPES because HEPES lacks primary amines that could interfere with crosslinking by formaldehyde or NHS esters. HEPES-SO_4 was prepared by adjusting the pH of the HEPES solution down to 4.5 using concentrated H_2SO_4.*

Although the Cu^{2+}-chelator complex is effective at inhibiting nucleases at pH levels ranging from 4.5 to 7.2, a relatively low pH of 4.5 was preferred to further reduce nuclease activity, as demonstrated in Fig 5C.

Here, Cu^{2+}-citrate complex was prepared by mixing equal molar amounts of $CuSO_4$ and sodium citrate acid in Milli-Q water, adjusting the pH to 4.5, and then adding water to reach a final concentration of 250 mM. Interestingly, a $CuSO_4$-to-citric-acid ratio of 1:0.5 provided even more potent RNase A inhibition (complete inhibition of 2 µg RNase A in a 20 µL reaction determined by the RNaseAlert assay.) without $Cu(OH)_2$ precipitation at pH levels above 7, even at concentrations tested up to 50 mM.

In our experience, the Cu^{2+}-citrate complex demonstrates flexible compatibility with various buffer ingredients. The inclusion of sucrose (250 mM) or the absence of NaCl and $CaCl_2$ does not affect its

nuclease-blocking potency. Thus, it should work with many other buffer formulations. With different formulations, it is recommended to assess compatibility by visually confirming the absence of $Cu(OH)_2$ precipitation (or through OD600 quantification) and verifying the inhibition of nuclease activity using RNaseAlert assays, as described in Fig 5 and the Methods section.

***The primary detergent composition (0.1% NP-40, 0.1% Tween 20, and 0.01% digitonin) is gentle, as it likely does not disrupt organellar membranes. This is supported by the presence of residual green chloroplasts after lysing maize shoots, and by the significantly reduced mitochondrial reads observed in ATAC-seq when using this detergent combination, compared with lysis using 0.1% IGEPAL CA-630 alone[2], which is chemically very similar to NP-40. In contrast, the ability of the nonionic detergent Triton X-100 to lyse chloroplasts indicates that it is a stronger lysis agent. Different detergents can significantly influence the composition of extracted cell types. Milder detergents, such as Tween 20, generally preserve higher cell-type diversity and retain more cytoplasmic content, including mature mRNAs[1]. However, these cytoplasmic carryovers often include unwanted organellar RNAs[1] and possible cytoplasmic nucleases. Furthermore, milder detergents may be less effective at releasing nuclei from cells embedded in dense cytoskeletal networks or extracellular matrices. Detergent composition in lysis buffers does not affect comparisons between samples processed with the same formulation; however, different detergent combinations can differentially recover cell types[1]. With the advantage of differential barcoding during the RT step, if maximum coverage of cell types is desired, the same tissue powder can be lysed using our standard recipe (0.1% NP-40, 0.1% Tween 20, and 0.01% digitonin) and compared with milder detergent (0.1% Tween 20 alone) or stronger detergent (0.3% Triton X-100) to identify the most suitable conditions. Key metrics to consider include the percentage of desirable cell types, minimal carryover of organellar reads, and UMI/gene counts per nucleus.*

****Regarding the use of salts in the lysis buffer, NaCl at this concentration helps reduce RNase activity[3], while $MgCl_2$ is important for maintaining chromatin and nuclear integrity. We found no specific reason to include Ca^{2+} in the lysis buffer. USPPAR is compatible with buffers lacking $CaCl_2$ and containing the Ca^{2+} chelator EGTA, as demonstrated in maize shoots. Even in animal tissues, snRNA-Seq has been successfully performed with $CaCl_2$ replaced by EGTA in our hands. Therefore, when the presence of $CaCl_2$ is undesirable, for example when lysing plant tissues rich in pectin, releasing nuclei from calcium-rich matrices such as bone, or preventing calcium-dependent enzymatic activity such as DNases, EGTA can be considered as a substitute for $CaCl_2$.*

*****The CuC in lysis buffer alone was able to inhibit RNA degradation (S9F Fig) and functioned effectively with nuclease-rich spleen tissue. The following extra additives have been tested for compatibility with USPPAR: RNase inhibitor (5 ng/μL) can be included during lysis and washes to sequester residual RNases during and after density-gradient centrifugation, although it can not stoichiometrically eliminate all RNase activity in RNase-rich tissues during lysis. Yeast tRNA (50 ng/μL) serves as a sacrificial competitive substrate for residual RNases, although the RNaseAlert assay showed it alone was not a potent RNase inhibitor.*

******For protease-rich tissues, Halt protease inhibitor is compatible at 1X concentration during lysis and density-gradient purification (up to step D8). It is removed during the washes in step D9*

to avoid confounding crosslinking or nonspecific binding to nuclei, which could inhibit downstream enzymatic reactions.

Iodixanol (30%) buffer

Component	Amount (µL)	Final Centration
Milli-Q water	388.8	-
HEPES-SO_4 pH 4.5, 2M	10	20 mM
NaCl, 5M	29.2	146 mM
$CaCl_2$, 1M	1	1 mM
$MgCl_2$, 1M	21	21 mM
Iodixanol, 60%	500	30%
PVA, 10%	10	0.1%
Cu^{2+}-citrate complex pH 4.5, 250 mM	40	10 mM
Total	1000	-

Tissue lysis buffer without detergents

Component	Amount (µL)	Final Centration
Milli-Q water	898.8	-
HEPES-SO_4 pH 4.5, 2 M	10	20 mM
NaCl, 5 M	29.2	146 mM
$CaCl_2$, 1 M	1	1 mM
$MgCl_2$, 1 M	21	21 mM
Cu^{2+}-citrate complex pH 4.5, 250 mM	40	10 mM
Total	1000	-

D2. Flash freeze the tissue fragments at liquid nitrogen temperature until use.

D3. **(Pulverization)** Crush the tissue fragments in folded polyimide films using an aluminum block on a stainless-steel plate stored in liquid nitrogen to maintain a low temperature.

**The aluminum block and stainless-steel plate can be kept in a liquid nitrogen tank. Place the wrapped tissue fragments between the block and plate, ensuring that the contact surfaces are flat for maximum crushing. To prevent contamination from spilled tissue powder, the folded tissue can be placed in a thin polyethylene (PE) plastic envelope before crushing. Next, use a hammer at room temperature to strike the aluminum block and crush the tissue. This procedure maintains the tissue at liquid nitrogen temperature throughout and minimizes direct contact of hands with the frozen metal surfaces. The resulting powder can be stored in a liquid nitrogen container for long-term storage, and a frozen microspatula can be used to take the required amounts for lysis as needed.*

***The RNA retention throughout the procedure can be assessed by directly lysing small aliquots of the powders or nuclei, followed by purification using a silica column (e.g., ZYMO, R2061), omitting DNase treatment, or using Qiagen mini-spin columns, as described in "Assessment of RNA quality and DNA staining in nuclei extracted from three mouse organs after pH 3 or CuC lysis" in the*

Methods section. RNA can be analyzed for DV200 using a capillary electrophoresis system to evaluate integrity.

****In addition to DV200, the comparison of RT reactions with reverse transcriptase (cDNA + gDNA) and without (gDNA-only) using qPCR, as shown in Fig 5E, is used to indicate the presence of RNA in the samples. Since the reverse transcription reaction is performed using oligo(dT) primers (T16V or T23VN), primers for target genes should be designed within 400 bp from the 3' end of the transcripts. There should be no splice sites within the amplicon or between the amplicon and the transcript ends to ensure efficient amplification of both genomic DNA (gDNA) and complementary DNA (cDNA). Note that gDNA is more difficult to elute from silica columns compared with RNA. Therefore, while the difference in Ct values between samples with and without reverse transcriptase can indicate the presence of RNA, it cannot provide reliable absolute RNA quantification by this comparison.*

D4. **(Lysis)** Immediately add 600 µL of ice-cold tissue lysis buffer containing detergents and grind with plastic pestles in microcentrifuge tubes to enhance dissociation.

**It is recommended to use less than 20 mg (approximately 2-4 scoops with a microspatula) of tissue powder per 600 µL of lysis buffer.*

***Plastic pestles used with microtubes provide a gentle method for grinding tissues while minimizing RNA exposure to nucleases compared with lengthy chopping. It is important to match the pestle to the bottom contour of the microtube and test several combinations to identify one that enables efficient dissociation of the tissue powder in fewer than 20 strokes, as indicated by the reduced coarse tissue fragments and increased fine sand-like material. Even with a well-matched set, more coarse fragments typically remain compared with those produced using a Dounce homogenizer, particularly when processing tissues with dense matrices or rigid cell walls. For tougher tissues, Dounce homogenization can be considered to enhance dissociation. However, unlike pestling, Dounce homogenization tends to be more damaging to nuclei. Excessive damage can be observed as increased thread- or worm-like debris at the 30-60% iodixanol interface after density-gradient centrifugation, likely representing chromatin released from ruptured nuclei. Although the extracted nuclei can still be used for snRNA-seq in our experience, there is concern about the selective loss of fragile nuclei from specific cell populations and the potential crosslinking of released chromatin fragments to intact nuclei, which could lead to contamination. Further, detergents should be added only after Dounce homogenization to minimize nuclear damage, as the combination of mechanical shear and detergent exposure can exacerbate membrane disruption.*

If it is necessary to determine the optimal method for isolating high-quality nuclei, the same tissue sample can be processed using pestling (20 strokes) and Dounce homogenization (5 and 20 strokes). The resulting lysates can then undergo parallel but separate purification, washing, and fixation steps. Following differential barcoding, the optimal method for a given tissue can be identified based on cell-type coverage and gene-detection sensitivity per barcode.

D5. Filter the lysates through an 800-mesh nylon membrane.

D6. **(Purification)** Layer the filtrate over 500 µL of iodixanol buffer, with 20 µL of 60% iodixanol already at the bottom as the cushion.

**Prepare 500 µL of iodixanol buffer first. Just before adding filtered nuclei, use a 20 µL pipette tip to take 60% iodixanol solution, insert the tip to the bottom of the iodixanol buffer, and slowly inject the 60% iodixanol cushion. Finally, gently add the filtered nuclei on top of the iodixanol buffer, taking care not to disturb the interface.*

D7. Centrifuge at 2,000 x g for 5 minutes.

D8. Collect the nuclei from the iodixanol 30%-60% interface using a pipette tip.

**When using CuC lysis buffer on the tested tissues, nuclei accumulate at the 30-60% iodixanol interface as a ring, because many nuclei slide along the tube wall before reaching that position. Take aliquots from several locations to check for nuclei: from the top of the 30% layer, at the bottom of the 60% layer, and within the 30% layer just above the 30-60% interface. Buoyant density of nuclei changes depending on treatment. For example, nuclei treated with DEPC have lower density and reside at the 30% interface. Nuclei lysed using CuC lysis buffer at pH 7.2 with Cu:Citrate ratio 1:0.5 exhibit higher density and accumulate at the bottom of the 60% layer. To account for differences in centrifugation speed between centrifuges (even when using standard RCF/g as reference), unsettled nuclei above the 30-60% interface indicate the need to increase centrifugation time to allow complete settling.*

D9. **(Wash)** Wash nuclei 2 times with 200 µL of Tissue lysis buffer without detergents in a wash bottle bearing hydrophilic PTFE membrane (3 µm pore size) using centrifugation at 100 × g for 1 minute .

**When collecting purified nuclei from the 30-60% interface, minimize the amount of iodixanol taken along with the nuclei. Dilute the nuclei with 200 µL of tissue lysis buffer without detergents for washing on a PTFE membrane in a wash bottle. The flow rate through the membrane may decrease due to clogging or high nuclei occupancy on the pores. If more than 10 µL of residual volume remains after 1 minute of centrifugation, perform pipetting and repeat the 1-minute centrifugation. If more than 10 µL of wash buffer remains after 2 minutes, switch to a new PTFE wash bottle. If a high number of nuclei is expected, they can be split across two or more wash bottles to ensure efficient washing.*

***Instead of washing nuclei on a PTFE membrane, perform two washes of plant nuclei by centrifugation at 250 × g for 1 minute each to reduce debris carryover (Include 0.05% PVA in the Tissue lysis buffer without detergents to prevent nuclei loss). Be aware that nuclear settling may be reduced due to increased iodixanol carryover from density-gradient purification, which can be observed as unsettled nuclei in the wash buffer just above the pellet. If this occurs, centrifuge the nuclei for a longer time to fully settle them, remove the supernatant containing residual iodixanol, and then perform two additional washes at 250 × g to remove debris.*

D10. **(Fixation)** Resuspend the nuclei in 50 µL of Tissue lysis buffer without detergents. Take 1 µL and dilute with 10 µL of Tissue lysis buffer without detergents to count for nuclei.

**For nuclei that are difficult to count with a phase-contrast microscope, such as those with irregular shapes or abundant cell-wall debris from plant tissues, add 2 µL of nuclei to 100 µL of*

phosphate-buffered saline (PBS) containing 10 µg/mL Hoechst 33258 and 0.1% PVA. Centrifuge at 1,000 × g for 1 minute, then resuspend the pellet to a final volume of 10 µL and count the nuclei using a fluorescence microscope.

D11. Add 10 volumes (500 µL) of ice-cold, freshly prepared methanol containing 5 mM $MgCl_2$ (final concentration), then transfer to -20°C to fix the nuclei.

**To minimize potential RNA degradation in washed nuclei, the methanol fixative is usually added immediately after taking the required amount of nuclei for counting, staining, or quality control.*

D12. After 30 minutes, record the number of nuclei in each sample and transfer the nuclei in methanol to a -80°C freezer for long-term storage.

Pause Point: These cells remain stable in methanol and can be stored at -80°C until downstream usage.

D13. (Optional) Take the required volume of nuclei to be fixed, add 1/9 volume of 100 mM EGS in DMSO, and incubate at 4 °C for 2 hours before proceeding to the Barcoding stage.

**Although methanol fixation alone was compatible with USPPAR, we recommend using selective lysine conjugators in methanol when additional crosslinking or RNase inactivation is desired. With paraformaldehyde (PFA) fixation, we and others[4] found that detection sensitivity decreased noticeably with small parameter changes, such as increasing the PFA concentration (0.4%) or performing fixation at room temperature. This reduction was likely caused by aldehyde groups covalently modifying RNA, which lowers reverse transcription efficiency. In addition, performing fixation in aqueous buffers increased the risk of ongoing RNA degradation.*

These issues were absent when using selective lysine conjugators in methanol. This approach also provides greater flexibility, as fixation time and temperature can be adjusted without concern for RNA degradation in aqueous conditions.

Among the conjugators, EGS crosslinks proteins and inactivates RNases, resulting in more stable nuclei. When equal numbers of nuclei were used for Barcoding, we consistently observed higher nuclear recovery after fixation with EGS compared to methanol alone or methanol plus ABF, indicating that increased crosslinking reduces nuclear loss during repeated incubations and centrifugations in the barcoding stage.

In contrast, ABF (4-azidobenzoyl fluoride) does not promote crosslinking and can be used in situations where crosslinking is undesirable-for example, to prevent carryover chromatin or organelle fragments from becoming attached to nuclei. Additionally, ABF is useful for downstream modifications, as it allows subsequent crosslinking with bivalent BCN or the addition of functional groups or tags using BCN derivatives[5].

USPPAR Supporting Protocol

BARCODING STAGE (4-5.5 hours)

Reverse Transcription (1st-Round Barcoding) (75 minutes)

B1. Take the required amounts of cells in methanol and add PVA (10% in Milli-Q water) to final 0.1%.

B2. Centrifuge at 1,000 x g for 1 minute.

B3. The pellets were washed 2 more times with 200 µL of a Low-salt buffer containing 5 ng/µL of Rat RNase inhibitor. (RNase inhibitor 5 ng/µL*200 µL*2)

**It is important to include EDTA in the wash solution to remove residual Cu^{2+}, which could inhibit downstream enzymatic reactions.*

***During the Barcoding procedure, multiple centrifugation-based washes are performed. Therefore, it is highly recommended to evaluate the recovery efficiency of cells or nuclei under the washing condition. This can be done by performing a mock 10-wash test using methanol-fixed samples: wash the sample ten times with 200 µL of the wash buffer and count the remaining cells or nuclei after the 5th and 10th washes.*
In our experience, recovery is generally good: typically >40% after ten washes for EGS-fixed hepatic nuclei (compared with >25% without EGS), and even higher for methanol-fixed HEK293T cells, corresponding to approximately >90% recovery per wash cycle.
Low recovery may result from insufficient centrifugal force, particularly for small nuclei or when the actual g-force differs from the indicated value. This issue can be confirmed if recovery improves after extending the centrifugation time.
To minimize sample loss due to adsorption, include polyvinyl alcohol (PVA) in both wash and reaction buffers at a final concentration of 0.05-0.1%. PVA serves as an effective anti-adsorption agent and is compatible with standard polypropylene tubes.
For nuclei, an additional strategy to improve recovery is EGS crosslinking, which enhances nuclear stability during washes. Alternatively, using a PTFE wash bottle combined with gentle centrifugation (100 × g) can avoid sample loss caused by surface adsorption.

****In our comparison of commercial recombinant RNase inhibitors with our homebrew version, we found that SUPERase·In was less effective than our in-house protein (Fig 7H). This result is consistent with information reported by 10x Genomics (https://kb.10xgenomics.com/s/article/360049543672-Can-I-use-an-alternative-RNase-inhibitor-part-number). One possible explanation is that the unit definition of SUPERase·In differs from that of other recombinant RNase inhibitors, which usually use the standard unit definition[6]. Based on a reviewer's suggestion and guidance from the 10x website, other recombinant RNase inhibitors, such as RNaseOUT and Protector, which use the standard unit definition, may be the preferred commercial options. As a reference, SPLiT-seq used 5 U of recombinant porcine RNase inhibitor (Enzymatics Y9240L, specific activity: 53,333 U/mg; available at https://www.qiagen.com/us/products/discovery-and-translational-research/enzymes-for-molecular-biology/rnase-inhibitor) in a 20 µL reaction, corresponding to approximately 93.75 ng of a 75 kDa protein per 20 µL reaction. In our protocol, we used 100 ng of a 96 kDa homebrew recombinant RNase inhibitor per 20 µL reaction. Thus, if a precise benchmark comparison with the above-mentioned commercial RNase inhibitor is desired, this*

conversion can serve as a reference point for normalizing RNase inhibitor performance based on the RNaseAlert assay (S2E Fig), keeping in mind that these recombinant RNase inhibitors act by stoichiometrically binding RNase in a 1:1 ratio. Based on the conversion here, when using commercial recombinant RNase inhibitors, we recommend starting with 0.25 U of commercial enzyme (e.g., the Enzymatics product that works in SPLiT-seq) for every 5 ng of our homebrew recombinant RNase inhibitor during the procedure. For a more precise assessment of the optimal RNase inhibitor amount, follow steps O2b and O3e. Since the key steps for RNase inhibitor function are during reverse transcription (RT) and the pre-RT wash (Fig 7H), differential amounts for comparison can be applied to these two steps. The same recommended amounts can then be used after RT to allow pooling of cells or nuclei for subsequent washes and ligation steps.

Nevertheless, we strongly recommend using our homebrew RNase inhibitor, available from Addgene, for the following reasons. It is easy to produce and purify using a single-step His-tag affinity column. It does not require refolding from inclusion bodies or cleavage from the solubility enhancing fusion partner, as the linked form is already active. In addition, it can be produced in large quantities from a single preparation.

B4. Resuspend the cells in 102 µL of the same wash buffer (RNase inhibitor 5 ng/µL*102 µL) to prepare 12 µL of the master mix below (102X volume) except pBC0T16V and pBC0N9:

Component	Amount (µL)	Final Centration	102 Wells per Plate
Milli-Q water	3.81	-	
Cells in Low-salt buffer	1	1-4,000 cells	
10X RT buffer	2	1X	
dNTP mix, 10 mM each	1	0.5 mM each	
PEG8K, 40%	3.75	7.5%	
PVA, 10%	0.1	0.05%	
pBC0T16V, 5 µM	4	1 µM	
pBC0N9, 5 µM*	4	1 µM	
Rat RNase inhibitor, 445 ng/µL	0.22	5 ng/µL	5 ng/µL*20 µL*102
M5 reverse transcriptase, 865 ng/µL**	0.12	5 ng/µL	5 ng/µL*20 µL*102
Total	20	-	

**pBC0N9 is optional but enhances gene detection sensitivity, particularly for non-polyadenylated genes. If pBC0N9 is omitted, replace it with 4 µL of Milli-Q water. Also, a random hexamer (N6) tail can be used instead of a nonamer (N9), because there was no difference in gene-detection sensitivity (S2D Fig), while using N9 increases oligonucleotide synthesis costs.*

***Use an RNase H-negative reverse transcriptase to prevent cleavage of the mRNA/cDNA hybrid, which would otherwise release cDNA from the cells.*

With commercial Maxima H Minus Reverse Transcriptase, use 50 to 200 units in a 20 µL reaction volume. In this volume, 50 units of the enzyme were sufficient for 1,000 RNA-abundant HEK293T cells (S2D Fig, lane 6). Using excessive amounts of the enzyme (e.g., 400

units in 20 µL) reduced sensitivity (Fig 7H, lane 9), likely due to inhibitory effects from components in the enzyme storage buffer.

B5. Add 12 µL of the master mix to a 96-well PCR plate containing 4 µL of pBC0T16V and 4 µL of pBC0N9.

B6. Rotate the whole plate at 42°C for 40 minutes in an oven.

**Based on a reviewer's suggestion, we found that incubating at 42°C for 20 minutes followed by 50°C for 20 minutes improved gene detection sensitivity in HEK293T cells (S2D Fig, lane 8). This approach may also be applicable to nuclei samples. To ensure success, we recommend performing an initial pilot test by differentially barcoding part of the sample at 42°C for 40 minutes and the other part at 42°C for 20 minutes followed by 50°C for 20 minutes. This ensures that there is no heat-induced nuclear clumping or loss of information due to differences in surface or internal properties between fixed nuclei and whole cells.*

B7. Stop the reaction by adding 0.3 µL of EDTA (0.5 M) to each well.

**We usually add 3.6 (0.3*12) µL of EDTA to each well of the first column, use multichannel pipettes to pool the contents of the 12 columns into the last column, and then pool them for centrifugation.*

<u>Ligation-Mediated Barcoding (2nd and subsequent rounds) (75 minutes each round)</u>

**We usually take the required amount (4 µL) of the barcoding adapters from the thawed stock solution and reanneal them using the identical annealing program to minimize issues related to freeze-thaw-induced duplex denaturation.*

B8. Pool the reactions into 4 or 8 microtubes and centrifuge at 1,000 x g for 1 minute.

**Centrifuging a large total volume (~2 mL) in 2 microtubes (~1 mL each) from a 96-well plate usually leads to higher cell loss. It is recommended to split the volume into 8 tubes of 250 µL each to minimize this loss. Additionally, washing the samples can be done using 2 wash bottles with 3 µm pore size PTFE membranes, similar to the procedure used for nuclei after iodixanol density gradient centrifugation in step D9 of the Dissociation stage.*

B9. Pool the 4 or 8 pellets into two separate microtubes, each containing 200 µL of Low-salt buffer with 0.5 ng/µL of Rat RNase inhibitor. (RNase inhibitor 0.5 ng/µL*200 µL*2)

B10. Centrifuge at 1,000 × g for 1 minute.

B11. Wash the pellet again using the same volume of the wash buffer and centrifuge at 1,000 × g for 1 minute. (RNase inhibitor 0.5 ng/µL*200 µL*2)

B12. Resuspend the pellets in 102 µL of the same wash buffer (RNase inhibitor 0.5 ng/µL*102 µL) and prepare 16 µL of the master mix below, excluding the Barcoding adapters.

Component	Amount (µL)	Final Centration	102 Wells per Plate
Milli-Q water	12.53	-	
Cells in Low-salt buffer	1	4,000 cells	
10X PNK buffer	2	1X	
PVA, 10%	0.2	0.1%	
Barcoding adapters, 5 µM	4	1 µM	
Rat RNase inhibitor, 445 ng/µL	0.02	0.5 ng/µL	5 ng/µL*20 µL*102
T4 DNA ligase, 320 ng/µL*	0.25	4 ng/µL	4 ng/µL*20 µL*102
Total	20	-	

**If commercial T4 DNA ligase is used, the optimal amount can be determined by performing a ligation followed by denaturing gel electrophoresis, as described in Supporting Protocol 1 (steps 3–6). Another way to verify the ligase levels in a more system-relevant context is to perform differential barcoding using varying amounts of T4 DNA ligase across different rows or columns now and analyze gene detection in the sequencing data.*

B13. Add 16 µL of the master mix to a 96-well PCR plate containing 4 µL of the Barcoding adapters.

B14. Rotate the entire plate at 37°C for 40 minutes in an oven.

B15. Stop the reaction by adding 0.5 µL of 0.5 M EDTA to each well.

**We usually add 6 (0.5*12) µL of EDTA to each well of the first column, use multichannel pipettes to pool the contents of the 12 columns into the last column, and then pool them for centrifugation.*

B16. Repeat steps B9-15 for subsequent rounds of ligation-mediated barcoding.

Aliquot Barcoded Cells for Long-Term Storage (20 minutes)

B17. After the last round of ligation, pool the reactions into 4 or 8 microtubes and centrifuge at 1,000 x g for 1 minute.

B18. Pool the 4 or 8 pellets with 200 µL of Low-salt buffer with 0.5 ng/µL of Rat RNase inhibitor. (RNase inhibitor 0.5 ng/µL*200 µL)

B19. Filter the resuspended pellet through an 800-mesh nylon membrane (S2M Fig) pre-wetted with 100 µL of the same buffer. (RNase inhibitor 0.5 ng/µL*100 µL)

B20. Wash the column again with 200 µL of the same buffer. (RNase inhibitor 0.5 ng/µL*200 µL)

B21. Pool filtrates and split into 2 microtubes.

B22. Pellet the cells/nuclei by centrifugation at 1,000 x g for 1 minute.

B23. Wash the pellets again with 200 µL of the same buffer by centrifuging at 1,000 × g for 1 minute. (RNase inhibitor 0.5 ng/µL*200 µL)

B24. Resuspend both pellets in a total of 200 µL of the same buffer. (RNase inhibitor 0.5 ng/µL*200 µL)

B25. Count the cells/nuclei using a hemocytometer.

**Typically, 160,000 to 320,000 cells or nuclei are recovered from an input of 400,000, corresponding to a yield of 40-80% after the entire Barcoding procedure.*

B26. Centrifuge at 1,000 x g for 1 minute in one microtube.

B27. Resuspend the pellet in an appropriate amount of the wash buffer, and aliquot (~10,000-20,000 cells in 3 µL per PCR tube) for long-term storage at -80°C. (RNase inhibitor 0.5 ng/µL*3 µL *30)

**It is important to use Low-salt buffer and avoid other buffers containing unnecessary salts, such as additional K^+, Na^+, or Mg^{2+}, when aliquoting samples. These ions can be inhibitory for efficient TdT tailing as shown in Fig 2.*

Pause Point: These cells can be stored at -80°C for long-term storage.

USPPAR Supporting Protocol

AMPLIFICATION STAGE (5.5+3 hours)

<u>Lysis and Poly(dA)-Tailing of cDNA (150 minutes)</u>

A1. Add 8 µL of lysis buffer containing Triton X-100 (0.275%) and proteinase K (275 µg/mL) to the cells in 3 µL of Low-salt buffer to initiate lysis.

Component	Amount (µL)	Final Centration
Cells/nuclei in Low-salt buffer	3	10,000-20,000
Triton X-100, 0.275%; proteinase K, 275 µg/mL	8	Triton X-100, 0.2%; Proteinase K, 200 µg/mL
Total	11	-

A2. Vortex at 1,500 rpm at 55°C for 50 minutes.

Pause Point: This lysate can be stored at -80°C until downstream usage.

⑤ A3. Prepare the following polydeoxyadenylation mixture:

Component	Amount (µL)	Final Centration	For 10,000 cells*10
Milli-Q water	0.8	-	
Cell lysate	11	10,000-20,000 cells	
10X TdT buffer	2	1X	
dATP, 20 mM	2	2 mM	
$CoCl_2$, 7.5 mM	2	0.75 mM	
PMSF in DMSO, 50 mM	0.2	0.5 mM	
TdT, 20 U/µL*	2	2 U/µL	2 µL*10
Total	20	-	

A4. Incubate at 37°C for 60 minutes and at 42°C for 10 minutes, followed by heat inactivation at 75°C for 20 minutes.

**Sufficient tailing is critical not only for adding PCR primer binding sites to cDNA but also for inactivating unused residual ligation adapters and RT primers during the Barcoding stage. Therefore, it is essential to use enough TdT to achieve adequate tailing. A straightforward way to verify TdT sufficiency is to use a spare lysate and add 1-2 pmol (1-2 µL of 1 µM stock) of annealed 3'-Recessed Duplex DNA as a surrogate reporter to perform TdT tailing, as shown in Fig 2F.*

In this assay, the duplex DNA is incubated with the lysate and TdT, and the resulting tailing is compared with negative controls: the annealed 3'-Recessed Duplex DNA alone (to show the precise location of untailed oligos) and the duplex DNA incubated with lysate, but without TdT. After denaturing gel electrophoresis and silver staining, as detailed in the Methods section, the completeness of shifting of the DNA (indicated by the absence of residual oligos at the original position) and the degree of upshifting (reflecting extension length) is expected to reflect the efficiency of tailing of both the cDNA and any unreacted oligos in the lysate.

A5. Add 1 µL of EDTA (16 mM) to obtain trace-free, fully extended cDNA.

Pause Point: This tailed cDNA can be stored at -80°C until downstream usage.

***Successful reverse transcription and ligation can be assessed by performing qPCR using primers that target housekeeping gene cDNA (e.g., PSMB4) and genomic DNA, with the extended cDNA serving as the template. The cDNA primer set should yield significantly more copies than the gDNA set, evident in three key primer combinations: PSMB4 F+R (successful RT), PSMB4 F + supAGC, the primer targeting the last-round barcoding adapter (successful ligation), and PSMB4 R + T35 (successful polydeoxyadenylation). These combinations serve as markers for each critical step.**

Single-Tube 2nd-Strand Synthesis and Preamplification (120 minutes)

⑥ A6. Prepare the following reaction mixture on ice:

Component	Amount (μL)	Final Centration
Milli-Q water	31.4	-
cDNA with poly(dA) tails	21	10,000-20,000 cells
HAPA HiFi HF buffer, 5X	16	1X
dNTP, 10 mM each	1.6	0.2 mM
supT25*V, 2 μM	8	200 nM
Total	78	-

A7. Split the mixture into two 39 μL portions, heat each at 94°C for 1 minute, and then maintain at 45°C.

A8. Add 1 μL of KAPA HiFi to each reaction mixture. (KAPA HiFi 2 μL*10)

A9. Conduct 54 cycles of 0.5°C increase for 20 seconds each cycle, followed by incubation at 72°C for 10 minutes to synthesize 2nd-strand cDNA, and then maintain at 4°C indefinitely.

⑦ A10. Prepare the preamplification mixture:

Component	Amount (μL)	Final Centration	For 10,000 cells*10
Milli-Q water	36.4	-	
HAPA HiFi HF buffer, 5X	16	1X	
dNTP, 10 mM each	1.6	0.2 mM	
Suppressive primer, 5 μM	16	1 μM	
DMSO, 100%	8	10%	
KAPA HiFi, 1U/μL	2	2 U/80 μL	2 μL*10
Total	80	-	

A11. Add the preamplification mixture to the 2nd-strand cDNA from step A9.

A12. Perform thermocycling with an initial denaturation at 98°C for 1 minute, followed by 12 cycles of denaturation at 98°C for 10 seconds, annealing at 68°C for 20 seconds, and extension at 72°C for 4 minutes.

**Based on our experience, 12 cycles are usually sufficient. To further avoid insufficient or excessive amplification, 5 μL of the second-strand cDNA of step A9 can be used to perform a pilot qPCR with*

SYBR. The goal is to achieve a cycle number that reaches 20-50% of maximal raw fluorescence while ensuring that the amplification curve remains in the exponential phase.

A13. Stop polymerase activity by adding 0.96 µL of 500 mM EDTA to the reaction mixture.

Pause Point: This preamplified cDNA can be stored at -80°C until downstream usage.

Purification and Quantification of cDNA (60 minutes)

A14. Prepare purification mixture:

Component	Amount (µL)	Final Centration
Preamplified cDNA	~160	-
PEG/NaCl solution*	86.7	~7% of PEG8000
carboxylated paramagnetic beads, 5%	1	-
Total	247.7	-

**It is important that the size selection selectively enriches the desired range of amplified DNA. After preparing a new homebrew PEG/NaCl solution, it is recommended to identify the optimal final PEG concentration that removes empty and short amplicons to account for batch variations. One approach is to split preamplified DNA from a real lysate and purify it with different final PEG8000 concentrations (e.g., 6.5%, 7%, 7.5%, and 8%), then analyze the eluents using a TapeStation or Qsep system. However, this method consumes both sample and time.*

A simpler approach is to use a DNA ladder with a dense distribution (e.g., 100, 200, 300, 400, 500 bp up to 1 kb) prepared in the reaction formulation identical to those used for amplifying DNA (including PCR buffer, enzyme, DMSO, etc., but without thermocycling or DNA templates) and purify it using the same range of PEG concentrations as described above. By comparing with the unpurified original ladder using a TapeStation or Qsep system, this assay allows rapid and clear determination of the PEG cutoff that defines the desired size range. Once established, this information can be readily applied to real samples and subsequent two-sided size selection.

A15. Incubate the mixture at ambient temperature for 10 minutes.
A16. Place the tube on a magnetic stand and let it stand for an additional 5 minutes.
A17. Remove the supernatant.
A18. Wash the beads with 500 µL of freshly prepared ethanol-water (80%) by inverting the tubes horizontally 10 times on the magnetic stand.
A19. Repeat steps A17 and A18 once more.
A20. Remove most of wash solution and leave the beads on the magnetic stand for 5 minutes to allow the remaining ethanol to evaporate.
A21. Add 8 µL of loTE to the beads, pipette to mix, and leave the mixture at ambient temperature for 10 minutes.
A22. Place the tube on a magnetic stand and take the supernatant containing the preamplified cDNA.

**A tiny amount (e.g., 0.25 µL) of eluted preamplified DNA can be assayed by qPCR using the cDNA and gDNA primers. A relative increase in housekeeping cDNA amplicons (lower Ct values) compared with gDNA amplicons indicates successful progression through the Barcoding stage (cDNA synthesis and barcode ligation to add one PCR binding site) and the Amplification stage (poly-dA tailing for second-strand synthesis to add the second PCR binding site on cDNA for suppressive PCR).*

A23. Assay dsDNA concentration in the eluent using the following mixtures:

dsDNA type	dsDNA volume (µL)	loTE containing 1X diluted SYBR Green I (µL)	Purpose
Plasmid DNA, 60 ng/ul in loTE	3.2	220.8	For standard curve
Plasmid DNA, 30 ng/ul in loTE	3.2	220.8	
Plasmid DNA, 15 ng/ul in loTE	3.2	220.8	
Plasmid DNA, 5 ng/ul in loTE	3.2	220.8	
Plasmid DNA, 2 ng/ul in loTE	3.2	220.8	
loTE only	3.2	220.8	
Eluted DNA	1	69	Sample to test

A24. Quantify the eluted preamplified cDNA using SpectraMax iD5 fluorescence reader (PicoGreen program) with a calibration curve produced by plasmid DNA.

**Triplicates of 70 µL per concentration are used in 384-well plates for the standard curve, while only one well is used for the eluted cDNA to conserve the samples.*

It is critical that the fluorescent reading of the eluted cDNA falls within the linear region of the plasmid DNA standard curve. If it does not, try diluting the sample and repeat the quantification.

cDNA Tagmentation (45 minutes)

⑧ A25. Prepare tagmentation mixture:

Component	Amount (µL)	Final Centration	For 10,000 cells*10
Milli-Q water	6.5-X	-	
Plasmid carrier, 50 ng/µL	1	50 ng/10 µL	
Preamplified cDNA	X	50 ng/10 µL	
10X Tagmentation buffer	1	1X	
DMF, 100%	1	10%	
Loaded Tn5, 2µM*	0.5	0.1 µM	0.5 µL*10
Total	10	-	

**The optimal amount of loaded Tn5 is determined in "Support Protocol 2: Assemble and test Tn5 transposase complex".*

A26. Incubate at 55°C for 30 minutes.

A27. Stop the reaction by adding 2.5 µL of the 5X Quenching buffer and heating the reaction at 55°C for an additional 10 minutes.

Pause Point: This tagmented cDNA can be stored at -80°C until downstream usage.

Library Amplification (75 minutes)

⑨ A28. Prepare the pilot qPCR mixture:

Component	Amount (µL)	Final Centration	For 10,000 cells*10
Milli-Q water	3.475	-	
Tagmented cDNA	1	-	
HAPA HiFi HF buffer, 5X	2	1X	
dNTP, 10 mM each	0.2	0.2 mM	
supAGC, 2 µM	1	200 nM	
i5, 2 µM	1	200 nM	
SYBR Green I diluted to 1,500 fold in DMSO	0.5	1/30,000	
ROX 10 µM	0.5	500 nM	
Triton X-100, 10%	0.2	0.1%	
KAPA HiFi, 1 U/µL	0.125	0.125 U/10 µL	0.125 µL*10
Total	10	-	

A29. Perform thermocycling with an initial denaturation at 98°C for 1 minute, followed by 20 cycles of denaturation at 98°C for 10 seconds, annealing at 63°C for 15 seconds, and extension at 72°C for 30 seconds.

A30. Prepare the official PCR mixture:

Component	Amount (µL)	Final Centration	For 10,000 cells*10
Milli-Q water	49.4	-	
Tagmented cDNA	11.5	-	
HAPA HiFi HF buffer, 5X	24	1X	
dNTP, 10 mM each	2.4	0.2 mM	
P7supAGC or P7NextR2supAGC, 2 µM	12	200 nM	
Nextera i5XX, 2 µM	12	200 nM	
DMSO, 100%	6	5%	
Triton X-100, 10%	1.2	0.1%	
KAPA HiFi, 1 U/µL	1.5	0.125 U/10 µL	1.5 µL*10
Total	120	-	

A31. Perform thermocycling with an initial denaturation at 98°C for 1 minute, followed by cycles of denaturation at 98°C for 10 seconds, annealing at 63°C for 15 seconds, and extension at 72°C for 30 seconds.

**Use the cycle number at which the raw fluorescence reaches approximately half of the maximum in the pilot qPCR from step A29.*

A32. Add 0.72 µL of EDTA (0.5 M) to achieve a final concentration of 3 mM.

Pause Point: This amplified library can be stored at -80°C until downstream usage.

Size-selection for the library (60 minutes)

A33. Prepare purification mixture.

Component	Amount (µL)	Final Centration
Preamplified cDNA	~120	-
PEG/NaCl solution*	45.9	~5.5% of PEG8000
carboxylated paramagnetic beads, 5%	1	-
Total	166.9	-

**For the NovaSeq X Plus system, we limited the upper size cutoff of our library to 800 bp (the optimal PEG concentration for this cutoff was determined in step A14*). Longer DNA fragments are at a competitive disadvantage during detection, especially when libraries are pooled and sequenced in the same lane with others containing shorter fragments. This imbalance can result in reduced sequencing output for libraries enriched in longer fragments.*

A34. Incubate the mixture at ambient temperature for 10 minutes.
A35. Place the tube on a magnetic stand and let it stand for an additional 5 minutes.
A36. Take the supernatant add PEG/NaCl solution:

Component	Amount (µL)	Final Centration
Supernatant	~160	-
PEG/NaCl solution**	19	~7% of PEG8000
carboxylated paramagnetic beads, 5%	1	-
Total	180	-

**There will be some loss of supernatant, so adjust the amounts of PEG/NaCl solution accordingly.*

***The final concentration of PEG8000 is determined by the expected library size. With 4-round barcoding, where the empty library is expected to be 259 bp in size, a concentration of 7% will be sufficient. For smaller libraries with fewer barcoding rounds and different library structures, the optimal PEG8000 concentration will be different. The PEG concentrations corresponding to*

different DNA size cutoffs, as determined in step A14, will be used here for the final PEG concentration.*

A37. Incubate the mixture at ambient temperature for 10 minutes.

A38. Place the tube on a magnetic stand and let it stand for an additional 5 minutes.

A39. Discard the supernatant.

A40. Wash the beads with 500 µL of freshly prepared ethanol-water (80%) by inverting the tubes horizontally 10 times on the magnetic stand.

A41. Repeat steps A39 and A40 once more.

A42. Remove most of wash solution and leave the beads on the magnetic stand for 5 minutes to allow the remaining ethanol to evaporate.

A43. Add 8 µL of loTE containing 0.1% Tween 20 to the beads, pipette to mix, and leave the mixture at ambient temperature for 10 minutes.

**The inclusion of Tween 20 minimizes the absorption of DNA onto plastic tubes during shipping at ambient temperature.*

A44. Place the tube on a magnetic stand and take the supernatant for NGS.

Pause Point: This amplified library can be stored at -80°C until downstream usage.

**Part of the eluted libraries can be submitted for fragment analysis using a Qsep or similar instrument to confirm that the library is in the correct size range, with essentially all fragments between "empty library + 50 bp" and 800 bp.*

***When pooling multiple libraries, each individual library should be quantified using Illumina P7 (CAAGCAGAAGACGGCATACGAGAT) and P5 (AATGATACGGCGACCACCGAGATCTACAC) primers (identical conditions to steps A28-29, except for the different primers and the use of 0.25 µL of the eluted library as the template). The volumes of each sublibrary in the pool should be determined based on their amplification curves to ensure that each sublibrary contributes a similar amount of template to the final pool. Meanwhile, primers targeting cDNA and gDNA can also be used in qPCR to demonstrate the selective enrichment of cDNA over gDNA as an additional layer of library quality control, as described in step A22*.*

[1] M. Slyper *et al.*, "A single-cell and single-nucleus RNA-Seq toolbox for fresh and frozen human tumors," *Nat Med*, vol. 26, no. 5, pp. 792–802, May 2020, doi: 10.1038/s41591-020-0844-1.

[2] M. R. Corces *et al.*, "An improved ATAC-seq protocol reduces background and enables interrogation of frozen tissues," *Nat Methods*, vol. 14, no. 10, pp. 959–962, Oct. 2017, doi: 10.1038/nmeth.4396.

[3] C. Park and R. T. Raines, "Origin of the 'inactivation' of ribonuclease A at low salt concentration," *FEBS Lett*, vol. 468, no. 2–3, pp. 199–202, Feb. 2000, doi: 10.1016/S0014-5793(00)01227-8.

[4] A. Sziraki *et al.*, "A global view of aging and Alzheimer's pathogenesis-associated cell population dynamics and molecular signatures in human and mouse brains," *Nat Genet*, vol. 55, no. 12, pp. 2104–2116, Dec. 2023, doi: 10.1038/s41588-023-01572-y.

[5] I. Dovgan *et al.*, "Acyl Fluorides: Fast, Efficient, and Versatile Lysine-Based Protein Conjugation via Plug-and-Play Strategy," *Bioconjug Chem*, vol. 28, no. 5, pp. 1452–1457, May 2017, doi: 10.1021/acs.bioconjchem.7b00141.

[6] P. Blackburn, "Ribonuclease inhibitor from human placenta: rapid purification and assay.," *Journal of Biological Chemistry*, vol. 254, no. 24, pp. 12484–12487, Dec. 1979, doi: 10.1016/S0021-9258(19)86340-8.
