# Supplementary figures and images for: USPPAR is a cost-effective, scalable, and highly sensitive single-cell RNA sequencing workflow compatible with diverse specimens
Source: PLoS Biol. 2025 Dec 15;23(12):e3003537. doi: 10.1371/journal.pbio.3003537 (PMC12704895; doi:10.1371/journal.pbio.3003537)

Figure 2

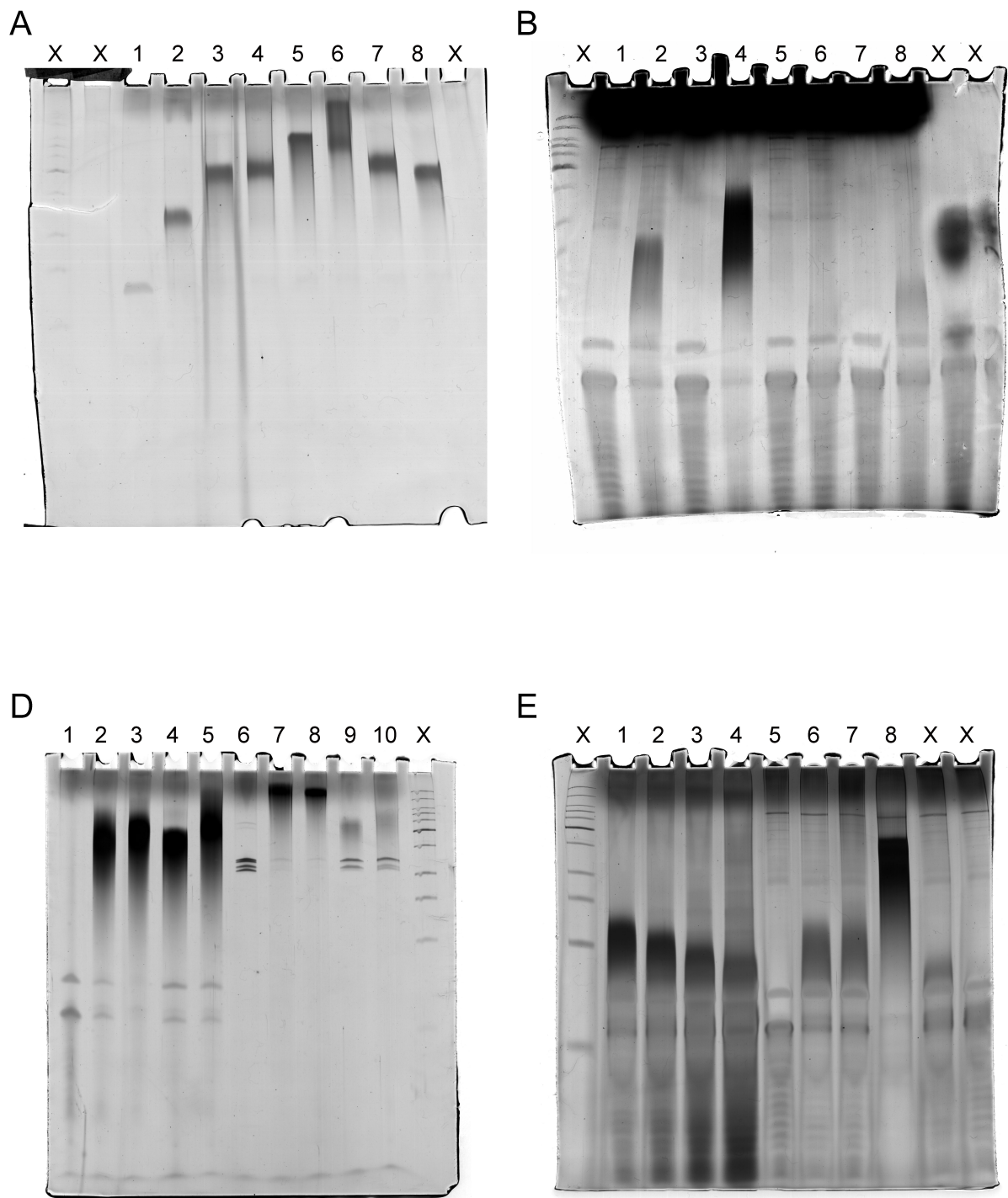

Figure 4

B

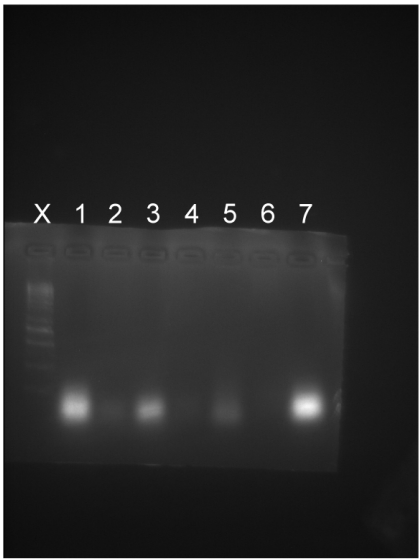

Figure S2

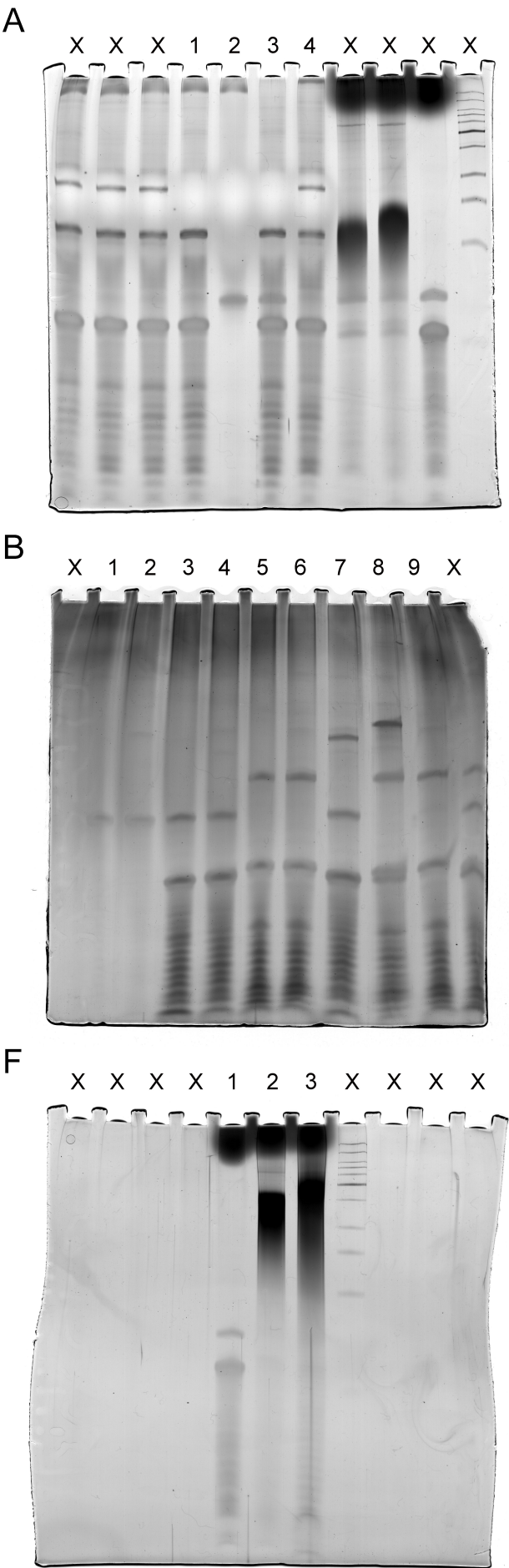

Figure S3

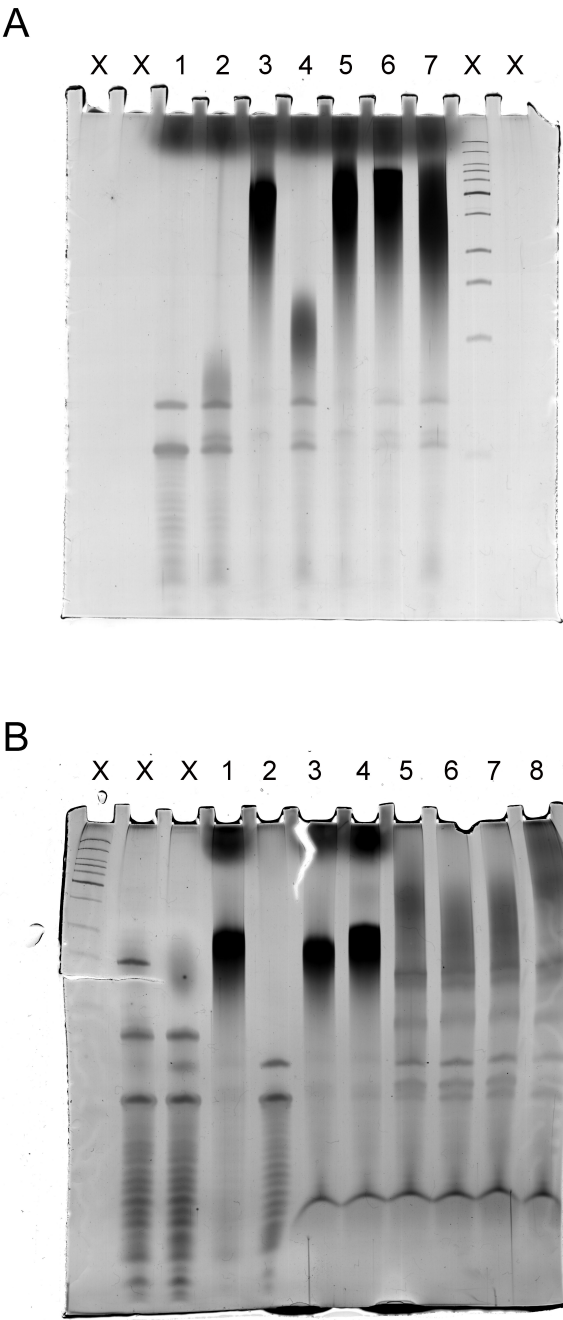

Figure S12

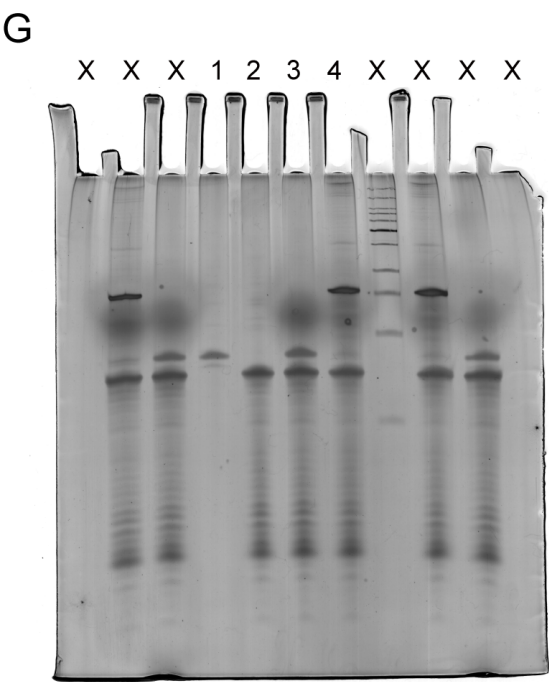

Supplement: S1 Raw Images — (PDF) [file pbio.3003537.s022.pdf]
